# Factors Correlated With Physical Function 1 Year After Total Knee Arthroplasty in Patients With Knee Osteoarthritis: A Systematic Review and Meta-analysis

**DOI:** 10.1001/jamanetworkopen.2022.19636

**Published:** 2022-07-11

**Authors:** Unni Olsen, Maren Falch Lindberg, Christopher Rose, Eva Denison, Caryl Gay, Arild Aamodt, Jens Ivar Brox, Øystein Skare, Ove Furnes, Kathryn Lee, Anners Lerdal

**Affiliations:** 1 Department of Nursing Science, Institute of Health and Society, Faculty of Medicine, University of Oslo, Oslo, Norway; 2 Department of Orthopaedic Surgery, Lovisenberg Diaconal Hospital, Oslo, Norway; 3 Division for Health Services, Norwegian Institute of Public Health, Oslo, Norway; 4 Department of Family Health Care Nursing, University of California, San Francisco; 5 Department of Patient Safety and Research, Lovisenberg Diaconal Hospital, Oslo, Norway; 6 Department of Physical Medicine and Rehabilitation, Oslo University Hospital, Oslo, Norway; 7 Institute of Clinical Medicine, Faculty of Medicine, University of Oslo, Oslo, Norway; 8 Norwegian Arthroplasty Register, Department of Orthopaedic Surgery, Haukeland University Hospital, Bergen, Norway; 9 Department of Clinical Medicine, University of Bergen, Bergen, Norway; 10 Department of Interdisciplinary Health Sciences, Institute of Health and Society, Faculty of Medicine, University of Oslo, Oslo, Norway

## Abstract

**Importance:**

More than 1 in 5 patients do not experience improved physical function after total knee arthroplasty (TKA). Identification of factors associated with physical function may be warranted to improve outcomes in these patients.

**Objective:**

To identify preoperative and intraoperative factors associated with physical function at 12 months after TKA in a systematic review and meta-analysis.

**Data Sources:**

Data from January 2000 to October 2021 were searched in Medline, Embase, Cumulative Index to Nursing and Allied Health Literature (CINAHL), Cochrane Library, and Physiotherapy Evidence Database (PEDro). No language restrictions were applied.

**Study Selection:**

Prospective observational studies or randomized clinical trials on factors associated with physical function after TKA in adult patients with osteoarthritis were selected. A prespecified peer-reviewed protocol was followed.

**Data Extraction and Synthesis:**

Following the Preferred Reporting Items for Systematic Reviews and Meta-analyses guideline, 2 reviewers independently screened titles and abstracts and judged risk of bias using Quality in Prognosis Studies (QUIPS). Multivariate random-effects meta-analyses were performed to estimate mean correlations between factors and physical function with 95% CIs. Sensitivity analyses were conducted for each QUIPS domain. Certainty of evidence was evaluated using Grading of Recommendations, Assessment, Development and Evaluations (GRADE). This study was registered with the International Prospective Register of Systematic Reviews (PROSPERO).

**Main Outcomes and Measures:**

The primary outcome was physical function 12 months after TKA. Secondary outcomes were physical function 3 and 6 months after TKA. All estimates are mean correlations between factors and postoperative function. Positive correlations correspond to better function.

**Results:**

Among 12 052 articles, 20 studies (including 11 317 patients and 37 factors) were analyzed. Mean correlation with higher BMI was estimated to be −0.15 (95% CI, −0.24 to −0.05; *P* = .33; moderate-certainty evidence), while mean correlation with better physical function was estimated to be 0.14 (95% CI, 0.02 to 0.26; *P* = .03; low-certainty evidence) and mean correlation with more severe osteoarthritis was estimated to be 0.10 (95% CI, 0.01 to 0.19; *P* = .17; high-certainty evidence). In sensitivity analyses, mean correlation with better physical function was estimated to be 0.20 (95% CI, 0.04 to 0.36; *P* = .02), and so perhaps a larger coefficient than in the main analysis, while mean correlations were estimated to be similar for other factors (BMI: –0.17; 95% CI, –0.28 to –0.06; *P* < .001; osteoarthritis severity: 0.10; 95% CI, −0.01 to 0.20; *P* = .05).

**Conclusions and Relevance:**

This study found that higher presurgical BMI was correlated with worse physical function (with moderate certainty) and that better physical function (low certainty) and osteoarthritis severity (high certainty) were correlated with better physical function after TKA. These findings suggest that these factors should be included when testing predictive models of TKA outcomes.

## Key Points

**Question:**

What preoperative and intraoperative factors are correlated with physical function after total knee arthroplasty (TKA)?

**Findings:**

In this systematic review and meta-analysis of 20 studies that included 11 317 patients with osteoarthritis, higher preoperative body mass index (BMI) was correlated with worse physical function, while better preoperative physical function and more severe osteoarthritis were correlated with better physical function 1 year after TKA.

**Meaning:**

These findings suggest that presurgical BMI, physical function, and osteoarthritis severity may be important factors to include and test in models predicting TKA outcomes.

## Teaser

This systematic review and meta-analysis investigates the correlation of preoperative and intraoperative factors with physical function 1 year after total knee arthroplasty in patients with knee osteoarthritis.

## Introduction

Total knee arthroplasty (TKA) has become the third most common inpatient surgery in the United States, with 750 000 yearly procedures projected to double in the next decade.^1,2^ TKA is regarded as a cost-efficient and effective treatment for restoring physical function in patients with end-stage osteoarthritis.^3^ However, more than 1 in 5 patients do not regain physical function after TKA.^4^ Nonimprovement of physical function is a risk factor associated with more expensive revision surgery and an immense burden at individual, health care system, and socioeconomic levels.^5,6^

Factors identified in predictive models using high-quality evidence could improve patient outcomes, particularly for those who are unlikely to benefit from surgery or who have unrealistic expectations. Evidence on factors associated with physical function has been reviewed previously, but findings were contradictory, limited in scope, based on pooled data across short-term and longer-term outcomes, or did not address certainty of evidence.^7,8,9,10,11,12,13^ Thus, there is need for a new synthesis of evidence on short-term TKA outcomes that uses current systematic review methods and captures recently published studies. The aim of this systematic review and meta-analysis was to synthesize evidence on preoperative and intraoperative factors associated with physical function 12 months after TKA (primary outcome) and 3 and 6 months after TKA (secondary outcomes).

## Methods

In this systematic review and meta-analysis, we followed a prespecified peer-reviewed protocol^14^ and a preprint^15^ registered in International Prospective Register of Systematic Reviews (PROSPERO; CRD42018079069), designed and conducted according to Cochrane Handbook guidelines.^16^ Results are reported according to the recently revised Preferred Reporting Items for Systematic Reviews and Meta-analyses (PRISMA) reporting guideline.

### Search Strategy and Data Sources

The search strategy was collaboratively developed by researchers (U.O. and M.F.L.) and research librarians, with feedback from the research team.^14^ Published studies from January 1, 2000, to October 8, 2021, were systematically searched, with no language restrictions, in Medline (Ovid), Embase (Ovid), Cumulative Index to Nursing and Allied Health Literature (CINAHL; EBSCO), Cochrane Library, and Physiotherapy Evidence Database. References were managed using Endnote X8 software version 20.2.1 (Clarivate Analytics). Subject headings and keywords for each database are described in eTable 5 in the Supplement, and full search strategies for each database are defined in the protocol.^14^

### Eligibility Criteria

To be maximally inclusive, studies had to include estimates of association between preoperative or intraoperative factors and physical function at 3, 6, or 12 months after TKA. We considered studies eligible if participants were adults diagnosed with osteoarthritis scheduled for primary TKA. Prospective longitudinal observational studies and randomized clinical trials that provided sufficient estimates of association were eligible. We excluded retrospective and case-control studies, as well as conference abstracts. We also excluded studies with mixed patient populations (eg, rheumatoid arthritis, total hip arthroplasty, or unicompartmental arthroplasty) if separate outcome data were not reported for osteoarthritis and TKA.

### Outcomes

The primary outcome was physical function at 12 months after TKA. Secondary outcomes were physical function 3 and 6 months after TKA.

### Study Selection and Data Extraction

Data from included studies were extracted to a standardized extraction form, with details in the published protocol.^14^ Data included study design, sample size, country, age, sex, body mass index (BMI [calculated as weight in kilograms divided by height in meters squared]), outcome measures used, data collection time points, statistical analyses, and estimates of association. One reviewer performed data extraction (U.O.), while another reviewer checked data accuracy against source material (M.F.L.). Two reviewers (U.O. and M.F.L.) evaluated titles and abstracts for applicability, then read and checked full-text publications against eligibility criteria. Another author (E.D.) was involved in resolving disagreements.

### Methodological Quality

Risk of bias was assessed using the Quality in Prognosis Studies (QUIPS) tool,^17^ following the strategy described in the protocol,^14^ in which 2 reviewers (U.O. and M.F.L.) independently assessed risk of bias and had consensus discussions before arriving at consensus. In cases of disagreement, E.D. was involved in the final decision. QUIPS has 6 risk domains: study participation, attrition, prognostic factor measurement, statistical analysis and reporting, confounding, and outcome measurement.

### Certainty of Evidence

Two researchers (U.O. and M.F.L.) rated certainty of evidence by consensus discussion using the Grading of Recommendations, Assessment, Development and Evaluation (GRADE) framework.^18,19^ In some cases, a third researcher (E.D.) was involved in discussions. Certainty of evidence was graded as high, moderate, low, or very low. We used GRADEpro GDT (McMaster University) to summarize evidence.

### Statistical Analysis

Findings for all included studies were synthesized by outcomes at 3, 6, or 12 months after TKA as described in the protocol.^14^ We were unable to complete planned multivariate random-effects meta-analysis because extracted data were too sparse (with a large number of factors reported by relatively few studies). Accordingly, we used a frequentist version of the bayesian multivariate model.^15^ Additional protocol deviations are explained in eMethods in the Supplement.

To quantify associations between potential factors and the outcome, we extracted odds ratios (ORs), risk ratios (RRs), linear model coefficients (including differences), or correlations using discrete or continuous scales. We meta-analyzed hyperbolic arctangent–transformed correlation coefficients,^20^ which under reasonable assumptions can be imputed for these measures of association and are invariant under linear transformation. This approach allowed inclusion of studies using various measurement tools and analyses in the meta-analysis.

We anticipated that studies would use different instruments and statistical methods that could lead to between-study heterogeneity. Therefore, multivariate random-effects meta-analysis was conducted to estimate mean correlations (ie, not common correlations) between factors and postoperative physical function.

Heterogeneity was quantified using *I*^2^ statistics. We used *P* scores that measured the certainty that the mean correlation for a factor was larger than those for all other factors.^21^ We also performed exploratory univariate meta-analyses and multivariate meta-analyses (after removing factors supported by few studies to reduce the problem of sparsity of estimation). Estimates from 3 models were compared for consistency. Finally, sensitivity analyses on physical function at 12 months after TKA were conducted for each QUIPS domain by excluding studies judged as high risk of bias and rerunning multivariate meta-analysis.

Statistical analyses were performed using Stata statistical software version 16 (StataCorp). We report mean correlations with 95% CIs. We did not prespecify any hypothesis testing but report 2-sided *P* values for completeness.

## Results

**Figure 1.**
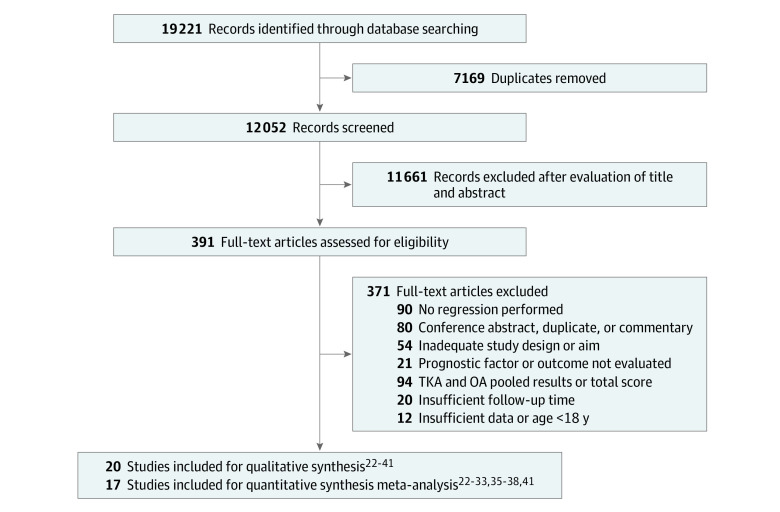
Flowchart of Included Studies OA indicates osteoarthritis; TKA, total knee arthroplasty.

The Figure 1 study flow diagram outlines study selection and reasons for exclusion.^22,23,24,25,26,27,28,29,30,31,32,33,34,35,36,37,38,39,40,41^ From 12 052 articles screened for title and abstracts, 391 articles were selected for full-text examination, with 20 studies^22,23,24,25,26,27,28,29,30,31,32,33,34,35,36,37,38,39,40,41^ (total sample = 11 317 patients) for qualitative analysis at 3, 6, and 12 months and 17 studies^22,23,24,25,26,27,28,29,30,31,32,33,35,36,37,38,41^ for quantitative analysis at 6 and 12 months. Individual study characteristics are detailed in the Table.^22,23,24,25,26,27,28,29,30,31,32,33,34,35,36,37,38,39,40,41^ All were prospective longitudinal observational designs; no randomized trial met inclusion criteria. We identified 37 factors across 20 studies. There were 8 studies^26,27,28,29,30,34,37,38^ conducted in Europe, 6 studies^24,31,32,33,39,40^ in Asia, 4 studies^25,35,36,41^ in North America, and 1 study^22^ in Australia, and 1 study^23^ was multicontinental (ie, Australia, Europe, and North America). Sample sizes ranged from 49 patients^36^ to 5309 patients.^31^ Mean age varied from 63 years^35^ to 75 years,^32^ and representation of women ranged from 49.3%^36^ to 90.0%.^32^ The most common physical function measure was the Western Ontario and McMaster Universities Arthritis Index (WOMAC). We excluded 6 studies from analysis.^42,43,44,45,46,47^ owing to unsuccessful attempts to obtain missing data. Sedentary behavior,^40^ lack of energy,^38^ drowsiness,^38^ sleeping difficulties,^38^ bloating,^38^ worrying,^38^ and problems with sexuality were reported once^38^ and were not included in the meta-analysis.

**Table.** Characteristics of Reviewed Studies

Source	Country	Design	Patients analyzed, No.	Data collection	Follow-up, mo	Baseline age, y	Patients, No./Total No. (%)		Analysis	Factors measured	Outcome measured
							Female	Male			
Berghmans et al,^37^ 2019^a^	Netherlands	PC	146	NA	3	Mean, 66.4	79/150 (53)	71/150 (47)	Stepwise multiple linear regression	Mental health (SF-36), physical function (WOMAC), knee stiffness (WOMAC)	WOMAC
Lindner et al,^34^ 2018	Germany	PC	61	NA	3	Mean, 67	37/61 (61)	24/61 (39)	Stepwise multiple linear regression	Pain (WOMAC)	WOMAC
Lingard et al,^23^ 2007^a^	UK, US, Canada, Australia	PC	676	1997-1999	3	Distress: median, 70	574/676 (85)	102/ 676 (15)	Repeated measures	Psychological distress (SF-36)	WOMAC
						Nondistress: median, 71					
Luo et al,^39^ 2019	China	PC	471	2017-2018	3	Mean, 64.3	357/471 (76)	114/471 (24)	Pearson correlation	Sleep dysfunction (PSQI), daytime sleepiness (ESS), sleep quality (self-developed scale [0-10])	KSS
Bugada et al,^29^ 2017	Italy	PC	563	2012-2015	6	Median, 72	421/606 (69)	185/606 (31)	Logistic regression	Comorbidity (ASA Physical Status Classification System)	NRS
Engel et al,^36^ 2004	US	PC	54	NA	6	Mean, 68	26/74 (49%)	28 /74 (51)	Multiple hierarchical regression	AHI	WOMAC
Escobar et al,^30^ 2007	Spain	PC	640	1999-2000	6	Mean, 72	473/471 (74%)	167/471 (26)	General linear model	Age (y), sex (men/women), social support (yes/no), comorbidity (CCI), physical function (WOMAC), low back pain (yes/no), mental health (SF-36)	WOMAC
Hylkema et al,^35^ 2019	US	PC	131	2012-2014	6	Mean, 61	114/ 183 (62)	69/ 183 (38)	Univariate linear regression	Pain catastrophizing (PCS)	WPAI:SHP
Oka et al,^40^ 2020	Japan	PC	82	2017-2019	6	Mean, 72.1	67/82 (82)	15/82 (18)	Multiple linear regression	Sedentary behavior (MET)	New KSS
Pua et al,^31^ 2019	Singapore	PC	4026	2013-2017	6	Mean, 68	3003/4026 (75)	1026/4026 (25)	Proportional odds ordinal regression	Age (y), sex (men/women), BMI, education (primary, secondary, tertiary), ethnicity (Chinese, Indian, Malay, other), social support (yes/no), comorbidities (yes/no) contralateral knee pain (KSS), pain (OKQ), knee extension and flexion (goniometer), physical function (categories), depression (SF-36)	OKQ
Sugawara et al,^32^ 2017	Japan	PC	59	2011-2012	6	Mean, 75	53/59 (90)	6/59 (10)	Stepwise multiple regression	TSLS	JKOM
Taniguchi et al,^33^ 2016	Japan	PC	81	2013-2014	6	Mean, 72	73/81 (90)	8/81 (10)	Multiple linear regression	TUG	TUG
Yang et al,^41^ 2019	US	PC	107	2010-2011	6	Mean, 65	55/107 (51)	42/107 (49)	Multivariate logistic regression	Mental health (SF-36), pain catastrophizing (PCS), comorbidity (CCI), use device (yes/no)	WOMAC
Berghmans et al,^37^ 2019^a^	Netherlands	PC	144	NA	12	Mean, 66.4	79/150 (53)	71/150 (47)	Stepwise multiple linear regression	Physical function (WOMAC), knee function (KSS)	WOMAC
Dowsey et al,^22^2012	Australia	PC	473	2006-2007	12	Mean, 71	331/478 (69)	142/478 (31)	Multivariate linear regression	Age (y), sex (men/women), BMI, comorbidity (CCI), pain (IKSS), physical function (IKSS), mental health (SF-12), K-L grade, cruciate retaining, patella resurface, multicompartment OA	IKSS
Lindberg et al,^38^ 2020	Norway	PC	182	2012-2014	12	Mean, 67	124/ 182 (68)	58/182 (32)	Multivariate logistic regression	Age (y), sex (men/women), pain (BPI), lack of energy, drowsiness, sleeping difficulties, bloating, worrying, sexuality problems (MSAS-SF)	BPI
Lingard et al,^23^ 2007^a^	UK, US, Canada, Australia	PC	676	1997-1999	12	Distress: median, 70	574/676 (85)	102/ 676 (15)	Logistic regression	Psychological distress (SF-36)	WOMAC
						Nondistress: median, 71					
Nankaku et al,^24^ 2018	Japan	PC	115	2013-2015	12	Mean, 71	99/115 (86)	16/115 (14)	Stepwise multiple regression	Age (y), physical function (KSS), TUG	KSS
Sullivan et al,^25^2011	Canada	PC	120	NA	12	Mean, 67	73/120 (61)	47/120 (39)	Multiple regression	Age (y), sex (men/women), BMI, comorbidity (CCI), physical function and pain (WOMAC), pain catastrophizing (PCS), depression (PHQ-9), kinesiophobia (TSK), surgery duration (min)	WOMAC
Tilbury et al,^26^ 2018	Netherlands	PC	146	2011-2012	12	Mean, 67	101/146 (69)	87/146 (31)	Multivariate linear regression	BMI, mental health (SF-36), physical function (KOOS), outcome expectancies (HSS hip replacement and knee replacement expectations surveys)	KOOS
van de Water et al,^27^ 2019	Netherlands	PC	559	2012-2015	12	Mean, 67	378/559 (68)	181/559 (32)	Multivariate linear regression	Pain (KOOS), K-L grade	KOOS
Wylde et al,^28^ 2012	UK	PC	220	NA	12	Median, 70	136/220 (62)	84/220 (38)	Ordinary least square regression	Age (y), sex (men/women), comorbidity (SCQ), physical function (WOMAC), depression and anxiety (HADS), pain self-efficacy (PSEQ)	WOMAC

Abbreviations: AHI, Arthritis Helplessness Index; ASA, American Society of Anesthesiologists; BMI, body mass index; BPI, Brief Pain Inventory; CCI, Charlson Comorbidity Index; ESS, Epworth Sleepiness Scale; HADS, Hospital Anxiety and Depression Scale; HSS, Hospital for Special Surgery; IKSS, International Knee Society Score; JKOM, Japanese Knee Osteoarthritis Measure; K-L, Kellgren-Lawrence; KOOS, Knee Injury and Osteoarthritis Outcome Score; KSS, Knee Society Clinical Rating System; MET, Metabolic Equivalent of Tasks; MSAS-SF, Memorial Symptom Assessment Scale Short Form; NA, not applicable; NRS, numerical rating scale; OKQ, Oxford Knee Questionnaire; PC, prospective cohort; PCS, Pain Catastrophizing Scale; PHQ-9, Patient Health Questionnaire; PSEQ, Pain Self-Efficacy Questionnaire; PSQI, Pittsburgh Sleep Quality Index; SCQ, Self-Administered Comorbidity Questionnaire; SF-12, 12-Item Short-Form Health Survey 12; SF-36, 36-Item Short Form Health Survey; TSK, Tampa Scale of Kinesiophobia; TSLS, time single legged stand with eyes open; TUG, Timed Up and Go; WPAI:SHP, Work Productivity and Activity Impairment Questionnaire: Specific Health Problem; WOMAC, Western Ontario and McMaster Universities Osteoarthritis Index.

^a^ Study with 2 follow-up times.

**Figure 2.**
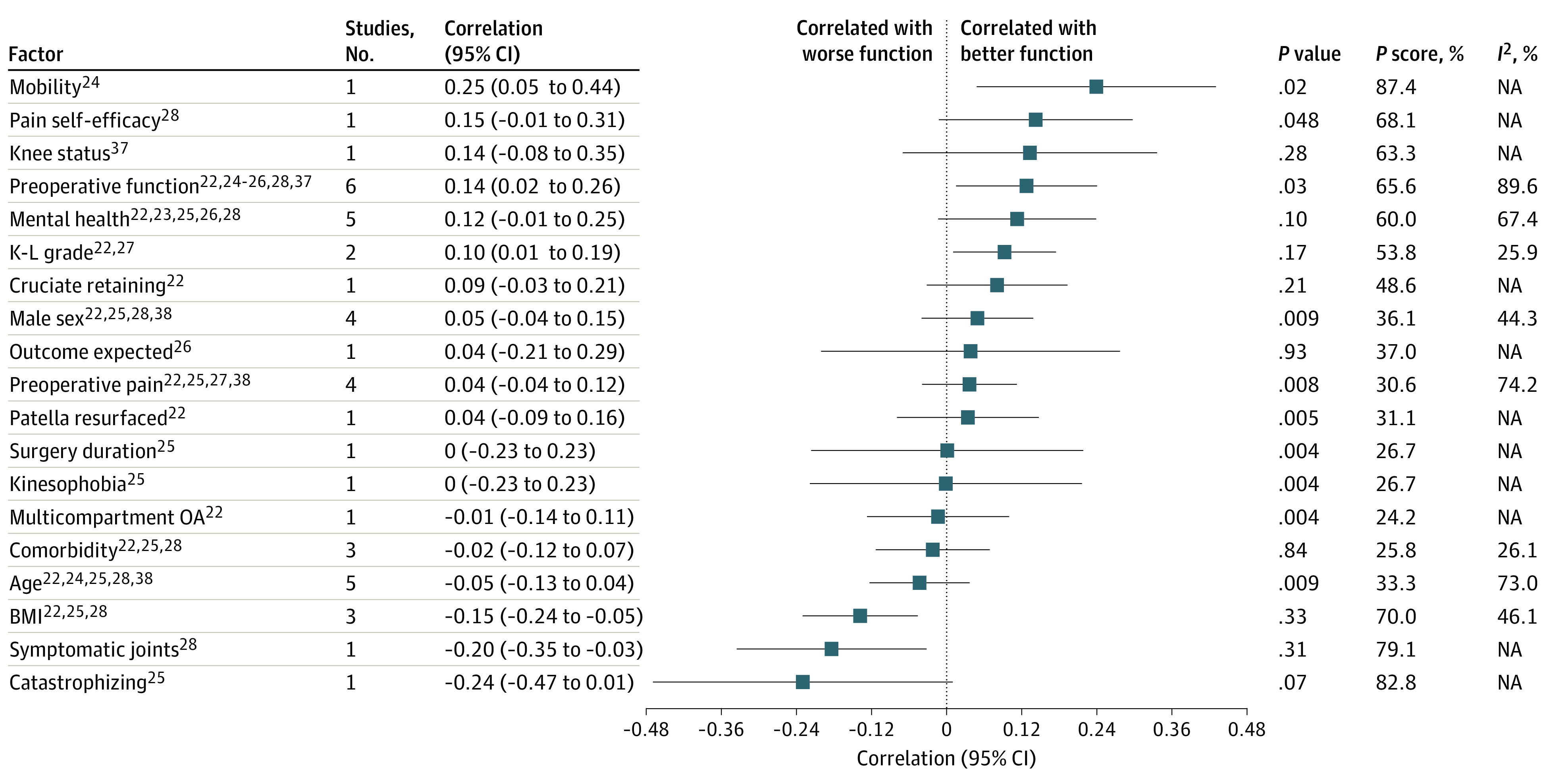
Forest Plot of Factors Associated With Physical Function at 12 mo BMI indicates body mass index; K-L, Kellgren-Lawrence; NA, not applicable; OA, osteoarthritis. Direction of correlation: increased values of factors correlate with better postoperative function for all factors except dichotomous values (ie, cruciate retaining, male sex, patella resurfaced, and multicompartment OA), for which presence of factor correlates with better postoperative function.

**Figure 3.**
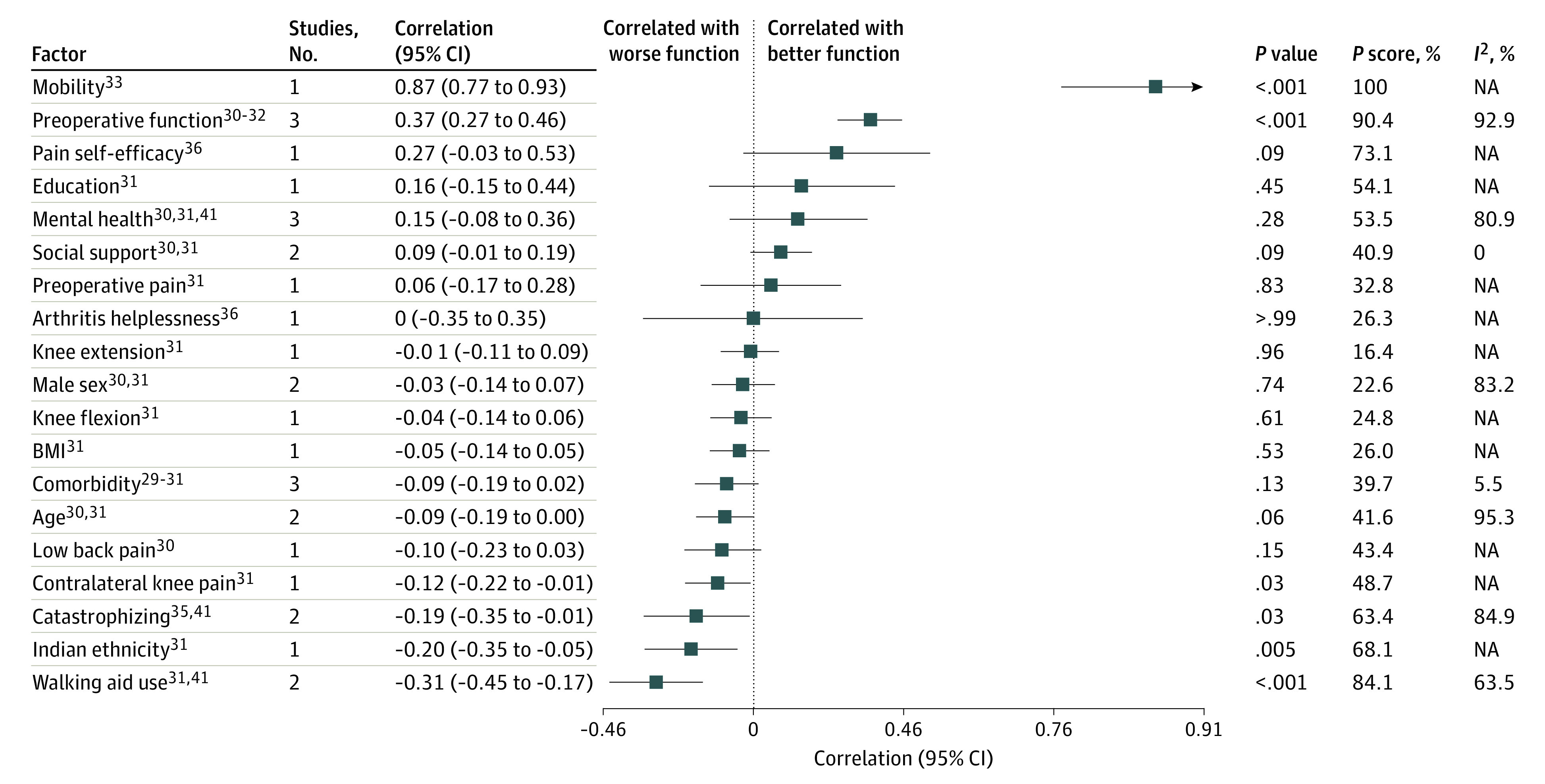
Forest Plot of Factors Associated With Physical Function at 6 mo BMI indicates body mass index; NA, not applicable. Direction of correlation: increased values of factors correlate with better postoperative function for all factors except dichotomous values (ie, male sex, Indian ethnicity, and walking aid use), for which presence of factor correlates with better postoperative function.

Estimates of correlations of factors with function are reported separately for 6-month and 12-month outcomes (Figure 2 and Figure 3). Results from 2 or more studies that could be statistically combined in multivariate meta-analysis are reported subsequently. Explorations of sensitivity analysis are in eFigure 1 and eTable 1 in the Supplement, while explorations of potential inconsistencies and results from exploratory univariate meta-analyses are in eFigures 2 and 3 in the Supplement. Labels for included factors are defined in eTable 3 and reason for exclusion of the individual studies are described in eTable 6 in the Supplement. Positive correlations correspond to better function postoperatively.

**Figure 4.**
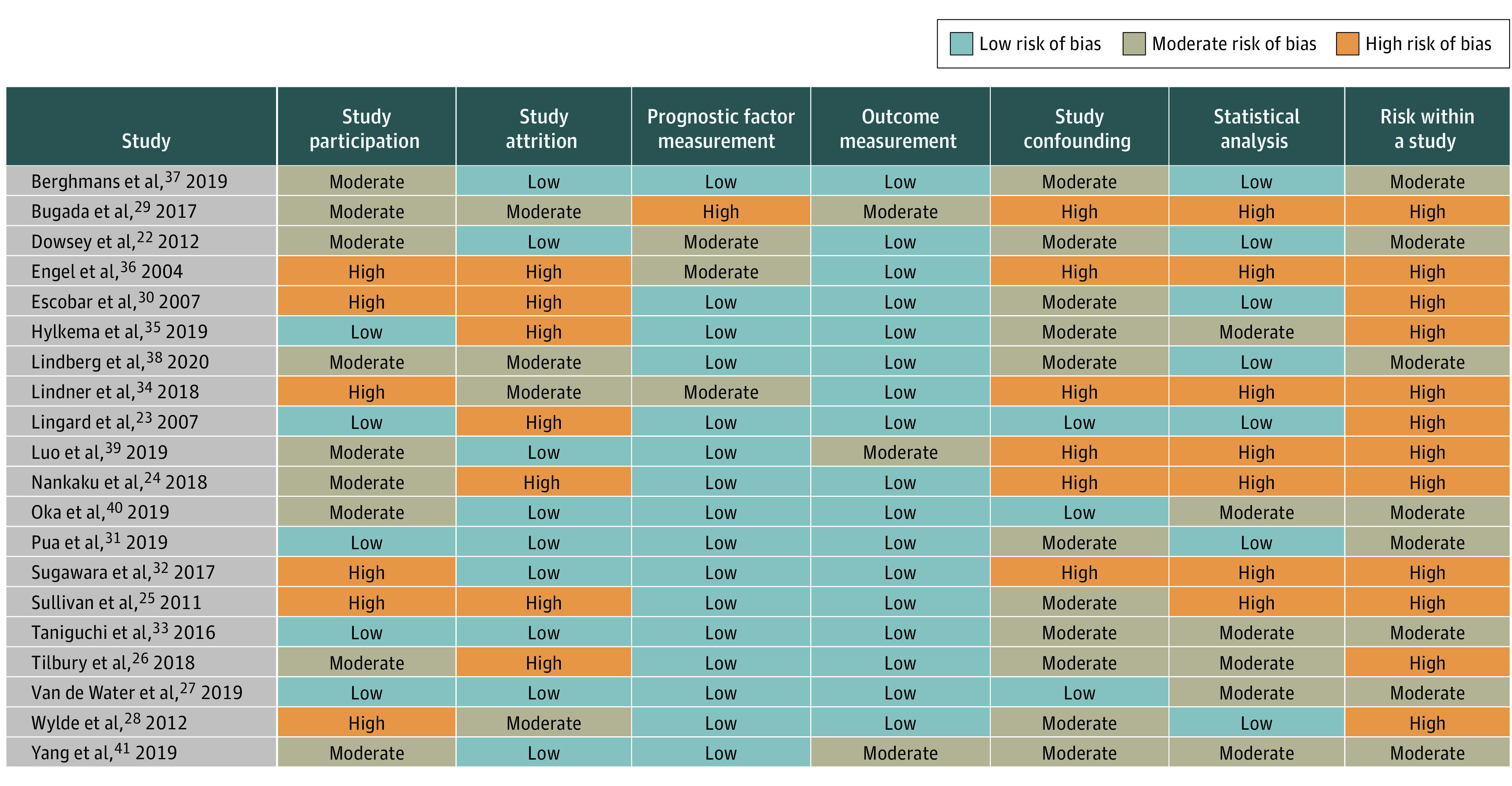
Risk of Bias

There were 9 studies with 2637 patients that reported estimates for 25 potential factors for our primary outcome, physical function at 12 months after TKA.^22,23,24,25,26,27,28,37,38^ Preoperative function (6 studies),^22,24,25,26,28,37^ mental health (including anxiety, depression, and psychological distress [5 studies]),^22,23,25,26,28^ and age (5 studies)^22,24,25,28,38^ were the most frequently reported factors. Several studies were judged as at high risk of bias on 1 or more domains (Figure 4).^23,24,25,26,28,29,30,32,34,35,36,39^ Multivariate meta-analytical correlation coefficient estimates are in Figure 2.^22,23,24,25,26,27,28,37,38^

Mean correlation with higher BMI was estimated to be −0.15 (95% CI −0.24 to −0.05; *P* = .33; *P* score = 70.0%; 3 studies^22,25,26^; moderate-certainty evidence and moderate heterogeneity among reported estimates of association [*I*^2^ = 46%]). Mean correlation with better physical function was estimated to be 0.14 (95% CI, 0.02 to 0.26; *P* = .03; *P* score = 65.6%; 6 studies^22,24,25,26,28,37^; low-certainty evidence and substantial heterogeneity among estimates of association [*I*^2^ = 90%]), while mean correlation with better mental health was estimated to be 0.12 (95% CI, –0.01 to 0.25; *P* = .10; *P* score = 60.0%; 5 studies^22,23,25,26,28^; moderate-certainty evidence and substantial heterogeneity among reported estimates of association [*I*^2^ = 67%]) and mean correlation with more severe osteoarthritis was estimated to be 0.10 (95% CI, 0.01 to 0.19; *P* = .17; *P* score = 53.8%; 2 studies^22,27^; high-certainty evidence and heterogeneity between reported estimates [*I*^2^ = 26%]). High-certainty evidence and heterogeneity for osteoarthritis may not be important. We were unable to conclude that clinically meaningful correlations did not exist for the other 15 factors owing to limited evidence (ie, wide CIs).

In the prespecified sensitivity analysis (eTable 1 in the Supplement), mean correlation with better physical function was estimated to be 0.20 (95% CI, 0.04 to 0.36; *P* = .02 vs coefficient = 0.14; 95% CI, 0.02 to 0.26 when including all studies). Mean correlation with BMI was estimated to be –0.17; 95% CI, –0.28 to –0.06; *P* < .001 vs coefficient = –0.15; 95% CI, –0.24 to –0.05 when including all studies), while mean correlation with mental health was estimated to be 0.13 (95% CI, –0.04 to 0.29; *P* = .02 vs coefficient = 0.12; 95% CI, –0.01 to 0.25 when including all studies), and mean correlation with osteoarthritis severity was estimated to be 0.10 (95% CI, –0.01 to 0.20; *P* = .05 vs coefficient = 0.10; 95% CI, 0.01 to 0.19 when including all studies).

For the secondary outcome, physical function 6 months after TKA, 9 studies with 5743 participants reported estimates on 20 potential factors.^29,30,31,32,33,35,36,40,41^ Estimated correlation coefficients from multivariate meta-analysis are in Figure 3.^29,30,31,32,33,35,36,41^ Mean correlation with more catastrophizing was estimated to be –0.19 (95% CI, –0.35 to –0.01; *P* = .03; *P* score = 63.4%; 2 studies^35,41^; very low–certainty evidence and substantial heterogeneity between reported estimates of association [*I*^2^ = 85%]), while mean correlation with walking use was estimated to be –0.31 (95% CI, –0.45 to –0.17; *P* < .001, *P* score = 84.1%; 2 studies^31,41^; high-certainty evidence and substantial heterogeneity between reported estimates of association [*I*^2^ = 63%]). Mean correlation with better physical function was estimated to be 0.37 (95% CI, 0.27 to 0.46; *P* < .001; *P* score = 90.4; 3 studies^30,31,32^; moderate-certainty evidence and substantial heterogeneity among reported estimates of association [*I*^2^ = 93%]), while mean correlation with better mental health was estimated to be 0.15 (95% CI, –0.08 to 0.36; *P* = .28; *P* score = 53.5; 3 studies^30,31,41^; high-certainty evidence and substantial heterogeneity among reported estimates of association [*I*^2^ = 81%]). We were unable to conclude that clinically meaningful correlations did not exist for the other 15 factors owing to limited evidence (ie, wide CIs). For the 3-month outcome, we were unable to perform multivariate meta-analysis, as shown in eTable 2 in the Supplement.

QUIPS domains most frequently assessed as at low risk of bias were prognostic factor measurement (16 studies^23,24,25,26,27,28,30,31,32,33,37,38,39,40,41^) and outcome measurement (17 studies^22,23,24,25,26,27,28,30,31,32,33,34,35,36,37,38,40^). For high risk of bias, QUIPS domains most often assessed were attrition (7 studies^23,24,25,26,30,35,37^) and statistical analysis (7 studies^24,25,29,32,34,36,39^), as shown in Figure 4.

Our GRADE certainty of evidence judgements are included in previously listed data and in eTable 4 in the Supplement. The most common reasons for downgrading certainty of evidence were risk of bias and imprecision.

## Discussion

To our knowledge, this study is the first prespecified systematic review and meta-analysis using wide eligibility criteria and evaluating certainty of evidence to identify preoperative and intraoperative factors correlated with physical function at 12 months after TKA. Evidence from 7 observational studies^22,24,25,26,27,28,37^ suggested that higher BMI was correlated with poorer physical function 12 months after TKA and that better preoperative physical function and more severe osteoarthritis were correlated with better physical function 12 months after TKA. Importantly, our findings did not suggest that individual patients with a poor risk factor profile will not experience functional improvement if they undergo TKA. Our findings merely suggest that identified factors were correlated with poorer or better physical function in an absolute sense and may therefore be useful for guiding expectations about TKA outcomes.

We found moderate-certainty evidence for a correlation between higher preoperative BMI and worse function at 12 months, with equal correlation in the sensitivity analysis, in which studies judged to be at high risk of bias were removed. This finding is similar to that of another meta-analysis,^13^ in which participants without obesity reported lower rates of disability than participants with obesity. The evidence was not graded, however, and the study included retrospective studies with follow-up at 6 months to 10 years. Although we found a correlation between obesity and poorer physical function after TKA, patients with obesity still experience improved function from baseline^48^ and should thus be considered for surgery. However, the surgeon needs to consider the functional benefit against the risk for complications (eg, septic revisions are more prevalent in patients with severe obesity and super obesity^49^) for each patient and discuss these issues with the patient to encourage realistic expectations before proceeding with TKA.^49^

We found a correlation between better preoperative and better postoperative function at 12 months (low-certainty evidence) and 6 months (moderate-certainty evidence). The correlation remained, with increased coefficients, in the sensitivity analysis. It is not surprising that patients who were healthier before surgery may also have been healthier after surgery. However, our results conflict with those of a systematic review^8^ concluding that lower preoperative function was associated with better function 12 months after TKA. To resolve these conflicting findings, evidence is needed from well-conducted studies using standardized methods to measure factors and outcomes. We also estimated a correlation between more severe osteoarthritis (Kellgren-Lawrence grade) and better physical function at 12 months (high-certainty evidence) in multivariate meta-analysis and sensitivity analysis. These findings are consistent with those of a systematic review^8^ that included retrospective studies with follow-up extending to 1 year. Uncertainty remains regarding evidence for osteoarthritis severity as a factor associated with the outcome at 12 months.^50,51^

Major strengths of our study include following the recently revised Cochrane Handbook^16^ and guidelines for peer-reviewed protocols,^14^ including longitudinal prospective studies reporting associations at predefined times after TKA, and using multivariate meta-analysis when the number of factors was large compared with the number of studies.^15^ Several previous systematic reviews were unable to perform meta-analysis owing to heterogeneity associated with measurement issues, and others used vote counting, a method discouraged in current guidelines.^16^ We used recommended tools to assess risk of bias (QUIPS) and certainty of evidence (GRADE). Additionally, we prioritized transparency with the systematic use of prespecified methods documented in the protocol,^14^ preprint,^15^ and this article’s supplemental materials.

### Limitations

This study has several limitations. To obtain trustworthy estimates without prejudging which factors may have been associated with the outcome, we included a wide range of factors but only from prospective studies reporting associations at specific postoperative times. This necessarily included estimates from studies measuring factors using a range of methods, and so we accounted for heterogeneity in our random-effects meta-analyses. Less heterogeneity was observed across studies using a common measure, particularly 9 studies that used WOMAC to measure physical function. Narrower inclusion criteria could increase the potential for excluding important evidence.^16^ Some studies had large sample sizes and therefore provided precise estimates (ie, narrow CIs). *I^2^* may be misleading when study estimates are very precise because it is statistically easier to distinguish (ie, detect heterogeneity) between study estimates. In this situation, it is important to consider the degree to which study estimates vary from one another and whether this is clinically important, rather than relying solely on *I^2^*. In particular, *I^2^* from prognostic studies may be misleading so *I^2^* statistics should be interpreted cautiously.^18^ Because studies with high risk of bias can lead to biased main results and heterogeneity, we performed prespecified sensitivity analyses and excluded studies assessed as high risk for each QUIPS domain.^14^ We planned to perform analyses of nonreporting bias, small study effects, and subgroup analyses,^14^ but the number of included studies did not meet our prespecified threshold.

We also downgraded certainty of evidence if we judged studies to be at risk of bias. Several studies^11,52,53,54^ had insufficient reporting of important QUIPS domains (such as attrition and statistical analysis), thus lowering the certainty that study estimates were unbiased. We suggest that researchers use tools like QUIPS at the study design stage to encourage low risk of bias in their findings regarding prognostic factors. This review identified some key areas for future research. Uncertainty remains regarding which patients may benefit most from TKA. Because patient preoperative status (ie, BMI, physical function, and osteoarthritis severity) may be correlated with overall outcomes, evidence from high-quality studies is fundamental for developing a prediction model to better identify patients at increased risk of poor outcomes after TKA. Prehabilitation interventions to improve modifiable factors (eg, better mental health) are not well-established.^55,56^ We could not synthesize data for a number of factors given that they were studied only once. For these and other factors and outcomes, such as associations between physical function during the first year after TKA and biomechanical aspects of surgery (eg, implant) or pain management, evidence is lacking, highlighting the need for research from these perspectives with appropriate design and power. Additionally, our study provided evidence at the population level not at the level of individual patients. Our results are important for investigating factors to include in predictive models but should be used with caution at the individual level.

## Conclusions

This study found that there is evidence (with moderate certainty) that higher BMI was correlated with worse physical function and that better physical function (low-certainty evidence) and more severe osteoarthritis (high-certainty evidence) were correlated with better physical function 12 months after TKA. Our findings suggest that these factors should be included in development of predictive models aimed at identifying patients at increased risk of poor function after TKA.

## Notes

Supplement. **eMethods.** Multivariate Meta-analysis **eFigure 1.** Sensitivity Analysis **eFigure 2.** Exploring Potential Inconsistency at 6 and 12 mo **eFigure 3.** Univariate Meta-analysis **eTable 1.** Sensitivity Analysis **eTable 2.** Reported Associations at 3 mo After TKA **eTable 3.** Definition and Labels of Factors **eTable 4.** Grading of Recommendation Assessment, Development and Evaluation **eTable 5.** Search Strategy **eTable 6.** Reason for Exclusion of Individual Studies
