# Supplementary material for: Factors Correlated With Physical Function 1 Year After Total Knee Arthroplasty in Patients With Knee Osteoarthritis: A Systematic Review and Meta-analysis
Source: JAMA Netw Open. 2022 Jul 11;5(7):e2219636. doi: 10.1001/jamanetworkopen.2022.19636 (PMC9274324; doi:10.1001/jamanetworkopen.2022.19636)
Supplement: Supplement. — eMethods. Multivariate Meta-analysis eFigure 1. Sensitivity Analysis eFigure 2. Exploring Potential Inconsistency at 6 and 12 mo eFigure 3. Univariate Meta-analysis eTable 1. Sensitivity Analysis eTable 2. Reported Associations at 3 mo After TKA eTable 3. Definition and Labels of Factors eTable 4. Grading of Recommendation Assessment, Development and Evaluation eTable 5. Search Strategy eTable 6. Reason for Exclusion of Individual Studies [file jamanetwopen-e2219636-s001.pdf]

Supplemental Online Content

Olsen U, Lindberg MF, Rose C, et al. Factors correlated with physical function 1 year after total knee arthroplasty in patients with knee osteoarthritis: a systematic review and meta-analysis. *JAMA Netw Open*. 2022;5(6):e2219636. doi:10.1001/jamanetworkopen.2022.19636

eMethods. Multivariate Meta-analysis
eFigure 1. Sensitivity Analysis
eFigure 2. Exploring Potential Inconsistency at 6 and 12 mo
eFigure 3. Univariate Meta-analysis
eTable 1. Sensitivity Analysis
eTable 2. Reported Associations at 3 mo After TKA
eTable 3. Definition and Labels of Factors
eTable 4. Grading of Recommendation Assessment, Development and Evaluation
eTable 5. Search Strategy
eTable 6. Reason for Exclusion of Individual Studies

This supplemental material has been provided by the authors to give readers additional information about their work.

© 2022 Olsen U et al. *JAMA Network Open.*

eMethods. Multivariate Meta-analysis

Except where noted, we performed statistical analyses according to the method prespecified in our protocol (Olsen 2020).

We imputed correlation coefficients from estimates of association expressed as odds ratios, risk ratios, and linear model coefficients (including differences) as described in our protocol's supplementary materials. Where it was necessary to impute odds ratios from risk ratios prior to imputing correlation, we assumed a prespecified baseline probability of reduced postsurgical function of 20%. We defined canonical directions for all outcomes and factors and inverted reported directions of association as appropriate to ensure consistent directions of association in meta-analysis.

If studies did not report confidence intervals or sampling variances, we imputed them as appropriate (Higgins 2019). If a study did not report exact statements of uncertainty but provided statements about “statistical significance”, we used a conservative approach in which we imputed “worst case” standard errors. For example, we imputed P≤0.01 to mean P=0.01 and “not statistically significant” to mean P=0.99. We performed all meta-analyses on the scale of Fisher’s z (hyperbolic arctangent, not Z-score; Borenstein 2009). We used the inverse transform (hyperbolic tangent) to report meta-analytical estimates as correlation coefficients.

We anticipated that factors may be correlated and that there may be important differences in the methods used to quantify associations. We therefore planned to perform multivariate random-effects meta-analysis for each outcome using White's (2009, 2011) multivariate extension to Riley's (2008) bivariate random-effects model, as implemented in the MVMETA add-on command for Stata. Unfortunately, it was not possible to fit this model given the sparsity of our data. We therefore used a frequentist version of the Bayesian multivariate model we developed for a meta-analysis of pain after total knee arthroplasty (Rose 2020). We had planned to identify factors likely to be most strongly associated with postoperative function by estimating the probability of superiority of each factor using the pbest option of MVMETA. Because that model could not be used, we assessed using P-scores (cf. p-values; Rücker and Schwarzer 2015), in which larger magnitudes were defined to be superior to those with smaller magnitudes. Unlike the probabilities we had planned to estimate, P-scores are not as heavily influenced by imprecisely estimated factors with small point estimates whose confidence intervals extend far beyond those of more precisely estimated factors with larger point estimates. This is particularly important for multivariate meta-analysis of correlations, in which the superiority of a factor is a function of the magnitude of its coefficient rather than its magnitude and direction, as is the case in multiple treatment comparison via network meta-analysis. P-scores are therefore likely to better identify good factors. Multivariate estimates of correlation are presented as forest plots, which

© 2022 Olsen U et al. *JAMA Network Open.*

also show I^2 statistics (the percentage of heterogeneity attributable to between-study differences rather than sampling error) and the numbers of studies that provided usable estimates for each factor. We also performed exploratory univariate meta-analyses for each factor and outcome, but which do not account for correlation. We compared estimates from the three approaches to identify possible inconsistency. We report 95% confidence intervals throughout. Statistical analyses were performed using Stata 16 (StataCorp LLC, College Station, Texas, USA).

We had planned to investigate non-reporting bias and small study effects for factor supported by at least 10 results. However, none of the factors met this criterion. Similarly, we had planned to perform subgroup analyses with respect to study design, type of outcome measurement, and intervention if at least five studies could be included in each subgroup. However, this criterion was not satisfied, and no subgroup analyses were performed.

We performed a sensitivity analysis for the primary outcome (function 12 months post-surgery). For each of the six QUIPS risk of bias domains, we excluded studies judged to be at high risk of bias, re-ran the multivariate meta-analysis, and compared the estimated correlations with those obtained when all studies are included. We had planned to do a leave-one-study-out sensitivity analysis to explore the influence of each study on the meta-analysis results. Unfortunately, this was not feasible. However, the effect of particular studies can be inferred by inspecting the univariate meta-analyses.

Fig. 1. Postsurgical function at 6 and 12 months

The following plot shows the multivariate meta-analytical estimates of correlation at each postoperative follow-up time. Estimates for factor studied at only one postoperative time point are omitted.

© 2022 Olsen U et al. *JAMA Network Open.*

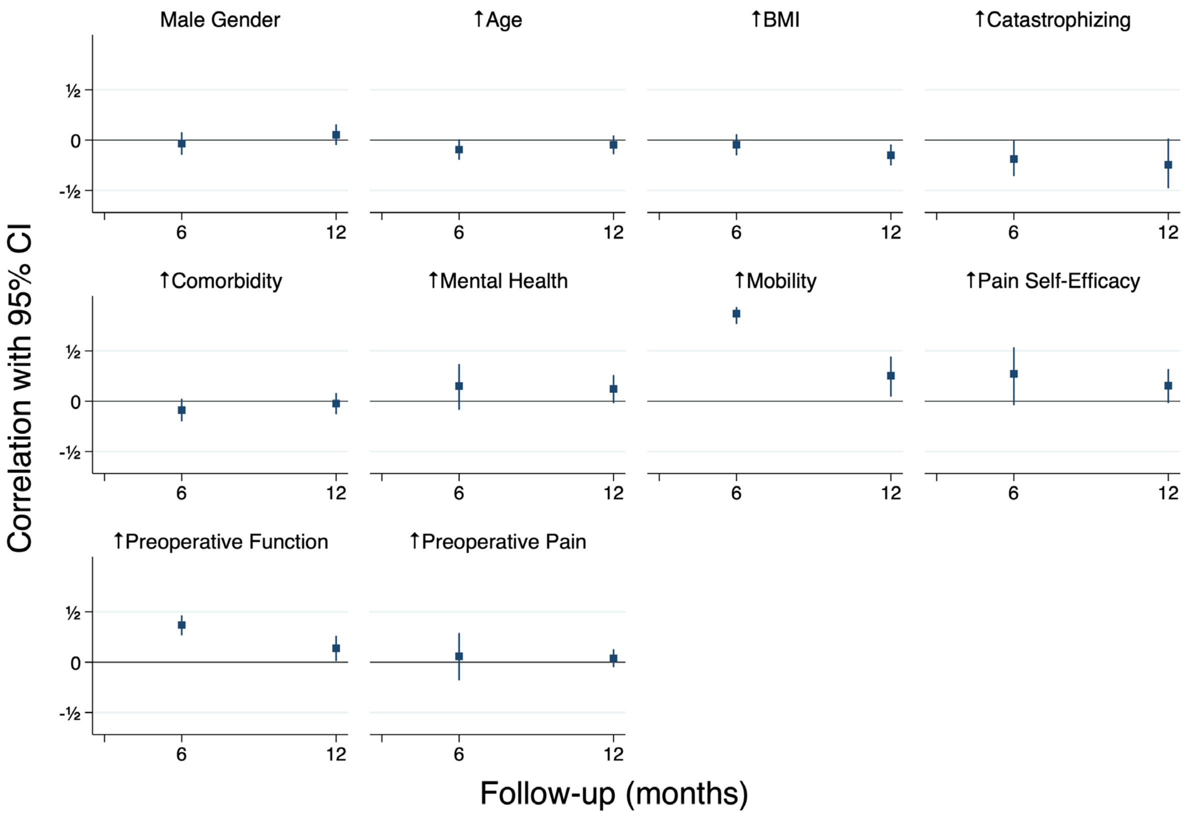

Predictors studied at only one postoperative time point are omitted

© 2022 Olsen U et al. *JAMA Network Open.*

eFigure 1. Sensitivity Analysis

The following plot shows the results of a sensitivity analysis in which the multivariate meta-analysis model was used to estimate correlations for each factor, omitting all estimates from studies judged to be at high risk of bias for each of the six QUIPS domains. Estimates from the full meta-analysis are also included for comparison (shown as "All Studies").

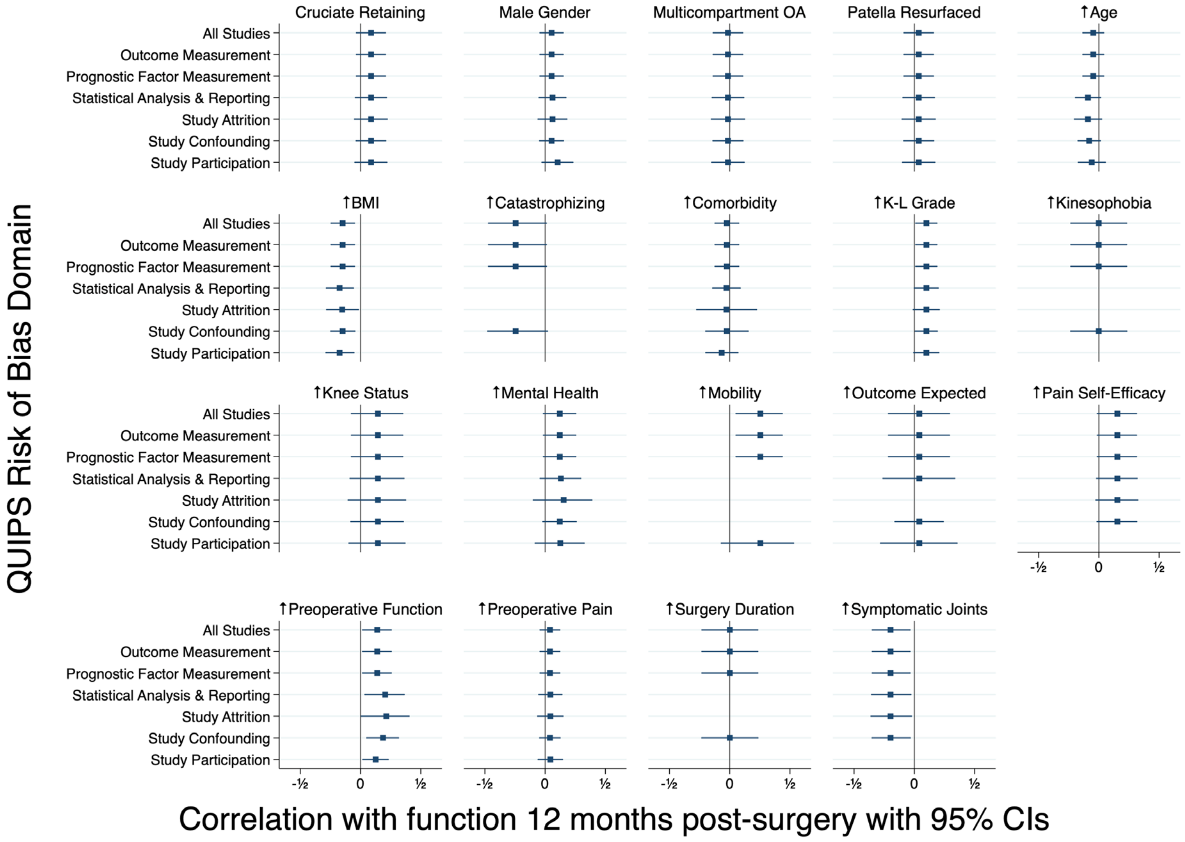

References

Borenstein M, Hedges LV, Higgins JP, et al. Introduction to meta-analysis: John Wiley & Sons; 2009.

Carpenter B, Gelman A, Hoffman MD, Lee D, Goodrich B, Betancourt M et al. Stan: A probabilistic programming language. Journal of Statistical Software 2017, 76(1).

Higgins JPT, Li T, Deeks JJ (editors). Chapter 6: Choosing effect measures and computing estimates of effect. In: Higgins JPT, Thomas J, Chandler J, Cumpston M, Li

T, Page MJ, Welch VA (editors). Cochrane Handbook for Systematic Reviews of Interventions version 6.0 (updated July 2019). Cochrane, 2019.

Lin L and Chu H. Bayesian multivariate meta-analysis of multiple factors. Research Synthesis Methods 2018, 9(2): 261-272.

Olsen U, Lindberg MF, Denison EML, Rose CJ, Gay CL, Aamodt A, Brox IB, Skare Ø, Furnes O, Lee KA, Lerdal A. Predictors of chronic pain and level of physical function in total knee arthroplasty: a protocol for a systematic review and meta-analysis. BMJ Open 2020, 10(9): e037674.

Riley RD, Thompson JR, Abrams KR. An alternative model for bivariate random-effects meta-analysis when the within-study correlations are unknown. Biostatistics 2008, 9: 172-186

Rose CJ, Olsen U, Lindberg MF, Denison EL, Aamodt A, Lerdal A. A new multivariate meta-analysis model for many variates and few studies. arXiv preprint 2020, arXiv:2009.11808. Available at https://arxiv.org/abs/2009.11808

Rücker G, Schwarzer, G. Ranking treatments in frequentist network meta-analysis works without resampling methods. BMC Medical Research Methodology 2015, 15(58).

White IR. Multivariate random-effects meta-analysis. Stata Journal 2009, 9: 40-56.

White IR. Multivariate random-effects meta-regression: Updates to mvmeta. Stata Journal 2011, 11: 255-270.

© 2022 Olsen U et al. *JAMA Network Open.*

eFigure 2. Exploring Potential Inconsistency at 6 and 12 mo

The following forest plots compare estimates between all models (where possible).

Fig. 3. Postsurgical Function (6 months) — Model comparison

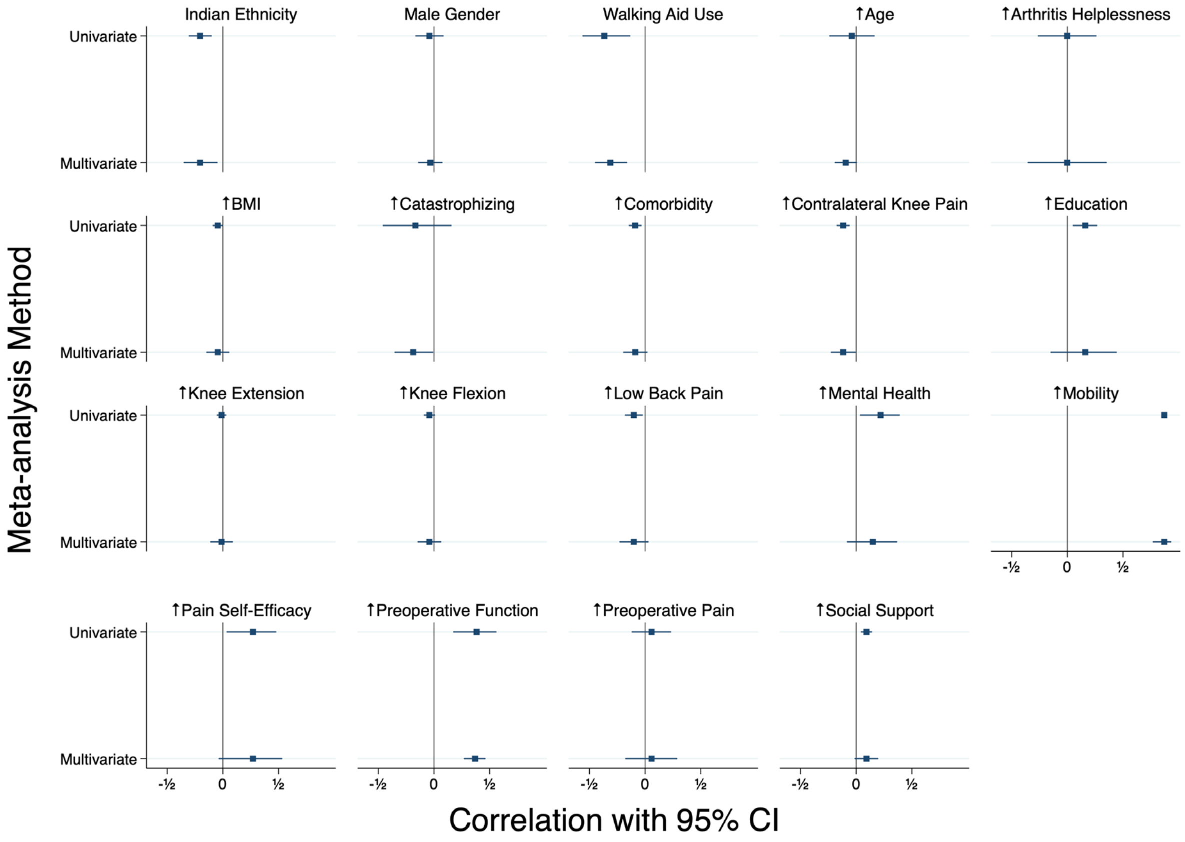

© 2022 Olsen U et al. *JAMA Network Open.*

Fig. 4. Postsurgical Function (12 months) — Model comparison

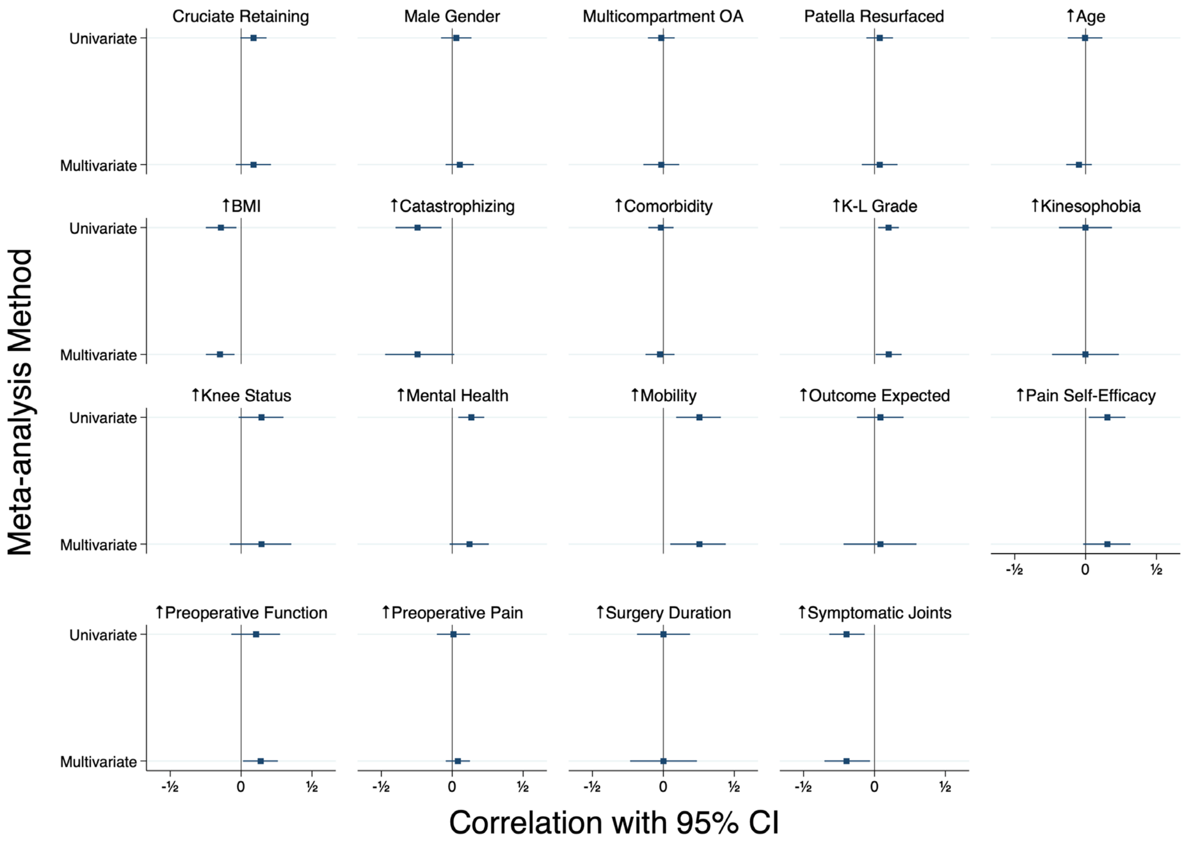

© 2022 Olsen U et al. *JAMA Network Open.*

eFigure 3. Univariate Meta-analysis

The following forest plots show the results of exploratory univariate meta-analyses of the association between individual factor and the outcomes. Note that these results do not account for any correlation between the prognostic factor.

© 2022 Olsen U et al. *JAMA Network Open.*

Function (6 months)

Fig. 5. ↑Arthritis Helplessness

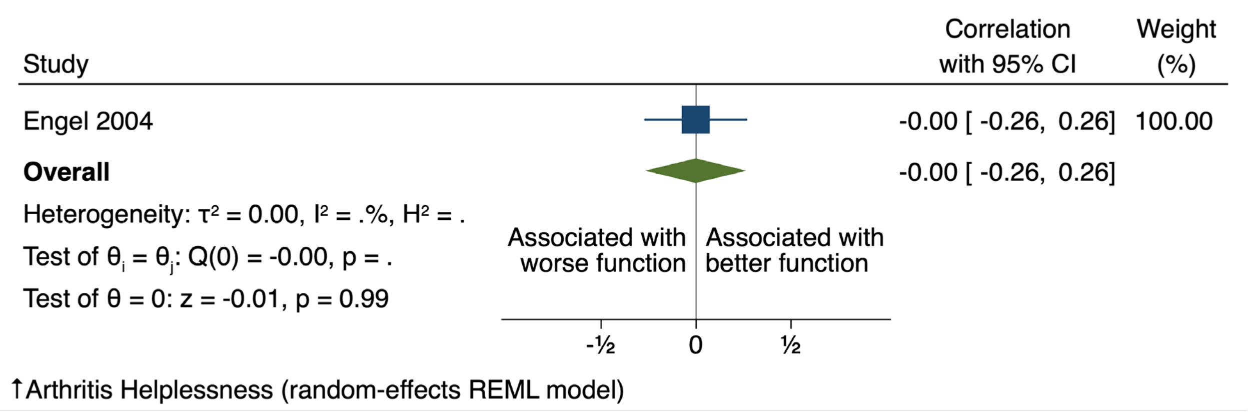

Fig. 6. ↑Education

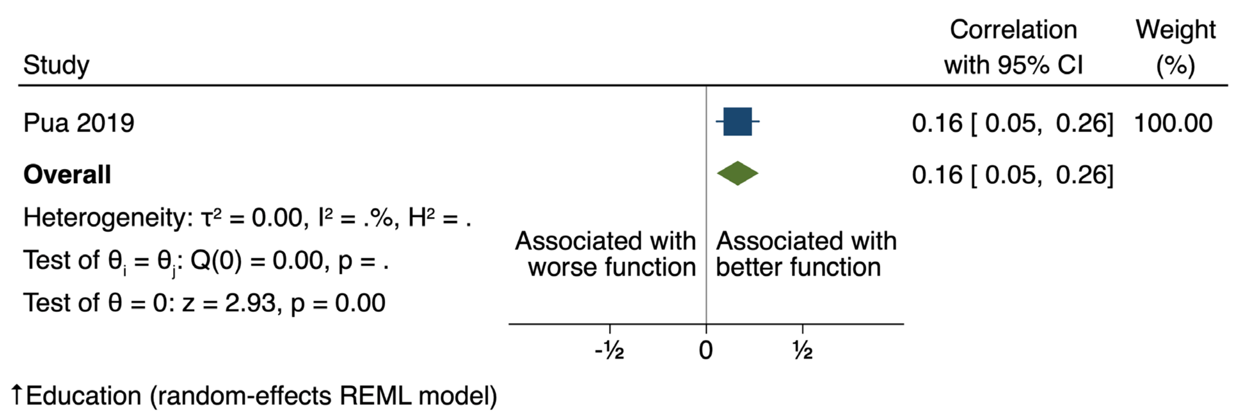

Fig. 7. ↑Mental Health

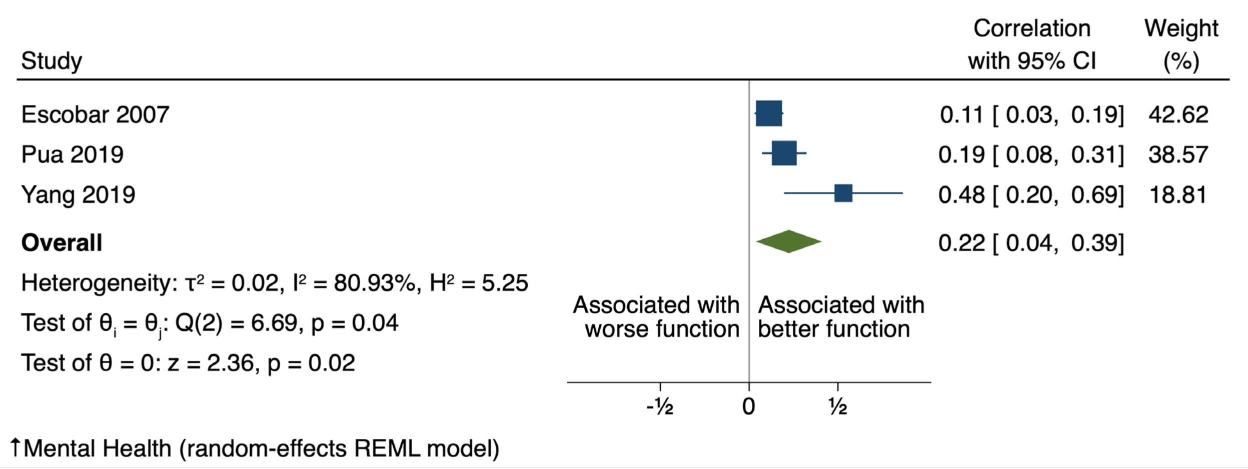

© 2022 Olsen U et al. *JAMA Network Open.*

Fig. 8. ↑Mobility

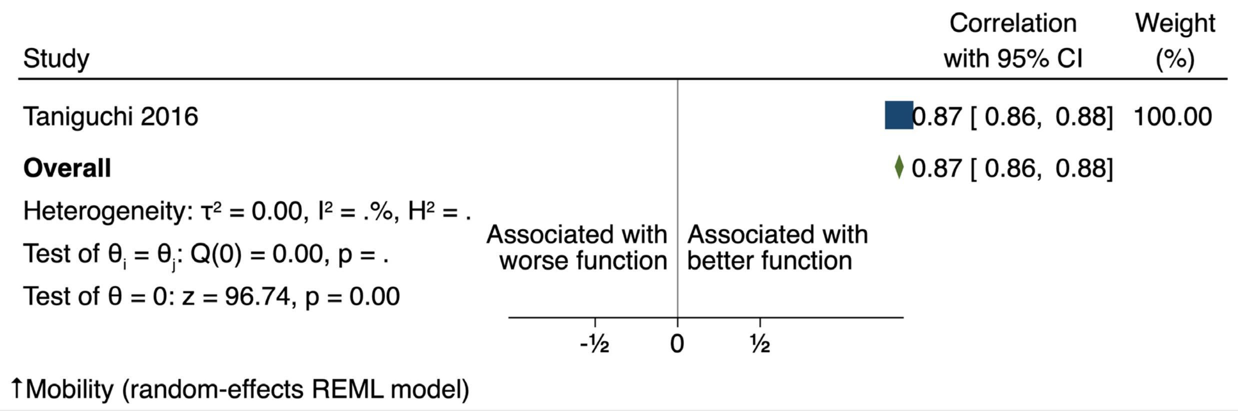

Fig. 9. ↑Preoperative Function

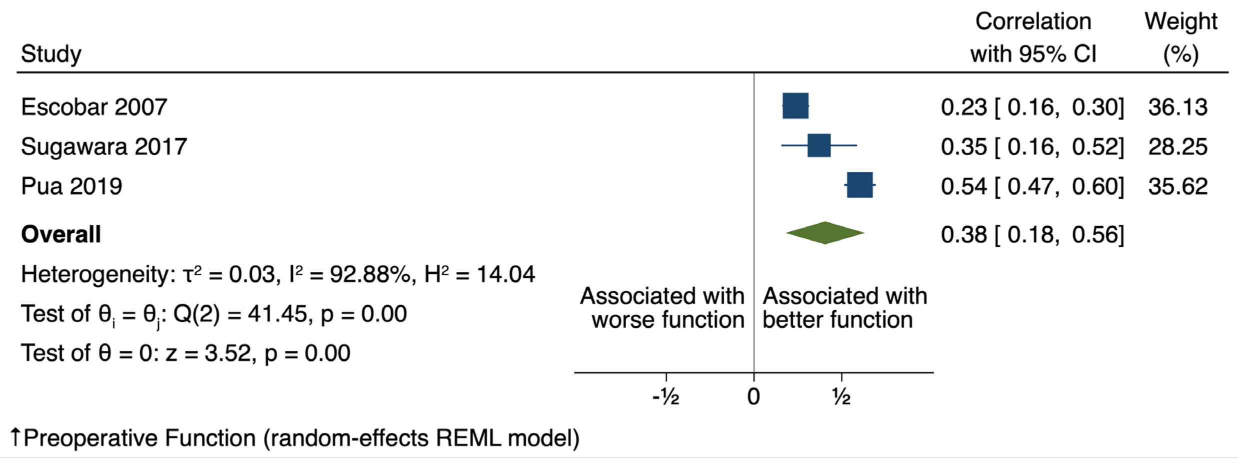

Fig. 10. ↑Catastrophizing

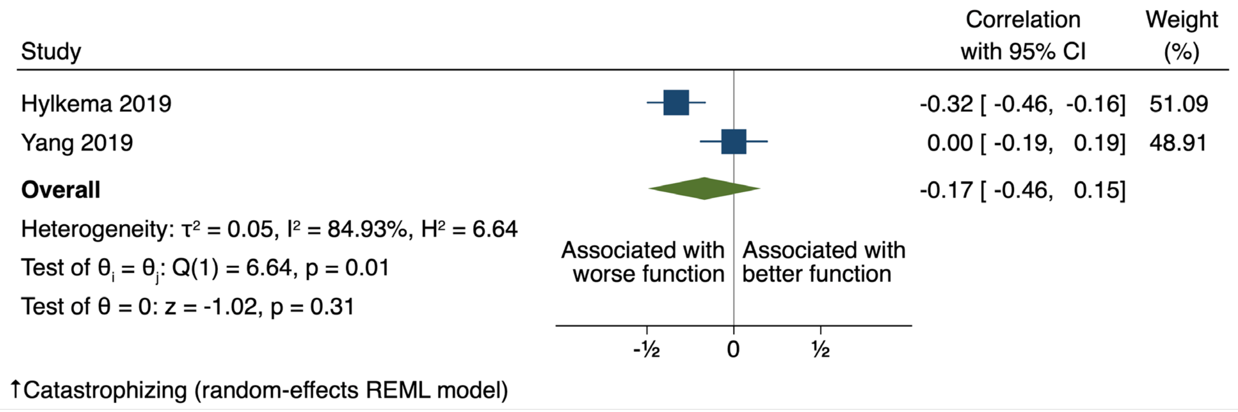

© 2022 Olsen U et al. *JAMA Network Open.*

Fig. 11. ↑Comorbidity

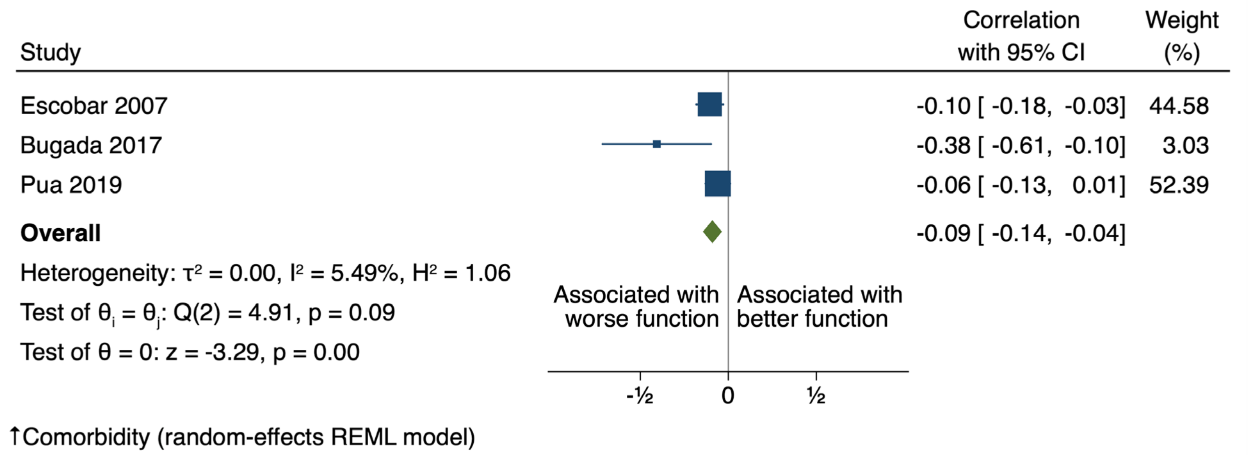

Fig. 12. ↑Contralateral Knee Pain

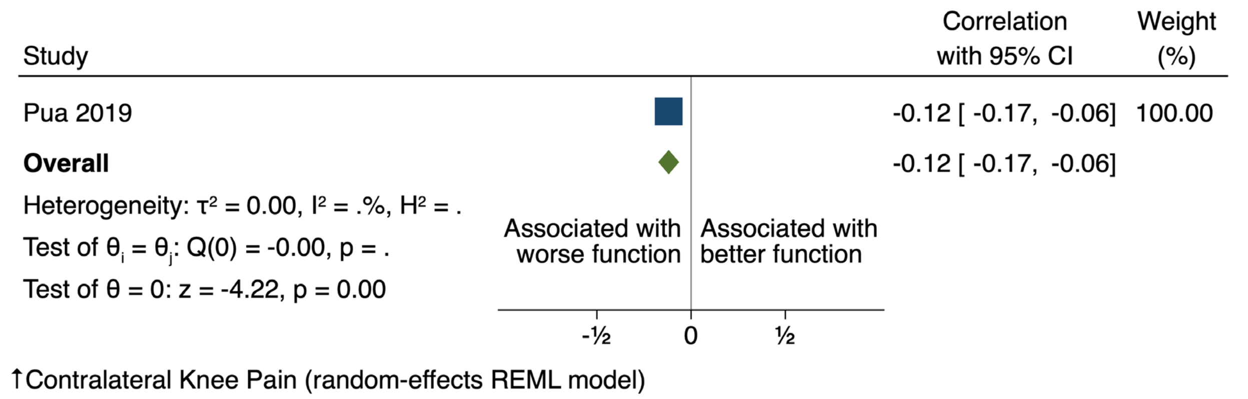

Fig. 13. ↑BMI

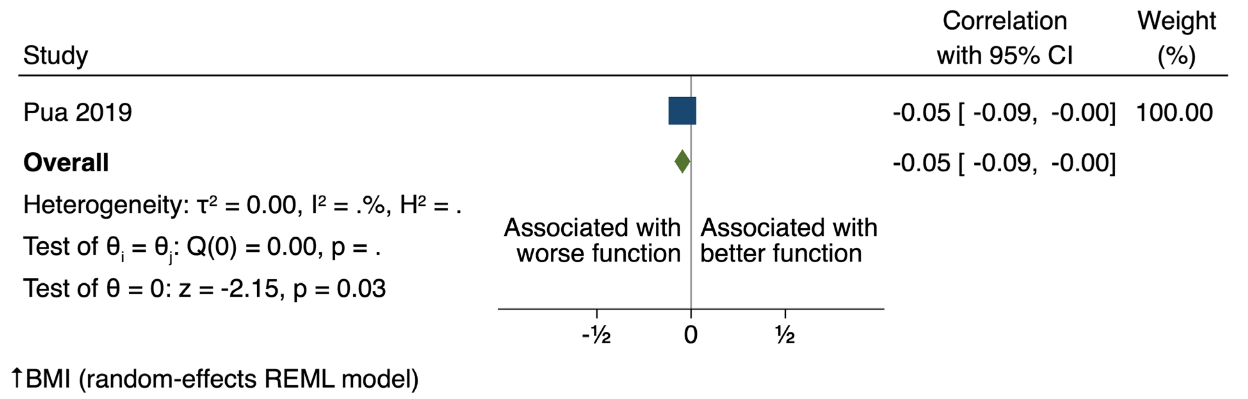

© 2022 Olsen U et al. *JAMA Network Open.*

Fig. 14. Indian Ethnicity

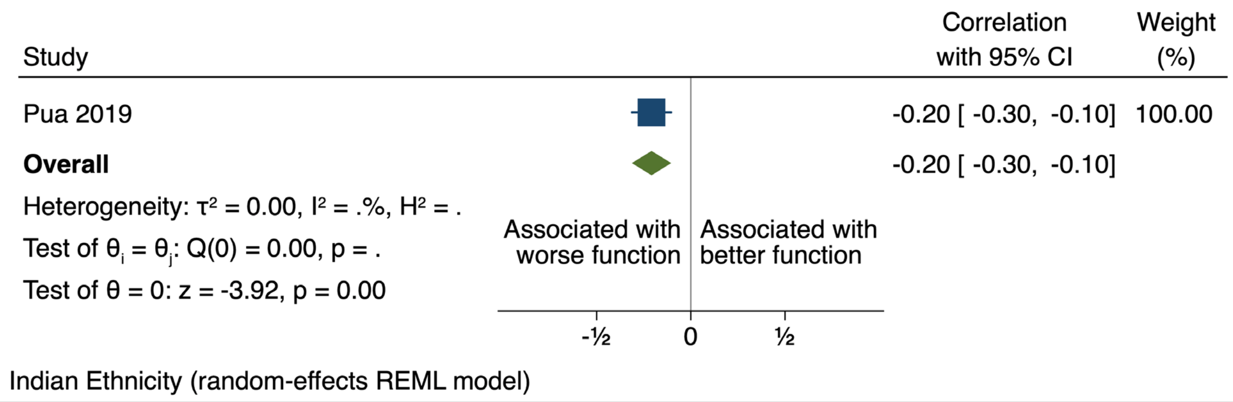

Fig. 15. ↑Low Back Pain

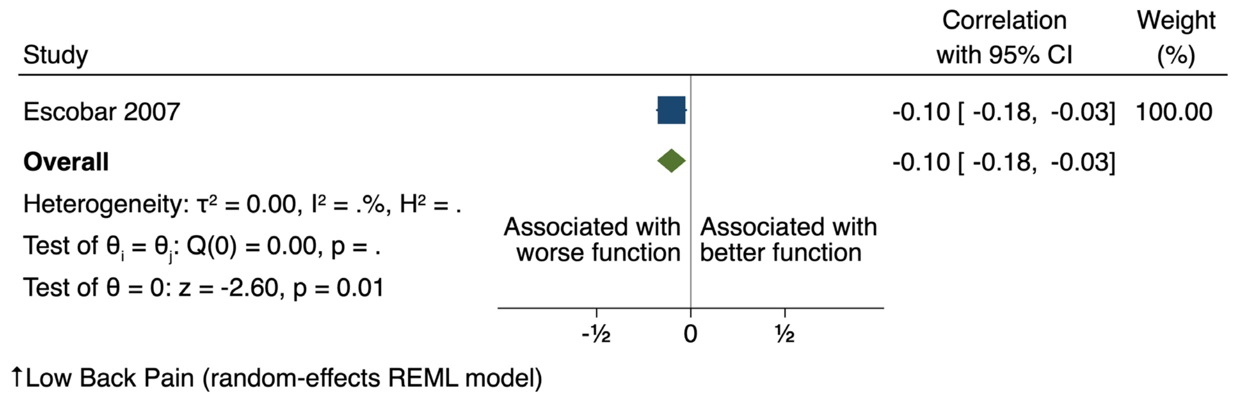

Fig. 16. Male Gender

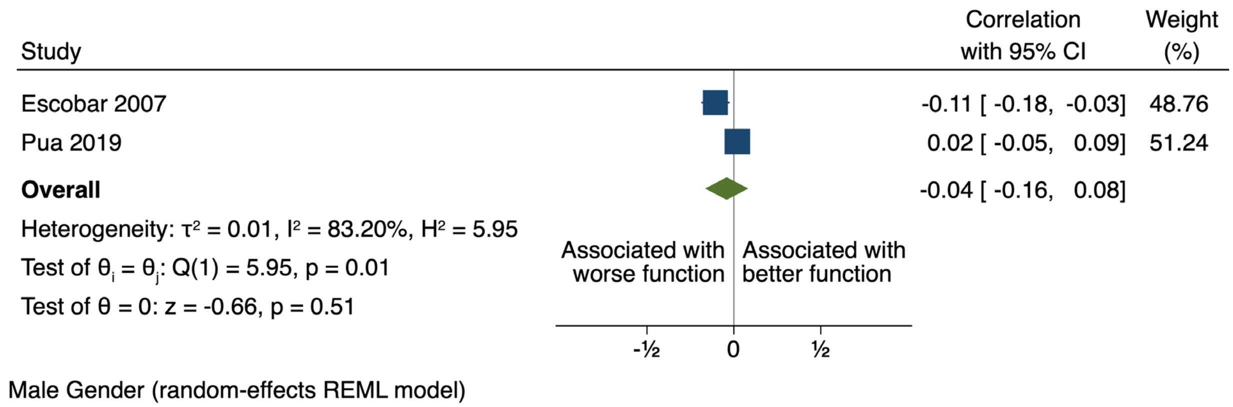

© 2022 Olsen U et al. *JAMA Network Open.*

Fig. 17. ↑Knee Extension

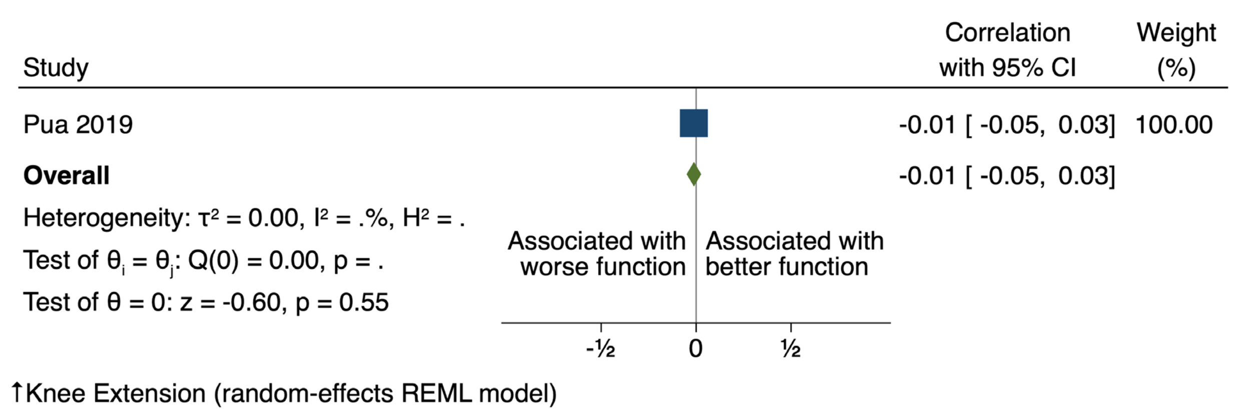

Fig. 18. ↑Knee Flexion

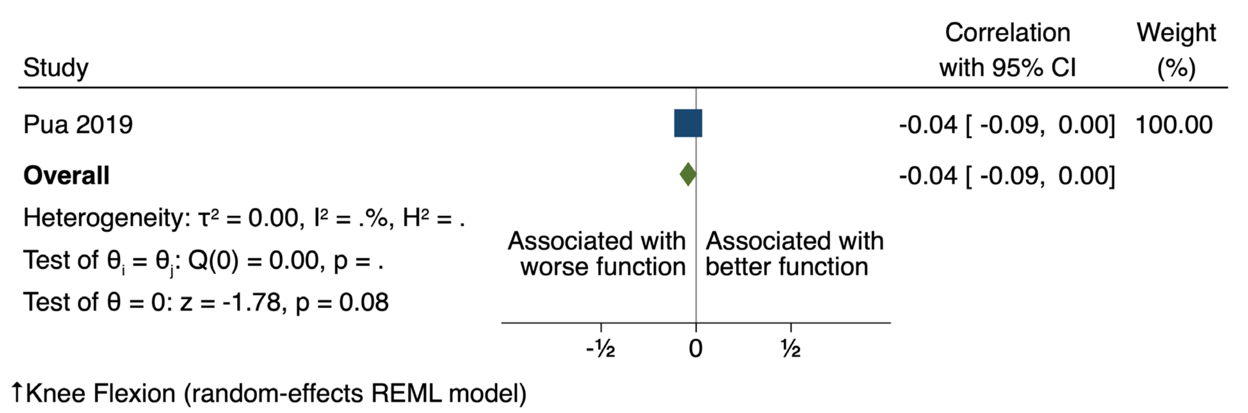

Fig. 19. ↑Age

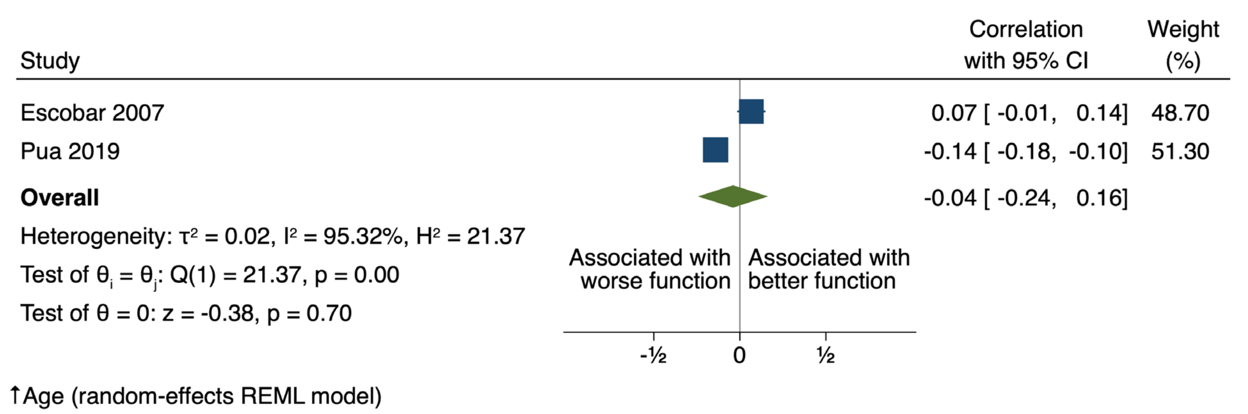

© 2022 Olsen U et al. *JAMA Network Open.*

Fig. 20. ↑Pain Self-Efficacy

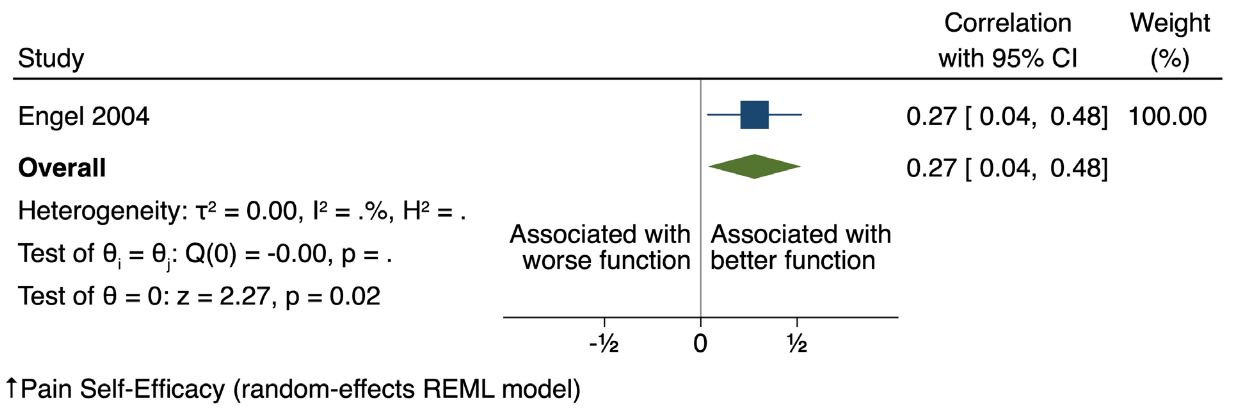

Fig. 21. ↑Preoperative Pain

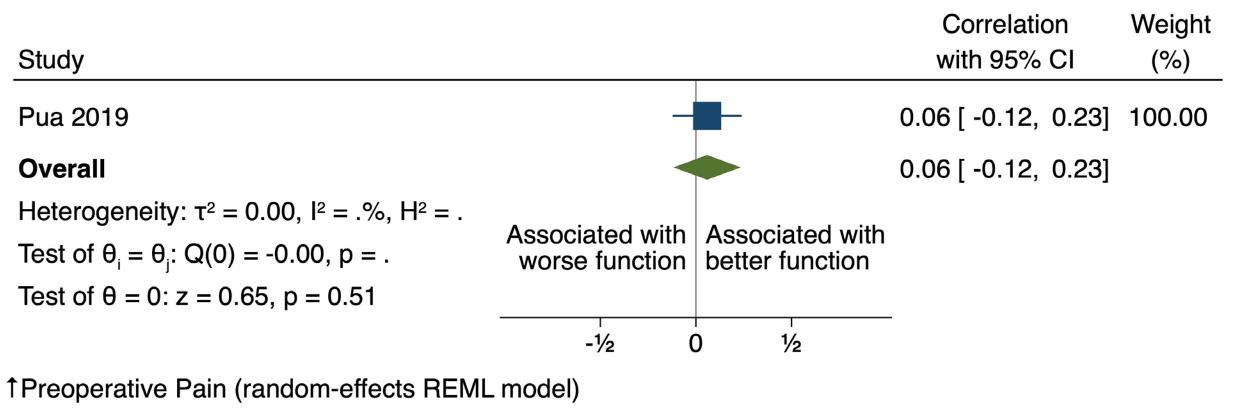

Fig. 22. ↑Social Support

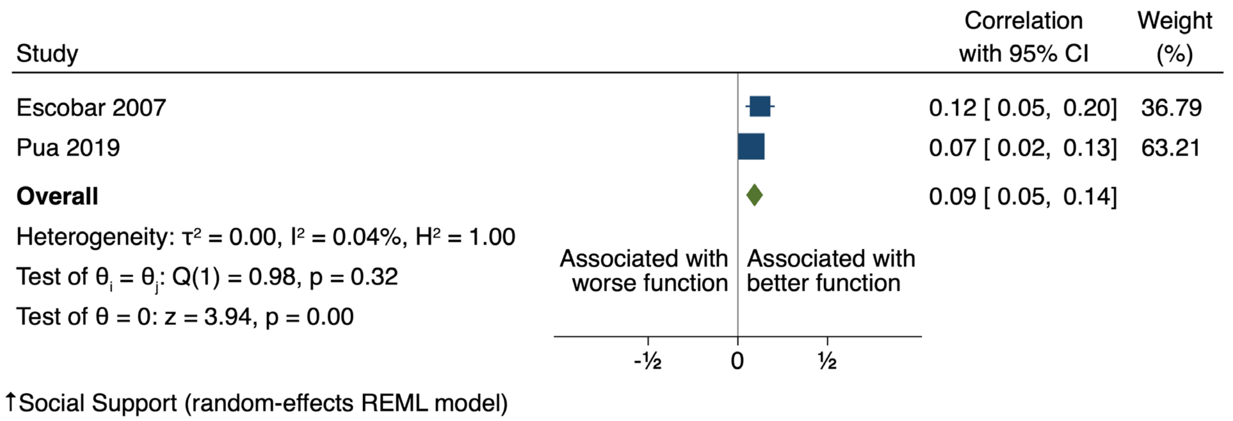

© 2022 Olsen U et al. *JAMA Network Open.*

Fig. 23. Walking Aid Use

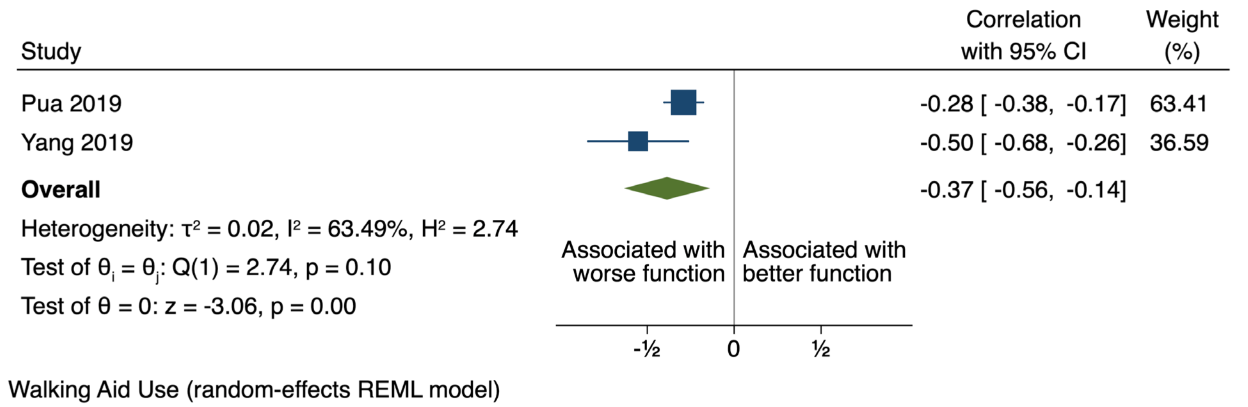

© 2022 Olsen U et al. *JAMA Network Open.*

Function (12 months)

Fig. 24. ↑Knee Status

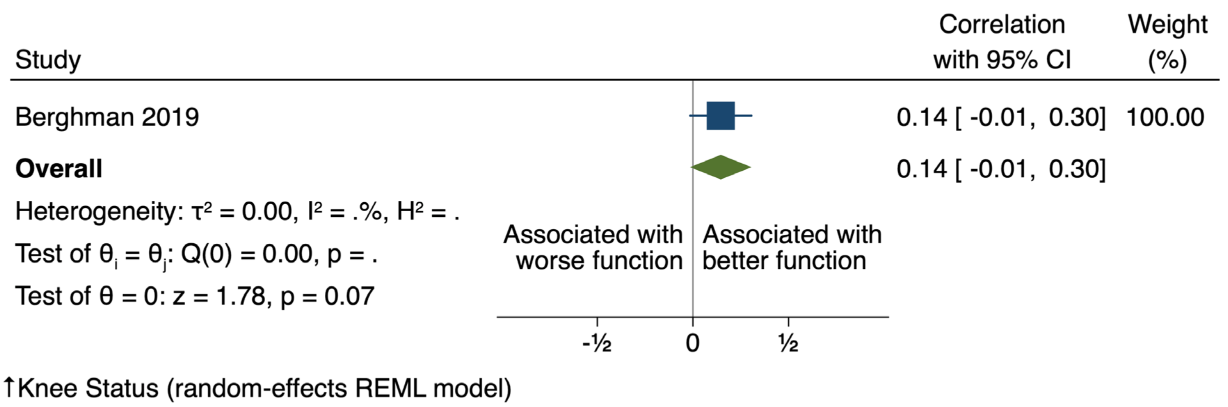

Fig. 25. ↑Mental Health

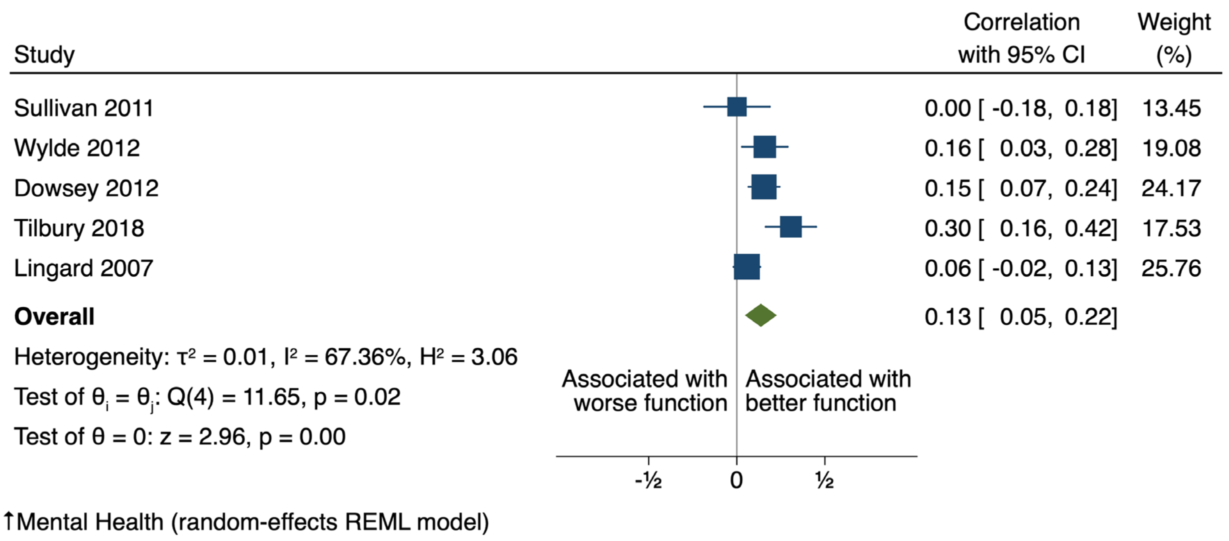

© 2022 Olsen U et al. *JAMA Network Open.*

Fig. 26. ↑Mobility

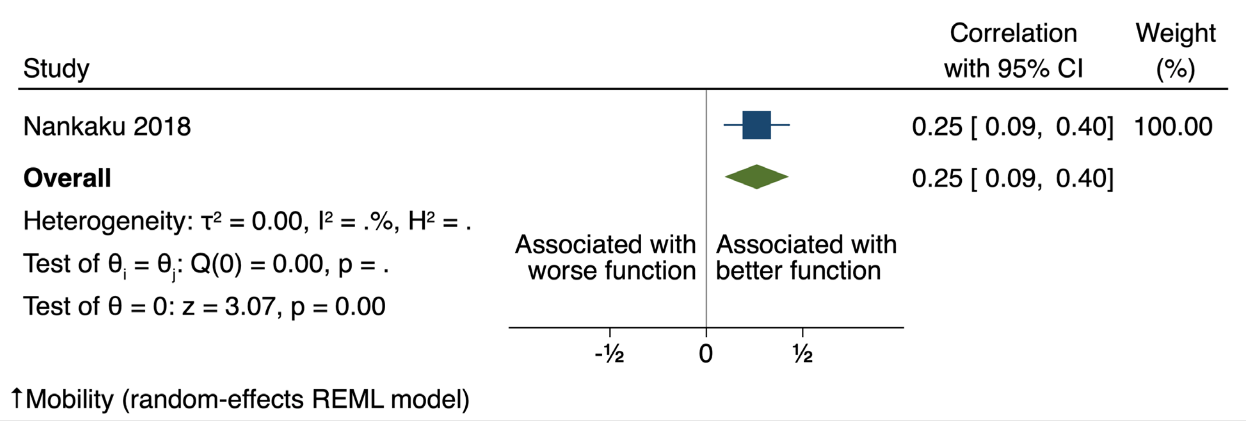

Fig. 27. ↑Outcome Expected

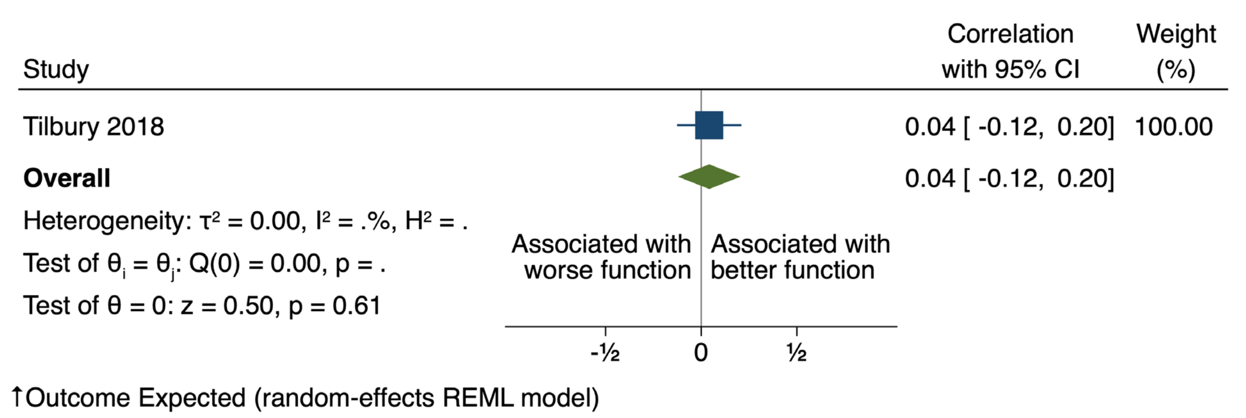

© 2022 Olsen U et al. *JAMA Network Open.*

Fig. 28. ↑Preoperative Function

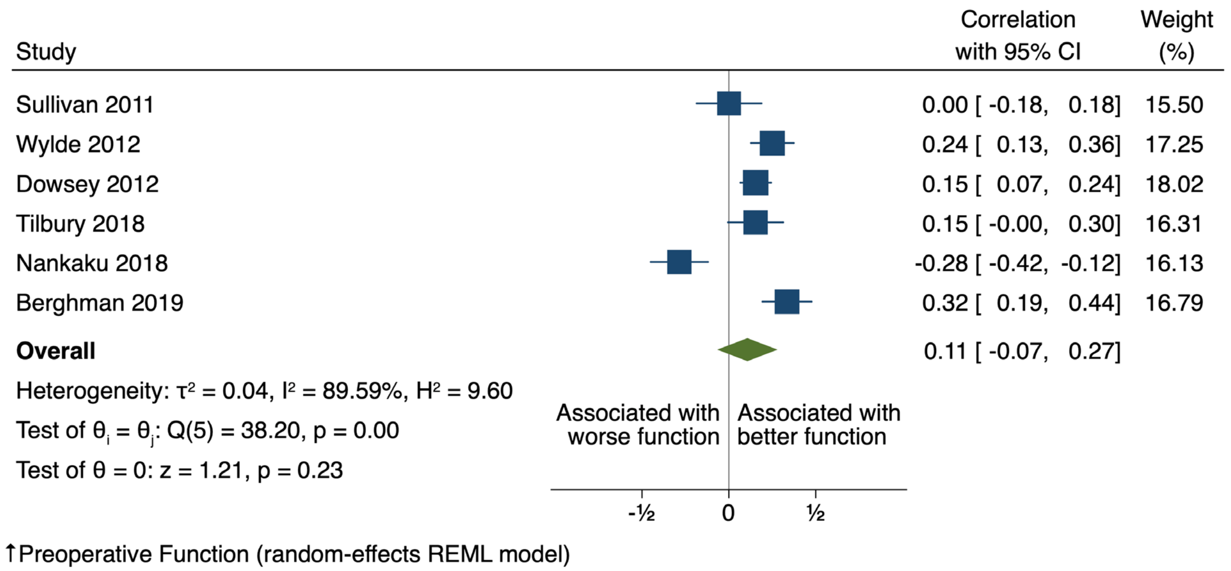

Fig. 29. ↑Catastrophizing

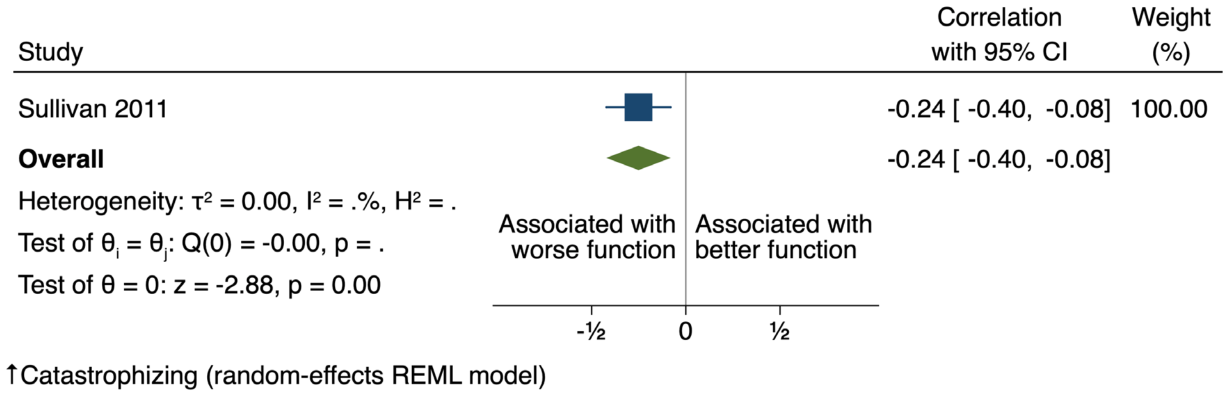

© 2022 Olsen U et al. *JAMA Network Open.*

Fig. 30. ↑Comorbidity

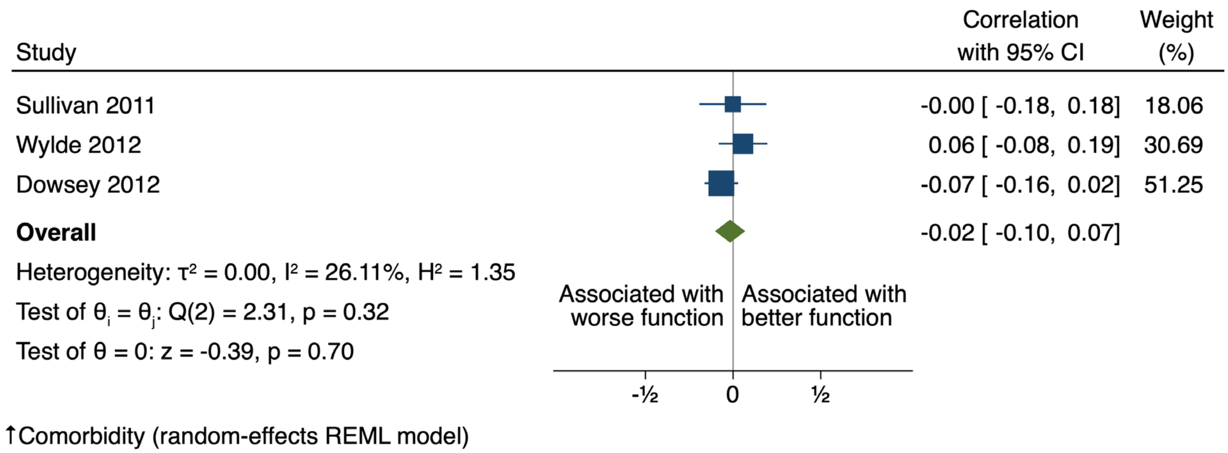

Fig. 31. Cruciate Retaining

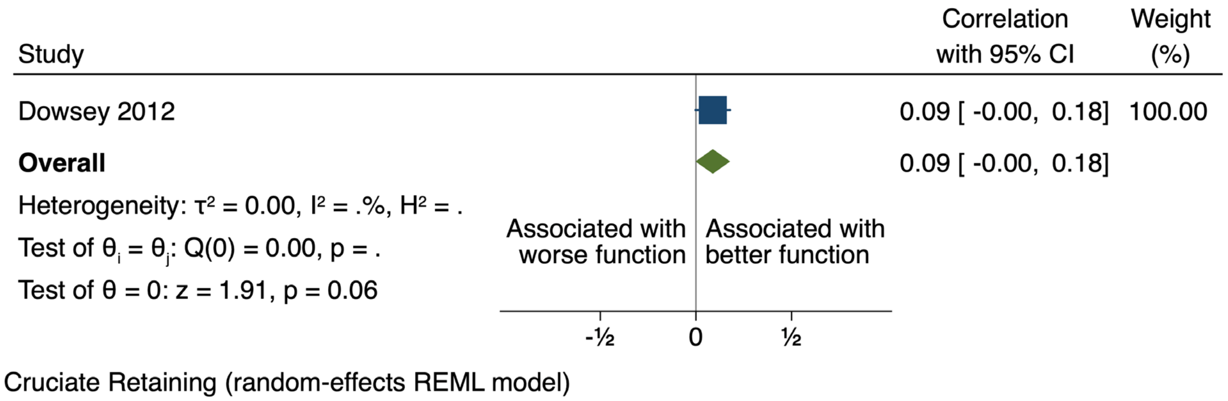

Fig. 32. ↑BMI

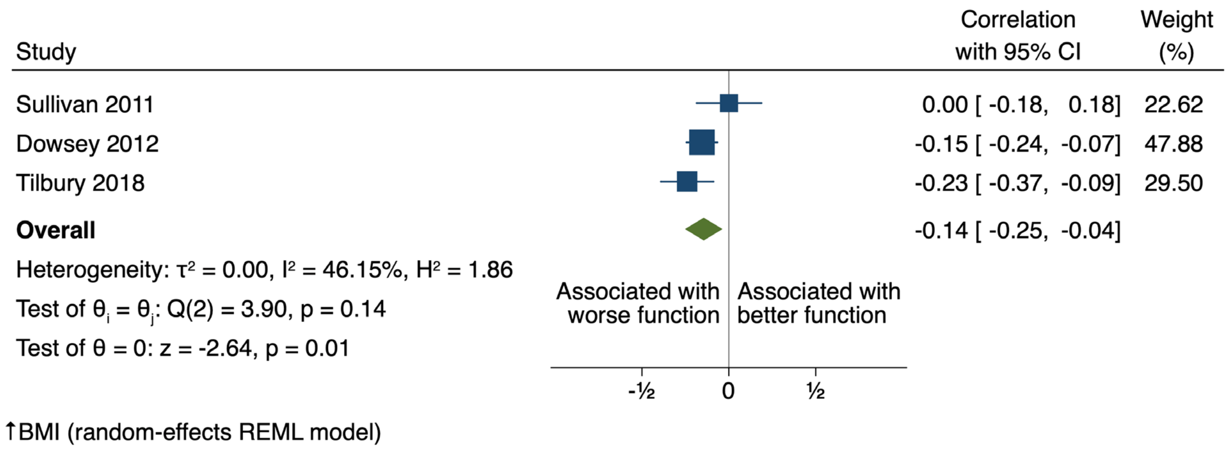

© 2022 Olsen U et al. *JAMA Network Open.*

Fig. 33. ↑K-L Grade

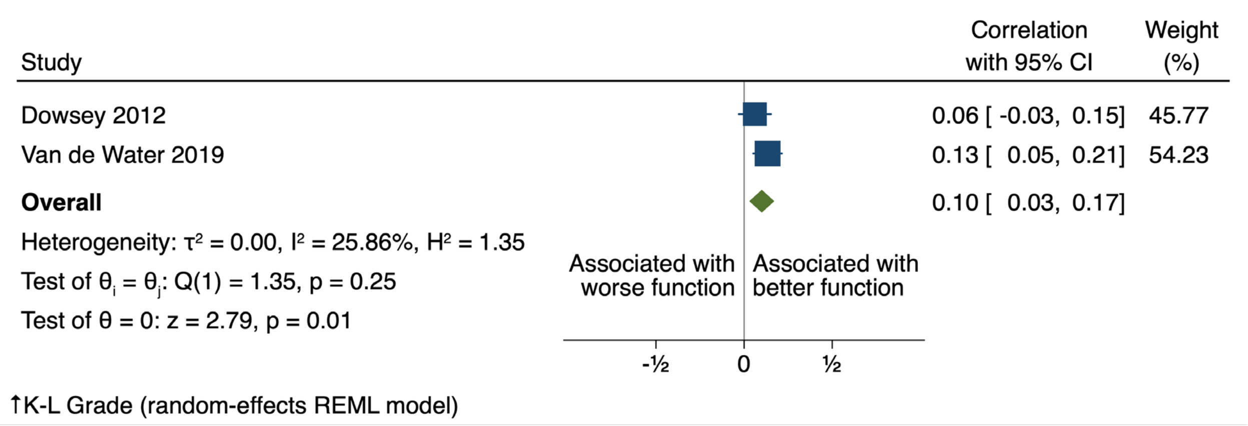

Fig. 34. ↑Kinesophobia

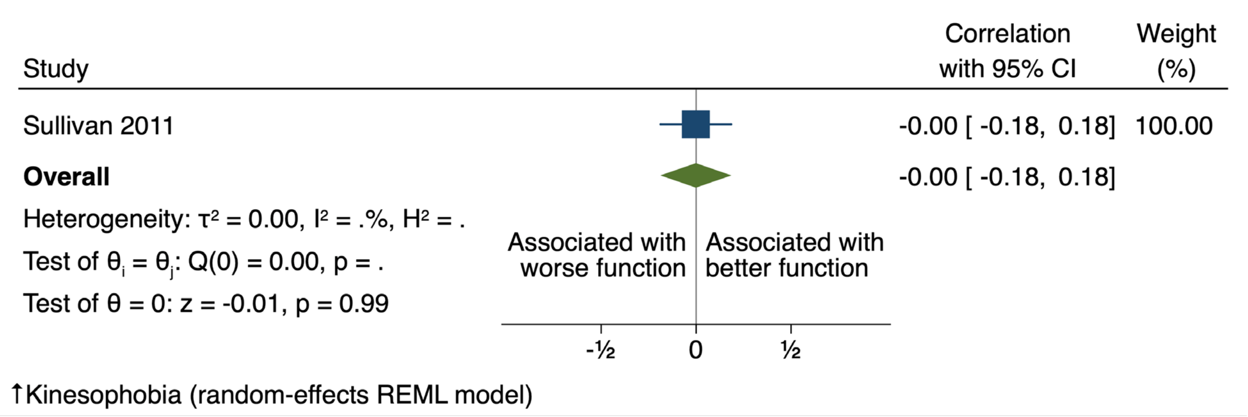

Fig. 35. Male Gender

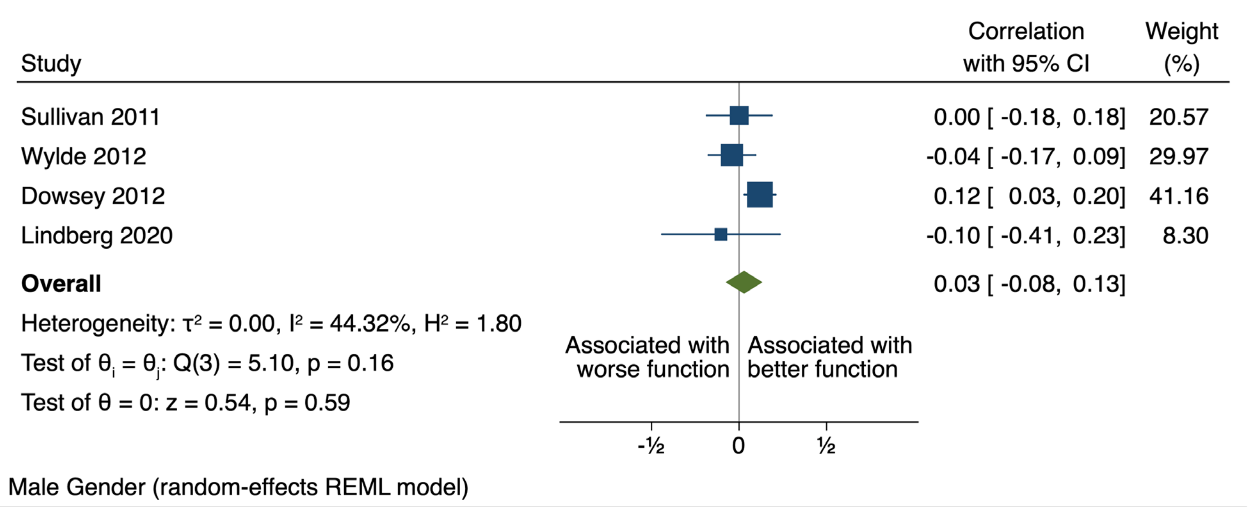

© 2022 Olsen U et al. *JAMA Network Open.*

Fig. 36. Multicompartment OA

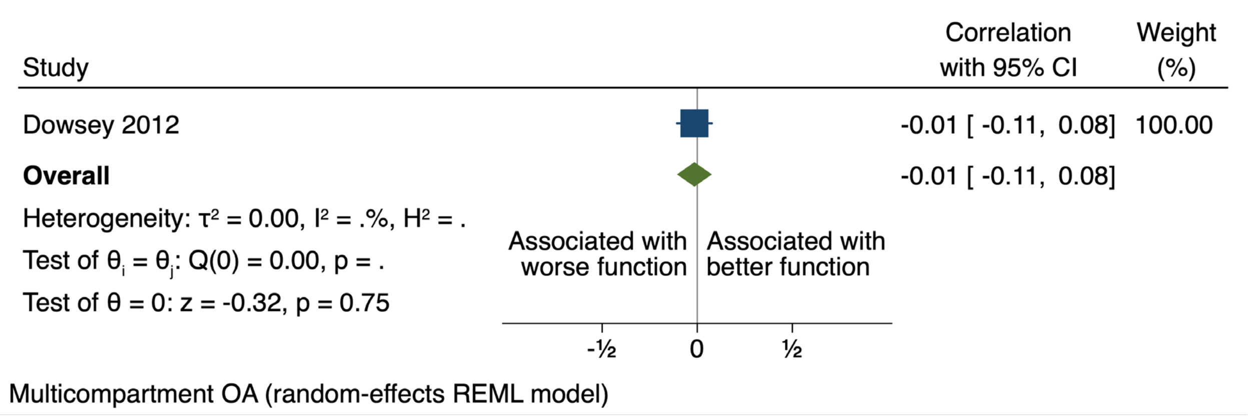

Fig. 37. ↑Age

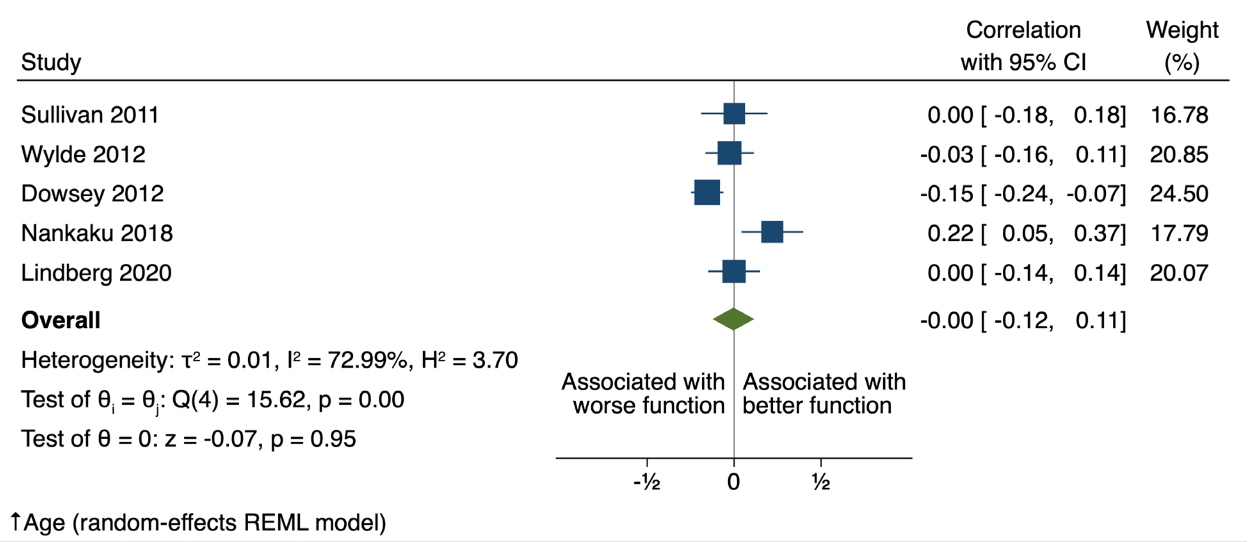

Fig. 38. ↑Pain Self-Efficacy

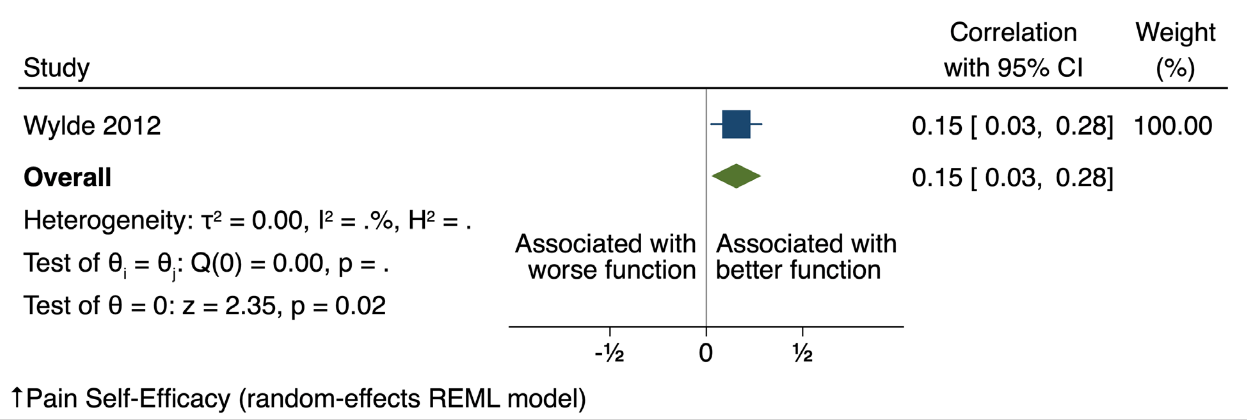

© 2022 Olsen U et al. *JAMA Network Open.*

Fig. 39. Patella Resurfaced

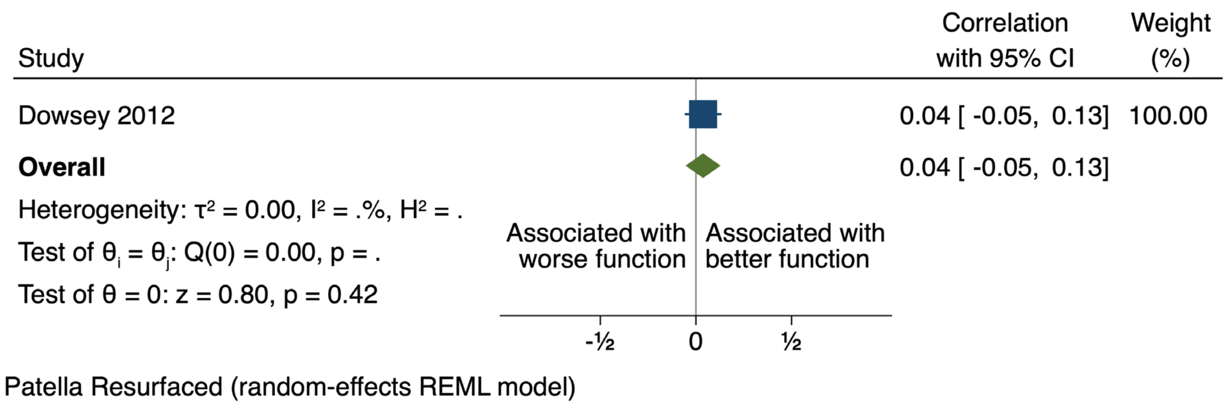

Fig. 40. ↑Preoperative Pain

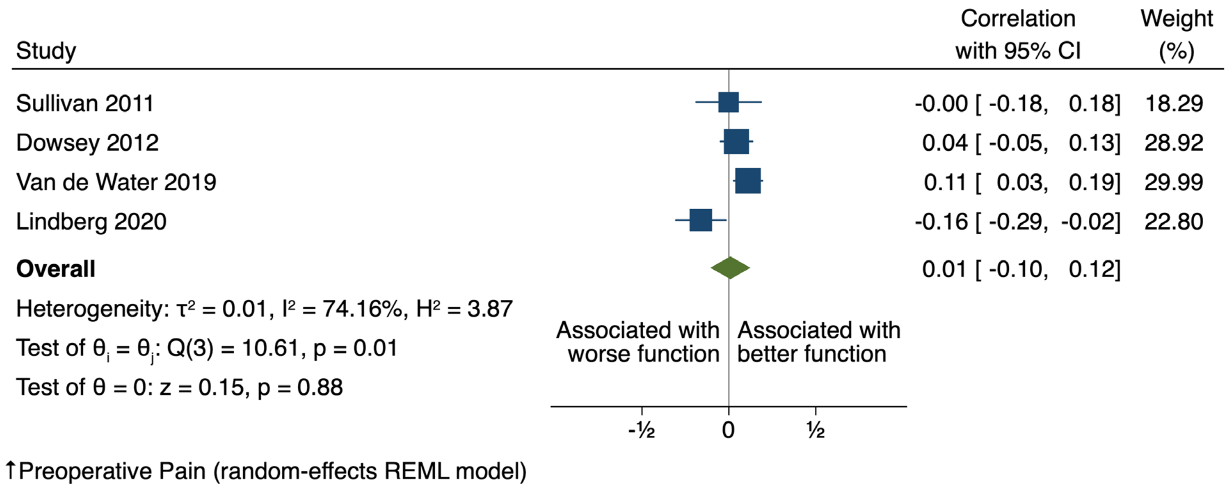

Fig. 41. ↑Surgery Duration

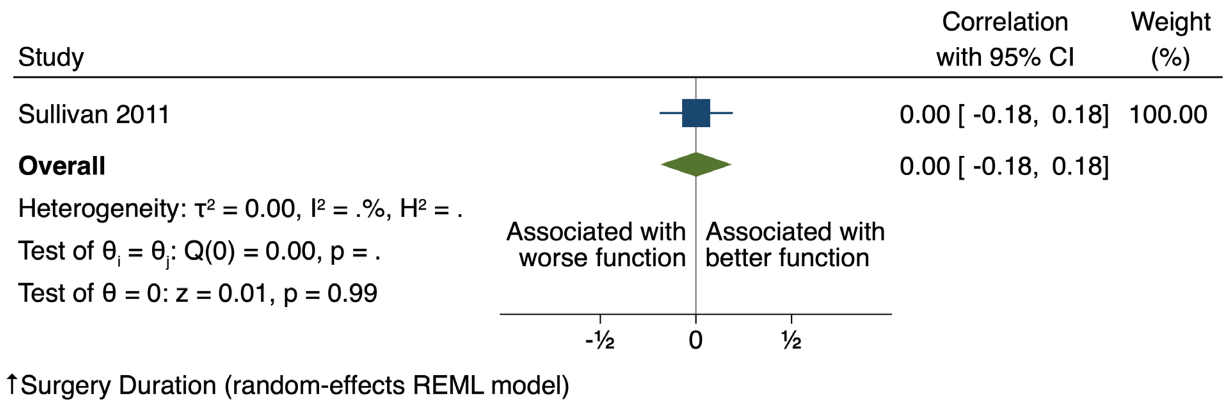

© 2022 Olsen U et al. *JAMA Network Open.*

Fig. 42. ↑Symptomatic Joints

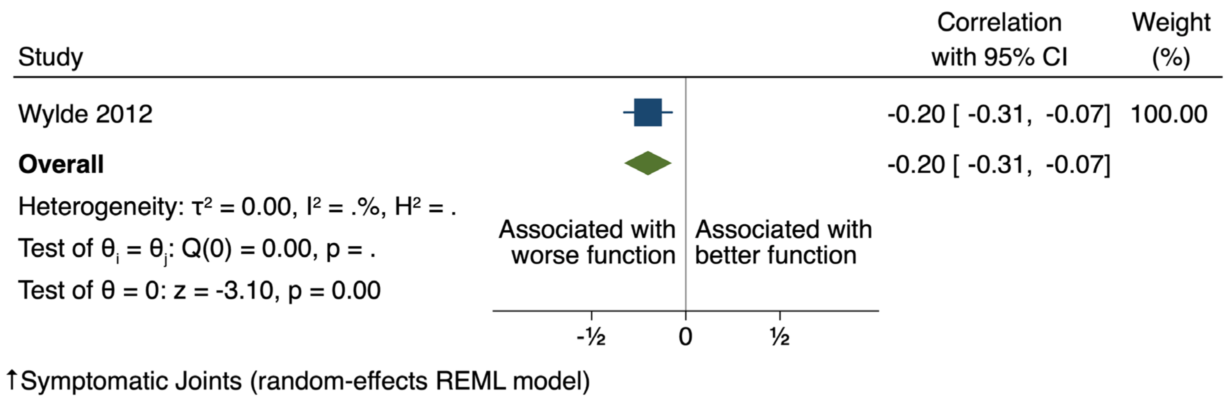

© 2022 Olsen U et al. *JAMA Network Open.*

eTable 1. Sensitivity Analysis

The following table shows estimates of correlations for each of the sensitivity analyses. Estimates for the main analysis (i.e., no studies omitted) are also shown for comparison.

domain_str	concept	cc	cc_lb	cc_ub
All Studies	↑Knee Status	.1438768	-.0758673	.3503173
All Studies	↑Mental Health	.1220621	-.0145701	.2542162
All Studies	↑Mobility	.2544658	.0522105	.4366684
All Studies	↑Outcome Expected	.0416651	-.2145815	.2925428
All Studies	↑Preoperative Function	.1380555	.0164913	.2555974
All Studies	↑Catastrophizing	-.2446435	-.4701152	.0107985
All Studies	↑Comorbidity	-.0239961	-.1224817	.0749586
All Studies	Cruciate Retaining	.0875719	-.0344355	.2070066
All Studies	↑BMI	-.1489251	-.2449921	-.0499614
All Studies	↑K-L Grade	.1004358	.0114511	.1878413
All Studies	↑Kinesophobia	-.0012078	-.2332215	.2309341
All Studies	Male Gender	.0535947	-.0433122	.1494959
All Studies	Multicompartment OA	-.0148018	-.1373228	.1081642
All Studies	↑Age	-.046835	-.1330566	.0400981
All Studies	↑Pain Self-Efficacy	.1537482	-.0133514	.3124918
All Studies	Patella Resurfaced	.0371242	-.085861	.1589939
All Studies	↑Preoperative Pain	.0398254	-.042381	.1214909
All Studies	↑Surgery Duration	.0012099	-.230934	.2332215
All Studies	↑Symptomatic Joints	-.1970977	-.3492267	-.0348474
Study Participation	↑Knee Status	.1438074	-.0972522	.3690755
Study Participation	↑Mental Health	.1259386	-.0829971	.3242769
Study Participation	↑Mobility	.2544397	-.0698999	.5301681
Study Participation	↑Outcome Expected	.0416674	-.2804281	.3553225
Study Participation	↑Preoperative Function	.125238	.0183123	.2294473
Study Participation	↑Comorbidity	-.0664894	-.1990565	.0685877
Study Participation	Cruciate Retaining	.0875113	-.0469377	.2189611

© 2022 Olsen U et al. *JAMA Network Open.*

Study Participation	↑BMI	-.1736704	-.2875198	-.0548615
Study Participation	↑K-L Grade	.1003758	-.005034	.2036943
Study Participation	Male Gender	.1036663	-.0266921	.2306734
Study Participation	Multicompartment OA	-.0148631	-.149485	.120419
Study Participation	↑Age	-.0589676	-.1708215	.0545022
Study Participation	Patella Resurfaced	.037063	-.0981895	.1710886
Study Participation	↑Preoperative Pain	.0434701	-.0582769	.1444436
Study Attrition	↑Knee Status	.1439137	-.1031806	.3742375
Study Attrition	↑Mental Health	.153948	-.0984798	.3877874
Study Attrition	↑Preoperative Function	.2131116	.0054118	.4031501
Study Attrition	↑Comorbidity	-.0276633	-.2746447	.2226636
Study Attrition	Cruciate Retaining	.0875735	-.0501764	.22205
Study Attrition	↑BMI	-.152584	-.2818696	-.017899
Study Attrition	↑K-L Grade	.1004379	-.0091389	.2076262
Study Attrition	Male Gender	.0615559	-.0590784	.1804243
Study Attrition	Multicompartment OA	-.0147903	-.1526335	.1235935
Study Attrition	↑Age	-.0909527	-.2027268	.0231506
Study Attrition	↑Pain Self-Efficacy	.1537942	-.0251349	.3230892
Study Attrition	Patella Resurfaced	.0371306	-.1013832	.1742187
Study Attrition	↑Preoperative Pain	.0435376	-.0625176	.148609
Study Attrition	↑Symptomatic Joints	-.1971193	-.3597354	-.0228384
Prognostic Factor Measurement	↑Knee Status	.1438768	-.0758673	.3503173
Prognostic Factor Measurement	↑Mental Health	.1220621	-.0145701	.2542162
Prognostic Factor Measurement	↑Mobility	.2544658	.0522105	.4366684
Prognostic Factor Measurement	↑Outcome Expected	.0416651	-.2145815	.2925428
Prognostic Factor Measurement	↑Preoperative Function	.1380555	.0164913	.2555974
Prognostic Factor Measurement	↑Catastrophizing	-.2446435	-.4701152	.0107985
Prognostic Factor Measurement	↑Comorbidity	-.0239961	-.1224817	.0749586

© 2022 Olsen U et al. *JAMA Network Open.*

Prognostic Factor Measurement	Cruciate Retaining	.0875719	-.0344355	.2070066
Prognostic Factor Measurement	↑BMI	-.1489251	-.2449921	-.0499614
Prognostic Factor Measurement	↑K-L Grade	.1004358	.0114511	.1878413
Prognostic Factor Measurement	↑Kinesophobia	-.0012078	-.2332215	.2309341
Prognostic Factor Measurement	Male Gender	.0535947	-.0433122	.1494959
Prognostic Factor Measurement	Multicompartment OA	-.0148018	-.1373228	.1081642
Prognostic Factor Measurement	↑Age	-.046835	-.1330566	.0400981
Prognostic Factor Measurement	↑Pain Self-Efficacy	.1537482	-.0133514	.3124918
Prognostic Factor Measurement	Patella Resurfaced	.0371242	-.085861	.1589939
Prognostic Factor Measurement	↑Preoperative Pain	.0398254	-.042381	.1214909
Prognostic Factor Measurement	↑Surgery Duration	.0012099	-.230934	.2332215
Prognostic Factor Measurement	↑Symptomatic Joints	-.1970977	-.3492267	-.0348474
Outcome Measurement	↑Knee Status	.1438768	-.0758673	.3503173
Outcome Measurement	↑Mental Health	.1220621	-.0145701	.2542162
Outcome Measurement	↑Mobility	.2544658	.0522105	.4366684
Outcome Measurement	↑Outcome Expected	.0416651	-.2145815	.2925428
Outcome Measurement	↑Preoperative Function	.1380555	.0164913	.2555974
Outcome Measurement	↑Catastrophizing	-.2446435	-.4701152	.0107985
Outcome Measurement	↑Comorbidity	-.0239961	-.1224817	.0749586
Outcome Measurement	Cruciate Retaining	.0875719	-.0344355	.2070066
Outcome Measurement	↑BMI	-.1489251	-.2449921	-.0499614
Outcome Measurement	↑K-L Grade	.1004358	.0114511	.1878413
Outcome Measurement	↑Kinesophobia	-.0012078	-.2332215	.2309341

© 2022 Olsen U et al. *JAMA Network Open.*

Outcome Measurement	Male Gender	.0535947	-.0433122	.1494959
Outcome Measurement	Multicompartment OA	-.0148018	-.1373228	.1081642
Outcome Measurement	↑Age	-.046835	-.1330566	.0400981
Outcome Measurement	↑Pain Self-Efficacy	.1537482	-.0133514	.3124918
Outcome Measurement	Patella Resurfaced	.0371242	-.085861	.1589939
Outcome Measurement	↑Preoperative Pain	.0398254	-.042381	.1214909
Outcome Measurement	↑Surgery Duration	.0012099	-.230934	.2332215
Outcome Measurement	↑Symptomatic Joints	-.1970977	-.3492267	-.0348474
Study Confounding	↑Knee Status	.1438906	-.081434	.3552223
Study Confounding	↑Mental Health	.1220604	-.0195056	.2588282
Study Confounding	↑Outcome Expected	.0416636	-.1608326	.240794
Study Confounding	↑Preoperative Function	.1863531	.0503854	.315535
Study Confounding	↑Catastrophizing	-.2446464	-.4773555	.0201301
Study Confounding	↑Comorbidity	-.0239955	-.1998238	.153332
Study Confounding	Cruciate Retaining	.0875715	-.0368199	.2092905
Study Confounding	↑BMI	-.1489248	-.2477128	-.0470718
Study Confounding	↑K-L Grade	.1004353	.0082223	.1909545
Study Confounding	↑Kinesophobia	-.0012089	-.2344216	.2321355
Study Confounding	Male Gender	.0535913	-.0462903	.152412
Study Confounding	Multicompartment OA	-.0148024	-.1396434	.1105018
Study Confounding	↑Age	-.0808168	-.173188	.0129638
Study Confounding	↑Pain Self-Efficacy	.1537472	-.0151047	.3140733
Study Confounding	Patella Resurfaced	.0371236	-.0882123	.1613024

© 2022 Olsen U et al. *JAMA Network Open.*

Study Confounding	↑Preoperative Pain	.0398227	-.0458679	.1249315
Study Confounding	↑Surgery Duration	.0012087	-.2321355	.2344216
Study Confounding	↑Symptomatic Joints	-.1970987	-.3507967	-.0330603
Statistical Analysis & Reporting	↑Knee Status	.1438709	-.0878438	.3608505
Statistical Analysis & Reporting	↑Mental Health	.1305	-.0422659	.2955705
Statistical Analysis & Reporting	↑Outcome Expected	.041638	-.2604262	.3363232
Statistical Analysis & Reporting	↑Preoperative Function	.2046128	.0356259	.3622169
Statistical Analysis & Reporting	↑Comorbidity	-.0277205	-.141957	.0872761
Statistical Analysis & Reporting	Cruciate Retaining	.0875583	-.0432345	.2154257
Statistical Analysis & Reporting	↑BMI	-.1736245	-.2836936	-.0590136
Statistical Analysis & Reporting	↑K-L Grade	.1004227	-.0002653	.1991194
Statistical Analysis & Reporting	Male Gender	.0615529	-.0510875	.172659
Statistical Analysis & Reporting	Multicompartment OA	-.0148157	-.1458837	.1167897
Statistical Analysis & Reporting	↑Age	-.0909736	-.1945621	.0146535
Statistical Analysis & Reporting	↑Pain Self-Efficacy	.1537341	-.0198638	.3183579
Statistical Analysis & Reporting	Patella Resurfaced	.0371104	-.0945377	.1675086
Statistical Analysis & Reporting	↑Preoperative Pain	.0435219	-.0533316	.1395834
Statistical Analysis & Reporting	↑Symptomatic Joints	-.1971116	-.3550482	-.0282085

© 2022 Olsen U et al. *JAMA Network Open.*

eTable 2. Reported Associations at 3 mo After TKA

Author, reference, year	Prognostic factor	Outcome	Published estimate
Lingard et al,[25] 2007	Distress/ physical function (SF-36 MH)	Womac function	LSM 3·5; p=0·14.
Berghmans et al,[39] 2019	Mental health (SF-12 mental health)	WOMAC function	B 0.27, CI95% 0.22 to 0.66, SE 0.11, p=0.00
Berghmans et al,[39] 2019	Preoperative function (WOMAC)	WOMAC function	B 0.44, SE 0.11, CI 95% 0.22 to 0.66, p=0.00
Lindner et al,[36] 2018	Preoperative function (WOMAC)	WOMAC function	β 0·45,SE B, β0.45, t 3·65; p=0·001

© 2022 Olsen U et al. *JAMA Network Open.*

eTable 3. Definition and Labels of Factors*

Predictor Name	Definition
↑Age	Older age
Male Gender	Male (rather than female) gender
↑Preoperative Pain	More (worse) pain
↑Comorbidity	More comorbidities
↑BMI	Higher (worse) body mass index
↑Catastrophizing	More (worse) catastrophizing
↑Social Support	Better social support
↑Low Back Pain	More (worse) low back pain
↑Mental Health	Better (improved) mental health
↑Contralateral Knee Pain	More (worse) contralateral knee pain
↑Education	Higher educational attainment
Indian Ethnicity	Indian (rather than Chinese) Ethnicity
↑Knee Extension	Greater (better) knee extension
↑Preoperative Function	Better preoperative physical function
↑Preoperative knee status	Better knee status
↑Mobility	Better mobility
↑Pain Self-Efficacy	More (better) pain self-efficacy
↑Symptomatic Joints	More symptomatic joints
↑Kinesophobia	More (worse) kinesophobia
↑Surgery Duration	Longer surgery duration
Multicompartment OA	Multicompartment OA
↑K-L Grade	Higher (worse) Kellgren-Lawrence grade
Patella Resurfaced	Patella Resurfaced surgery
↑Outcome Expectation	Better outcome expected
Walking aid use	Walking aid use
↑Arthritis Helplessness	More (worse) arthritis helplessnes
Cruciate retaining surgery	Cruciate retaining surgery
↓Energy	Less energy
↑ Drowsiness	More drowsiness
↑ Bloating	More bloating
↑ Worrying	More worrying
↑ Problems sexuality	More problems sexuality
↑ Sleep dysfunction	Worse sleep dysfunction (PSQI)
↑ Day time sleepiness	More daytime sleepiness
↑ Sleep quality	Better sleep quality
↓ Sedentary behaviour	Less sedentary behaviour

*Labels for factors: direction of association is indicated by arrows, with ↑ symbol indicating "higher value of"; e.g., "↑Age" should be interpreted as "older age").

© 2022 Olsen U et al. *JAMA Network Open.*

eTable 4. Grading of Recommendation Assessment, Development and Evaluation

4a

№ of studies (Reference number)	Certainty assessment at twelve months follow-up						Certainty of evidence with explanations for downgrading of evidence
	Study design	Risk of bias	Inconsistency	Indirectness	Imprecision	Other considerations	
**Age**							
5 (23, 25, 26, 29, 39)	observational studies	serious[a]	serious[b]	not serious	not serious	none	⊕⊕⊕◯ MODERATE<br>[a]Two studies with high risk of bias at two or more domains. [b]Statistical heterogeneity and inconsistency in direction of effect.
**Male gender**							
4 (23, 25, 26, 29, 39)	observational studies	serious[c]	not serious	not serious	not serious	none	⊕⊕⊕◯ MODERATE<br>[c]Two studies with high risk of bias at one or more domains.
**Preoperative pain**							
3 (23, 26, 28, 39)	observational studies	not serious	serious	not serious	not serious	none	⊕⊕⊕◯ MODERATE<br>Statistical heterogeneity and inconsistency in direction of effect.
**Comorbidity**							
3 (23, 26, 29)	observational studies	serious[d]	not serious	not serious	not serious	none	⊕⊕⊕◯ MODERATE<br>[d]Two studies with high risk of bias at one or more domains.
**Higher BMI**							
3 (23, 26, 27)	observational studies	serious[e]	not serious	not serious	not serious	none	⊕⊕⊕◯MODERATE<br>[e]Two studies with high risk of bias at one or more domains.
**Catastrophizing**							

© 2022 Olsen U et al. *JAMA Network Open.*

№ of studies (Reference number)	Certainty assessment at twelve months follow-up						Certainty of evidence with explanations for downgrading of evidence
	Study design	Risk of bias	Inconsistency	Indirectness	Imprecision	Other considerations	
1 (27)	observational studies	serious[f]	not serious	not serious	very serious[g]	none	⊕◯◯◯ VERY LOW<br>[f]One study with three domains rated with high risk of bias. [g]One study and small sample size (n=120)
**Mental health**							
5 (23, 24, 26, 27, 29)	observational studies	serious[h]	not serious	not serious	not serious	none	⊕⊕⊕ MODERATE<br>[h]High risk of bias on several domain from several studies.
**Preoperative function**							
5 (23,25-27, 29, 38)	observational studies	serious[i]	serious[j]	not serious	not serious	none	⊕⊕◯◯ LOW<br>[i]Two studies with high risk of bias at two or more domains. [j]Statistical heterogeneity and inconsistency in direction of effect.
**Mobility**							
1 (25)	observational studies	serious[k]	not serious	not serious	very serious[l]	none	⊕◯◯◯ VERY LOW<br>[k]One study with three domains rated with high risk of bias. [l]One study with small sample size (n=115) and wide confidence interval
**Pain self-efficacy**							
1 (29)	serious[m]	not serious	not serious	not serious	very serious[n]	none	⊕◯◯◯ VERY LOW<br>[m]High risk of bias at one domain. [n]One study and small sample size.
**Symptomatic joints**							
1 (29)	not serious	not serious	not serious	not serious	very serious[o]	none	⊕⊕◯◯ LOW<br>[o]Estimate based on one study (n=220).
**Kinesophobia**							

© 2022 Olsen U et al. *JAMA Network Open.*

№ of studies (Reference number)	Certainty assessment at twelve months follow-up						Certainty of evidence with explanations for downgrading of evidence
	Study design	Risk of bias	Inconsistency	Indirectness	Imprecision	Other considerations	
1 (26)	observational studies	serious[p]	not serious	not serious	very serious[p]	none	⊕◯◯◯ VERY LOW<br>[p]One study with three domains rated with high risk of bias. [p]Estimate based on one study with small sample size (n=120).
**Surgery duration**							
1 (26)	observational studies	serious[r]	not serious	not serious	very serious[s]	none	⊕◯◯◯ VERY LOW<br>[r]One study with three domains rated with high risk of bias. [s]Estimate based on one study with small sample size (n=120).
**Multicompartment osteoarthritis**							
1 (23)	observational studies	not serious	not serious	not serious	serious[t]	none	⊕⊕⊕ MODERATE<br>[t]Estimated based on one study (n=473)
**K-L grade**							
2 (23,28)	observational studies	not serious	not serious	not serious	not serious	none	⊕⊕⊕⊕ HIGH
**Patella resurfaced**							
1 (23)	observational studies	not serious	not serious	not serious	serious[u]	none	⊕⊕⊕ MODERATE<br>[u]Estimated based on one study (n=473)
**Outcome expected**							
1 (27)	observational studies	not serious	not serious	not serious	very serious[v]	none	⊕⊕◯◯ LOW<br>[v]Estimated based on one study (n=146)
**Cruciate retaining**							
1 (23)	observational studies	not serious	not serious	not serious	serious[w]	none	⊕⊕⊕ MODERATE<br>[w]Estimate based on one study (n=473)
**Knee status**							

© 2022 Olsen U et al. *JAMA Network Open.*

№ of studies (Reference number)	Certainty assessment at twelve months follow-up						Certainty of evidence with explanations for downgrading of evidence
	Study design	Risk of bias	Inconsistency	Indirectness	Imprecision	Other considerations	
1 (38)	observational studies	serious	not serious	not serious	very[x] serious	None	[x] Estimate based on one study (n=144)

References

23. Dowsey MM, Nikpour M, Dieppe P, Choong PF. Associations between pre-operative radiographic changes and outcomes after total knee joint replacement for osteoarthritis. *Osteoarthritis Cartilage* 2012; **20**: 1095-102.
24. Lingard EA, Riddle DL. Impact of psychological distress on pain and function following knee arthroplasty. *J Bone Joint Surg Am* 2007; **89**: 1161-9.
25. Nankaku M, Ito H, Furu M, et al. Preoperative factors related to the ambulatory status at 1 year after total knee arthroplasty. *Disabil Rehabil* 2018; **40**: 1929-32.
26. Sullivan M, Tanzer M, Reardon G, Amirault D, Dunbar M, Stanish W. The role of presurgical expectancies in predicting pain and function one year following total knee arthroplasty. *Pain* 2011; **152**: 2287-93.
27. Tilbury C, Haanstra TM, Verdegaal SHM, et al. Patients' pre-operative general and specific outcome expectations predict postoperative pain and function after total knee and total hip arthroplasties. *Scand J Pain* 2018; **18**: 457-66.
28. van de Water RB, Leichtenberg CS, Nelissen RG, et al. Preoperative Radiographic Osteoarthritis Severity Modifies the Effect of Preoperative Pain on Pain/Function After Total Knee Arthroplasty: Results at 1 and 2 Years Postoperatively. *JBJS* 2019; **101**: 879-87.
29. Wylde V, Dixon S, Blom AW. The role of preoperative self-efficacy in predicting outcome after total knee replacement. *Musculoskeletal Care* 2012; **10**: 110-8. 32.
38. Berghmans DDP, Lenssen AF, Emans PJ, van Rhijn LW, de Bie RA. Limited predictive value of pre-surgical level of functioning for functioning at 3 and 12 months after TKA. *Knee Surg Sports Traumatol Arthrosc*. May 2019;27(5):1651-1657. doi:10.1007/s00167-018-5288-5
39. Lindberg MF, Schweitz TU, Aamodt A, Gay C, Lerdal A. High pre- and postoperative symptom burden in non-responders to total knee arthroplasty. *PLoS One*. 2020;15(5):e0233347. doi:10.1371/journal.pone.02333471.

© 2022 Olsen U et al. *JAMA Network Open.*

eTable 4. Grading of Recommendation Assessment, Development and Evaluation

4b

№ of studies (Reference)	Certainty assessment six months follow-up						Certainty of evidence with explanations for downgrading of evidence
	Study design	Risk of bias	Inconsistency	Indirectness	Imprecision	Other considerations	
**Age**							
2 (31, 32)	observational	serious[a]	serious[b]	not serious	not serious	none	⊕⊕◯◯ LOW<br>[a]One study with high risk of bias at two domains inical and statistical heterogeinity. [b]Clinical and statistical heterogeinity
**Male gender**							
2 (31, 32)	observational	not serious	not serious	not serious	not serious	none	⊕⊕⊕⊕ HIGH
**Preoperative pain**							
1 (32)	observational	not serious	not serious	not serious	serious[c]	none	⊕⊕⊕◯ MODERATE<br>[c] Estimate based on one study (n= 4026)
**Comorbidity**							
3 (30,31,32)	observational	serious[d]	not serious	not serious	not serious	none	⊕⊕⊕◯ MODERATE<br>[d]Two studies with high risk of bias at one or more domains
**BMI**							
1 (32)	observational	not serious	not serious	not serious	serious[e]	none	⊕⊕⊕◯ MODERATE<br>[e]Estimate based on one study (n= 4026)
**Catastrophizing**							
2 (36,42)	observational	serious[f]	not serious	not serious	very serious[g]	none	⊕◯◯◯ VERY LOW<br>[f]One study with high risk of bias at one domain. [g]Estimate with imprecise estimated, based on one single study (n=131)
**Social support**							

© 2022 Olsen U et al. *JAMA Network Open.*

№ of studies (Reference)	Certainty assessment six months follow-up						Certainty of evidence with explanations for downgrading of evidence
	Study design	Risk of bias	Inconsistency	Indirectness	Imprecision	Other considerations	
2 (31,32)	observational studies	not serious	not serious	not serious	not serious	none	⊕⊕⊕⊕ HIGH
**Low back pain**							
1 (31)	observational studies	serious[h]	not serious	not serious	serious[i]	none	⊕⊕◯◯ LOW<br>[h]One study rated with high risk of bias at two domains. [i]Estimate based on one large study (n=640)
**Mental health**							
2 (31, 32, 42)	observational	not serious	not serious	not serious	not serious	none	⊕⊕⊕⊕ HIGH
**Contralateral knee pain**							
1 (32)	observational	not serious	not serious	not serious	serious[j]	none	⊕⊕⊕◯ MODERATE<br>[j]Estimate based on one study (n=4026)
**Education**							
1 (32)	observational	not serious	not serious	not serious	serious[k]	none	⊕⊕⊕◯ MODERATE<br>[k]Estimate based on one study (n= 4026)
**Indian ethnicity**							
1 (32)	observational	not serious	not serious	not serious	serious[l]	none	⊕⊕⊕◯ MODERATE<br>[l]Estimate based on one study (n= 4026)
**Knee extension**							
1 (32)	observational	not serious	not serious	not serious	serious[m]	none	⊕⊕⊕◯ MODERATE<br>[m]Estimate based on one study (n= 4026)
**Preoperative function**							
3 (31-33)	observational	serious[n]	not serious	not serious	not serious	none	⊕⊕⊕◯ MODERATE<br>[n]Two studies rated with high risk of bias at two or more domains
**Mobility**							

© 2022 Olsen U et al. *JAMA Network Open.*

№ of studies (Reference)	Certainty assessment six months follow-up						Certainty of evidence with explanations for downgrading of evidence
	Study design	Risk of bias	Inconsistency	Indirectness	Imprecision	Other considerations	
1 (34)	observational	not serious	not serious	not serious	very serious[o]	none	⊕⊕◯◯ LOW<br>[o]Estimate based on one study (n=81)
**Pain self-efficacy**							
1 (37)	observational	serious[p]	not serious	not serious	very serious[q]	none	⊕◯◯◯ VERY LOW<br>[p]High risk on bias on four domains.<br>[q]One study (n=54), statistical imprecise.
**Walking aid use**							
2 (32,42)	observational	not serious	not serious	not serious	serious	none	⊕⊕⊕⊕ HIGH
**Arthritis Helplessness**							
1 (37)	observational	serious[s]	not serious	not serious	very serious[t]		⊕◯◯◯ VERY LOW<br>[s]One study and high risk of bias for several domains.<br>[t]Estimate based on one study (n=54)
**Knee flexion**							
1 (32)	observational	not serious	not serious	not serious	serious[u]	none	⊕⊕⊕◯ MODERATE<br>[u]Estimate based on one study (n= 4026)

References

30. Bugada D, Allegri M, Gemma M, et al. Effects of anaesthesia and analgesia on long-term outcome after total knee replacement: A prospective, observational, multicentre study. *Eur J Anaesthesiol* 2017; **34**: 665-72.

31. Escobar A, Quintana JM, Bilbao A, et al. Effect of patient characteristics on reported outcomes after total knee replacement. *Rheumatology (Oxford)* 2007; **46**: 112-9.

32. Pua YH, Poon CL, Seah FJ, et al. Predicting individual knee range of motion, knee pain, and walking limitation outcomes following total knee arthroplasty. *Acta Orthop* 2019; **90**: 179-86.

33. Sugawara Y, Ishijima M, Kurosawa H, et al. Preoperative timed single leg standing time is associated with the postoperative activity of daily living in aged disabled patients with end-stage knee osteoarthritis at six-months after undergoing total knee arthroplasty. *Mod Rheumatol* 2017; **27**: 326-31.

34. Taniguchi M, Sawano S, Kugo M, Maegawa S, Kawasaki T, Ichihashi N. Physical Activity Promotes Gait Improvement in Patients With Total Knee Arthroplasty. *J Arthroplasty* 2016; **31**: 984-8.

35. Lindner M, Nosseir O, Keller-Pliessnig A, Teigelack P, Teufel M, Tagay S. Psychosocial predictors for outcome after total joint arthroplasty: a prospective comparison of hip and knee arthroplasty. *BMC Musculoskelet Disord.* May 22, 2018. doi:10.1186/s12891-018-2058-y. (Assessed December 16, 2020).

© 2022 Olsen U et al. *JAMA Network Open.*

36. Hylkema TH, Stevens M, Selzer F, Amick BA, Katz JN, Brouwer S. Activity Impairment and Work Productivity Loss After Total Knee Arthroplasty: A Prospective Study. *J Arthroplasty* 2019; **34**: 2637-45.
37. Engel C, Hamilton NA, Potter PT, Zautra AJ. Impact of two types of expectancy on recovery from total knee replacement surgery (TKR) in adults with osteoarthritis. *Behav Med* 2004; **30**: 113-23.
42. Yang HY, Losina E, Lange JK, Katz JN, Collins JE. Longitudinal Trajectories of Pain and Function Improvement Following Total Knee Replacement. *ACR Open Rheumatol*. Jul 2019;1(5):308-317. doi:10.1002/acr2.1041

© 2022 Olsen U et al. *JAMA Network Open.*

eTable 5. Search Strategy

Ovid MEDLINE(R) ALL 1946 to October 04, 2021
Search date: 05.10.2021
Result of search: 6577
Search Strategy:

#	Searches	Results
1	Arthroplasty, Replacement, Knee/	27391
2	(tkr or tjkr or tka or tjka).tw,kf.	15451
3	(knee* adj3 (arthroplast* or replacement*)).tw,kf.	36674
4	(total adj2 knee*).tw,kf.	29088
5	(knee* adj2 prosthes*).tw,kf.	3084
6	or/1-5	43063
7	risk/ or risk factors/ or logistic models/ or protective factors/ or risk assessment/	1292658
8	prognosis/ or (prognos* or risk* or predict*).tw,kf.	4462045
9	(preoperative factor* or pre operative factor* or protective factor*).tw,kf.	24813
10	or/7-9	4839127
11	and/6,10	11731
12	(pain adj3 (post* or ongoing or on going or long* or persist* or prolong* or after or follow*)).tw,kw.	102501
13	pain, postoperative/	43031
14	(Pain/ or chronic pain/ or musculoskeletal pain/) and (post* or ongoing or on going or long* or persist* or prolonged or after or follow*).tw,kf.	68062
15	cohort studies/ or follow-up studies/ or longitudinal studies/ or prospective studies/ or retrospective studies/	2219694
16	pain.tw,kf.	685484
17	and/15-16	125324
18	or/12-14,17	257072
19	and/11,18	1629
20	(function* or stiffness or contracture*).tw,kf.	4074115
21	(muscle adj3 (strength* or weakness or fatigue or tonus)).tw,kf.	52213
22	Contracture/	8294
23	"Recovery of Function"/	56792
24	"Range of Motion, Articular"/	55499
25	locomotion/ or walking/ or gait/ or walking speed/ or stair climbing/	83838
26	"Activities of Daily Living"/ or (adl or (daily adj3 activit*)).tw,kf.	109732
27	Movement/	76933
28	muscle fatigue/ or muscle tonus/ or physical exertion/ or postural balance/ or Muscle Strength/	116716

© 2022 Olsen U et al. *JAMA Network Open.*

29	(sitting or lying or standing or balance or posture or rising or neeling or bend* or walk* or gait or stair* or extension* or stability or contracture* or movement* or motion* or locomotion* or mobility or twisting or pivoting or straighten* or swelling or grinding or clicking or squatting or running or jumping).tw,kf.	2041300
30	treatment outcome/ or treatment failure/ or outcome*.tw,kf.	2666783
31	patient reported outcome measures/	9627
32	("Knee injury and Osteoarthritis Outcome Score" or womac or koos or "American Knee Society Score" or AKSS or Kellgren Lawrence).tw,kf.	9722
33	or/20-32	7873792
34	cohort studies/ or follow-up studies/ or longitudinal studies/ or prospective studies/ or retrospective studies/	2219694
35	Postoperative Period/	54421
36	(post* or after or follow* or cohort* or prospectiv* or longitudinal).tw,kf.	9493302
37	or/34-36	10040161
38	and/11,33,37	6598
39	or/19,38	6890
40	limit 39 to yr="2000 -Current"	6577

© 2022 Olsen U et al. *JAMA Network Open.*

Embase Classic+Embase 1947 to 2021 October 04
Search date: 05.10.2021
Result of search: 6456

Search Strategy:

#	Searches	Results
1	knee replacement/ or total knee arthroplasty/	18303
2	(tkr or tjkr or tka or tjka).tw,kw.	18755
3	(knee adj3 (arthroplast* or replacement*)).tw,kw.	45317
4	(total adj2 knee*).tw,kw.	35030
5	(knee* adj2 prosthes*).tw,kw.	4009
6	or/1-5	52283
7	risk factor/ or risk/ or protection/ or risk assessment/	2124295
8	prognosis/ or (prognos* or risk* or predict*).tw,kw.	6181483
9	"prediction and forecasting"/ or prediction/	448064
10	(preoperative factor* or pre operative factor* or protective factor*).tw,kw.	33029
11	or/7-10	6686278
12	and/6,11	14777
13	(pain adj3 (post* or ongoing or on going or long* or persist* or prolong* or after or follow*)).tw,kw.	153914
14	postoperative pain/	75694
15	(pain/ or chronic pain/ or musculoskeletal pain/) and (post* or ongoing or on going or long* or persist* or prolonged or after or follow*).tw,kw.	242372
16	cohort analysis/ or follow up/ or longitudinal study/ or prospective study/ or retrospective study/	3602464
17	pain.tw,kw.	1050089
18	and/16-17	237320
19	or/13-15,18	516581
20	and/12,19	2853
21	knee function/ or muscle function/ or muscle rigidity/ or muscle contraction/ or muscle strength/ or muscle fatigue/ or muscle function/ or muscle stretching/ or muscle weakness/	245358
22	contracture/ or flexion contracture/ or joint contracture/ or muscle contracture/	22050
23	convalescence/	57391
24	locomotion/ or climbing/ or stair climbing/ or jumping/ or walking/ or gait/ or walking speed/	219096
25	daily life activity/ or (daily life activity or actvities of daily living or adl).tw,kw.	106658
26	exp musculoskeletal function/ or Movement/	1219923
27	joint swelling/ or grinding/	12441

© 2022 Olsen U et al. *JAMA Network Open.*

28	(function* or stiffness or contracture*).tw,kw.	5229428
29	(muscle adj3 (strength* or weakness or fatigue or tonus)).tw,kw.	76148
30	(sitting or lying or standing or balance or posture or rising or neeling or bend* or walk* or gait or stair* or extension* or stability or contracture* or movement* or motion* or locomotion* or mobility or twisting or pivoting or straighten* or swelling or grinding or clicking or squatting or running or jumping).tw,kw.	2586868
31	treatment outcome/ or treatment failure/ or patient-reported outcome/ or clinical outcome/ or outcome*.tw,kw.	3599641
32	"knee injury and osteoarthritis outcome score"/ or "Western Ontario and McMaster Universities Osteoarthritis Index"/ or ("Knee injury and Osteoarthritis Outcome Score" or womac or koos or "American Knee Society Score" or AKSS or Kellgren Lawrence).tw,kw.	17894
33	or/21-32	10631159
34	cohort analysis/ or follow up/ or longitudinal study/ or prospective study/ or retrospective study.mp. [mp=title, abstract, heading word, drug trade name, original title, device manufacturer, drug manufacturer, device trade name, keyword heading word, floating subheading word, candidate term word]	3636540
35	postoperative period/	234631
36	(post* or after or follow* or cohort* or prospectiv* or longitudinal).tw,kw.	13477914
37	or/34-36	14175462
38	and/12,33,37	8304
39	or/20,38	8832
40	limit 39 to yr="2000 -Current"	8546
41	limit 40 to conference abstract	2086
42	40 not 41	6456

CINAHL(Ebsco):
Search date: 05.10.2021
Result of search: 2412

#	Query	Limiters/Expanders	Results
S1	(MH "Arthroplasty, Replacement, Knee+")	Search modes - Boolean/Phrase	18,460
S2	TX tkr or tjkr or tka or tjka	Search modes - Boolean/Phrase	7,676
S3	TX knee* N3 (arthroplast* or replacement*)	Search modes - Boolean/Phrase	18,510
S4	TX (total N2 knee*)	Search modes - Boolean/Phrase	15,292

© 2022 Olsen U et al. *JAMA Network Open.*

S5	TX (knee* N2 prosthes*)	Search modes - Boolean/Phrase	874
S6	S1 OR S2 OR S3 OR S4 OR S5	Search modes - Boolean/Phrase	23,578
S7	(MH "Risk Factors")	Search modes - Boolean/Phrase	190,050
S8	(MH "Risk Assessment")	Search modes - Boolean/Phrase	120,213
S9	MH "Prognosis")	Search modes - Boolean/Phrase	88,876
S10	TX prognos* or risk* or predict* or preoperative factor* or protective factor*	Search modes - Boolean/Phrase	1,141,387
S11	S7 OR S8 OR S9 OR S10	Search modes - Boolean/Phrase	1910,505
S12	S6 AND S11	Search modes - Boolean/Phrase	6,153
S13	(TX pain N2 (TX (post* or ongoing or "on going" or long* or persist* or prolong* or after or follow*)) OR (MH "Postoperative Pain") OR TX pain AND (MH "Prospective Studies+")	Search modes - Boolean/Phrase	80,496
S14	(MH "Pain+") OR (MH "Knee Pain+") OR (MH "Muscle Pain") AND TX post* or ongoing or "on going" or long* or persist* or prolong* or after or follow*	Search modes - Boolean/Phrase	1,903,304
S15	S13 OR S14	Search modes - Boolean/Phrase	1,903,304
S16	S12 AND S15	Search modes - Boolean/Phrase	4,814
S17	(MH "Movement") OR (MH "Hopping") OR (MH "Jumping") OR (MH "Kneeling+") OR (MH "Extension+") OR (MH "Locomotion") OR (MH "Walking+") OR (MH "Gait+") OR (MH "Step") OR (MH "Range of Motion") OR (MH "Rising") OR (MH "Sitting") OR (MH "Squatting") OR (MH "Stair Climbing") OR (MH "Standing+") OR (MH "Stretching")	Search modes - Boolean/Phrase	92,949
S18	(MH "Muscle Fatigue") OR (MH "Muscle Strength+") OR (MH "Muscle Tonus")	Search modes - Boolean/Phrase	30,937
S19	TX (function* or stiffness or contracture*)	Search modes - Boolean/Phrase	500,860

© 2022 Olsen U et al. *JAMA Network Open.*

S20	TX (muscle N3 (strength* or weakness or fatigue or tonus))	Search modes - Boolean/Phrase	21,279
S21	(MH "Contracture+")	Search modes - Boolean/Phrase	2,204
S22	(MH "Activities of Daily Living+")	Search modes - Boolean/Phrase	75,269
S23	TX (actvities or daily living or adl)	Search modes - Boolean/Phrase	46,034
S24	(MH "Treatment Outcomes+") OR (MH "Fatal Outcome") OR (MH "Treatment Failure")	Search modes - Boolean/Phrase	402,301
S25	TX outcome*	Search modes - Boolean/Phrase	721,791
S26	TX "Knee injury and Osteoarthritis Outcome Score" or womac or koos or "American Knee Society Score" or AKSS or Kellgren Lawrence)	Search modes - Boolean/Phrase	11,338
S27	S17 OR S18 OR S19 OR S20 OR S21 OR S22 OR S23 OR S24 OR S25 OR S26	Search modes - Boolean/Phrase	1,470,810
S28	(MH "Postoperative Period") OR (MH "Prospective Studies+")	Search modes - Boolean/Phrase	491,778
S29	TX (post* or after or follow* or cohort* or prospectiv* or longitudinal)	Search modes - Boolean/Phrase	1,845,119
S30	S28 OR S29	Search modes - Boolean/Phrase	1,966,707
S31	S12 AND S27 AND S30	Search modes - Boolean/Phrase	3,363
S32	S16 OR S31<br>**Limiters** - Published Date: 20000101-20211031	Search modes - Boolean/Phrase	5,059

The Cochrane Library
Search date: 05.10.2021

© 2022 Olsen U et al. *JAMA Network Open.*

Result of search: 1127 Cochrane Reviews (21) Trials (1106)

#1	MeSH descriptor: [Arthroplasty, Replacement, Knee] explode all trees	2721
#2	(tkr or tjkr or tka or tjka):ti,ab,kw	3715
#3	(knee near/3 (arthroplast* or replacement*)):ti,ab,kw	8696
#4	(total near/2 knee*):ti,ab,kw	7164
#5	(knee near/2 prosthes*):ti,ab,kw	1284
#6	#1 or #2 or #3 or #4 or #5	9242
#7	MeSH descriptor: [Risk] explode all trees	38325
#8	MeSH descriptor: [Prognosis] this term only	14053
#9	(prognos* or risk* or predict*):ti,ab,kw	354478
#10	((preoperative or "pre operative" or protective) near/2 factor*):ti,ab,kw	1482
#11	#7 or #8 or #9 or #10	356939
#12	#6 and #11	1585
#13	(pain near/3 (post* or ongoing or "on going" or long* or persist* or prolong* or after or follow*)):ti,ab,kw	56869
#14	MeSH descriptor: [Pain, Postoperative] explode all trees	16393
#15	MeSH descriptor: [Pain] this term only	11960
#16	MeSH descriptor: [Chronic Pain] this term only	2790
#17	MeSH descriptor: [Musculoskeletal Pain] this term only	531
#18	#14 or #15 or #16 or #17	31200
#19	(post* or ongoing or on going or long* or persist* or prolonged or after or follow*):ti,ab,kw	1143252
#20	#18 and #19	26936
#21	MeSH descriptor: [Cohort Studies] explode all trees	154431
#22	(pain):ti,ab,kw	195141
#23	#21 and #22	21911
#24	#13 or #20 or #23	75837
#25	#12 and #24	420
#26	(function* or stiffness or contracture*):ti,ab,kw	286360
#27	(muscle near/3 (strength* or weakness or fatigue or tonus)):ti,ab,kw	21991
#28	MeSH descriptor: [Contracture] this term only	188
#29	MeSH descriptor: [Recovery of Function] this term only	5518
#30	MeSH descriptor: [Range of Motion, Articular] this term only	5055
#31	MeSH descriptor: [Locomotion] explode all trees	8647
#32	MeSH descriptor: [Walking] explode all trees	5891
#33	MeSH descriptor: [Activities of Daily Living] this term only	5124
#34	("actvities of daily living" or adl):ti,ab,kw	3712
#35	MeSH descriptor: [Movement] this term only	2471
#36	MeSH descriptor: [Muscle Fatigue] this term only	1022
#37	MeSH descriptor: [Muscle Tonus] this term only	292
#38	MeSH descriptor: [Physical Exertion] explode all trees	3931
#39	MeSH descriptor: [Postural Balance] this term only	2960

© 2022 Olsen U et al. *JAMA Network Open.*

#40	MeSH descriptor: [Muscle Strength] this term only	4843
#41	(sitting or lying or standing or balance or posture or rising or neeling or bend* or walk* or gait or stair* or extension* or stability or contracture* or movement* or motion* or locomotion* or mobility or twisting or pivoting or straighten* or swelling or grinding or clicking or squatting or running or jumping):ti,ab,kw	173425
#42	MeSH descriptor: [Treatment Outcome] this term only	139508
#43	MeSH descriptor: [Treatment Failure] this term only	3374
#44	(outcome):ti,ab,kw	536106
#45	MeSH descriptor: [Patient Reported Outcome Measures] this term only	811
#46	("Knee injury and Osteoarthritis Outcome Score" or womac or koos or "American Knee Society Score" or AKSS or "Kellgren Lawrence"):ti,ab,kw	6098
#47	#26 or #27 or #28 or #29 or #30 or #31 or #32 or #33 or #34 or #35 or #36 or #37 or #38 or #39 or #40 or #41 or #42 or #43 or #44 or #45 or #46	791259
#48	MeSH descriptor: [Cohort Studies] explode all trees	154431
#49	MeSH descriptor: [Postoperative Period] explode all trees	6137
#50	(post* or after or follow* or cohort* or prospectiv* or longitudinal):ti,ab,kw	1150202
#51	#48 or #49 or #50	1151727
#52	#47 and #51	604333
#53	#12 and #52	1089
#54	#25 or #53 with Cochrane Library publication date Between Jan 2000 and Oct 2021	1127

PRDro (Physiotherapy Evidence Database):

Search date: 05.10.2021
Result of search: Søk 1: 120 Søk 2: 13

Søk 1: Abstract & Title : total knee* replacement*, Body Part: lower leg or knee, Published since:2000
Søk 2: Abstract & Title:, knee* prosthes*, Body Part: lower leg or knee, Published since: 2000

© 2022 Olsen U et al. *JAMA Network Open.*

eTable 6. Reason for Exclusion of Individual Studies

Authors and year	Reason for exclusion*
(Abane et al., 2015)	1. No regression performed
(Abdel et al., 2014)	1. No regression performed
(Aderinto et al., 2005)	5. Not separate analysis for OA
(Adie et al., 2012)	1. No regression performed
(Ahmed et al., 2009)	1. No regression performed
(Alentorn-Geli et al., 2013)	1. No regression performed
(Alomran, 2015)	1. No regression performed
(Amusat et al., 2014)	7. Insufficient information about the sample**
(Andrawis et al., 2015)	5. Not separate analysis for TKA
(Arden et al., 2017)	3. Inadequate study design
(Arendt-Nielsen et al., 2018)	4. Predictor not evaluated
(Arendt-Nielsen et al., 2018)	2. Duplicate publication
(Aso et al., 2021)	5. Not separate analysis for TKA
(Attal et al., 2014)	4. Outcome not evaluated
(Ayers et al., 2005)	5. Not separate analysis for OA
(Ayers et al., 2013)	5. Not separate analysis for OA
(Bade et al., 2012)	5. Not separate analysis intervention & control
(Bade et al., 2014)	5. Not separate analysis intervention & control
(Barrack et al., 2014)	3. Inadequate study design
(Barroso et al., 2020)	3. Inadequate aim
(Bascuas et al., 2013)	1. No regression performed for the outcome
(Bauer et al., 2010)	6. Insufficient follow-up time
(Behrend et al., 2019)	3. Inadequate study design
(Belford et al., 2020)	7. Insufficient data about the sample**
(Berghmans et al., 2015)	2. Conference abstract
(Bergschmidt et al., 2008)	1. No regression performed
(Bethge et al., 2010)	1. No regression performed
(Bian et al., 2021)	3. Inadequate aim
(Bierke and Petersen, 2017)	1. No regression performed
(Bin and Nam, 2007)	1. No regression performed
(Bistolfi et al., 2017)	1. No regression performed
(Blackburn et al., 2012)	1. No regression performed
(Boerger et al., 2005)	1. No regression performed
(Bossmann et al., 2017)	4. Outcome not evaluated
(Bove et al., 2022)	5. Not separate measure of function
(Braaksma et al., 2020)	6. Insufficient follow-up time
(Brander et al., 2003)	5. Not separate reporting for the OA population
(Brock et al., 2017)	5. Not separate reporting for the OA population
(Brockenbrough, 2007)	7. Insufficient data about the sample**
(Browne, 2013)	2. Commentary
(Brummett et al., 2011)	2. Conference abstract
(Brummett et al., 2013)	2. Conference abstract
(Brummett et al., 2015)	5. Not separate reporting for TKA population
(Bumberger et al., 2021)	3. Inadequate study design
(Buvanendran et al., 2011)	2. Conference abstract
(Buvanendran et al., 2012)	2. Conference abstract
(Caracciolo and Giaquinto, 2005)	3. Inadequate aim
(Carvalho Junior et al., 2017)	1. No regression performed
(Chalidis et al., 2010)	1. No regression performed for the outcome
(Chang et al., 2010)	5. Not separate analysis for TKA

© 2022 Olsen U et al. *JAMA Network Open.*

(Chen et al., 2021)	3. Inadequate aim
(Cheng et al., 2010)	3. Inadequate study design
(Cheuy et al., 2019)	1. No regression performed for the outcome
(Chouteau et al., 2009)	3. Ineligible study design
(Chowdhry et al., 2014)	3. Ineligible study design
(Christensen et al., 2019)	7. Insufficient data**
(Christensen et al., 2021)	3. Inadequate aim
(Clement et al., 2013a)	4. Outcome not evaluated
(Clement et al., 2013b)	5. Not separate data for function
(Clement et al., 2011)	1. No regression performed for the outcome
(Clement et al., 2013c)	5. Not separate data for function
(Collins et al., 2017)	5. Not separate analysis intervention & control
(Collins et al., 2016)	2. Conference abstract
(Cooper et al., 2017)	5. Not separate analysis intervention & control
(Cornelius et al., 2017)	2. Conference abstract
(Cornelius et al., 2015)	2. Conference abstract
(Cremeans-Smith et al., 2012)	2. Conference abstract
(Cremeans-Smith et al., 2015a)	4. Outcome not evaluated
(Cremeans-Smith et al., 2013)	2. Conference abstract
(Cremeans-Smith et al., 2015b)	4. Outcome not evaluated
(Cremeans-Smith et al., 2018)	2. Conference abstract
(Cremeans-Smith et al., 2016)	4. Outcome not evaluated
(Dailiana et al., 2015)	2. Duplicate
(Dalury et al., 2009)	1. No regression performed for the outcome
(Dave et al., 2017)	3. Insufficient aim
(Davis et al., 2009)	2. Conference abstract
(Davis et al., 2017)	2. Conference abstract
(Dere et al., 2014)	1. No regression performed for the outcome
(Desmeules et al., 2013)	5. Not separate analysis for OA
(Dierick et al., 2004)	1. No regression performed for the outcome
(Djadoun et al., 2014)	2. Conference abstract
(Dossett et al., 2012)	1. No regression performed for the outcome
(Dowsey et al., 2009)	5. Not separate analysis for OA
(Dowsey et al., 2014)	5. Not separate analysis for OA
(Dowsey et al., 2015)	5. Not separate analysis for OA
(Dowsey et al., 2016)	5. Not separate analysis for OA
(Doury-Panchout et al., 2015)	3. Ineligible study design
(Duivenvoorden et al., 2013)	5. Not separate analysis for OA
(Dumenci et al., 2019)	5. Not separate analysis for OA
(Dursteler et al., 2021)	1. No adequate regression performed
(Dutka et al., 2011)	5. Not separate analysis for OA
(Dutton et al., 2008)	1. No regression performed for the outcome
(Edwards et al., 2009)	4. Outcome not evaluated
(Ellis et al., 2012)	1. No regression performed for the outcome
(Faller et al., 2003)	2. Letter to editor
(Farahini et al., 2012)	5. Not separate analysis for OA
(Farin et al., 2006)	5. Not separate analysis for TKA
(Fernandez-Fairen et al., 2013)	1. No regression performed for the outcome
(Ferreira et al., 2021)	1. No adequate regression performed
(Ferrer et al., 2020)	3. Inadequate aim
(Filardo et al., 2017)	5. Not separate data for function, used a total score
(Filbay and Judge, 2017)	2. Conference
(Filbay et al., 2018)	5. Not separate reporting of function

(Fitzpatrick et al., 2017)	2. Conference
(Fitzsimmons et al., 2018)	4. Outcome not evaluated
(Fitzsimmons et al., 2018)	2. Duplicate publication
(Fleeton et al., 2016)	5. Allocation groups were pooled
(Foran et al., 2004)	6. Insufficient follow-up time
(Forsythe et al., 2008)	1. No regression performed
(Franklin et al., 2008)	5. Not separate analysis for OA
(Franklin et al., 2013)	2. Conference
(Furu et al., 2016)	5. Not separate analysis for OA
(Gandhi et al., 2009a)	5. Not separate analysis for TKA
(Gandhi et al., 2010a)	5. Not separate analysis for TKA
(Gandhi et al., 2010b)	2. Duplicate publication
(Gandhi et al., 2010c)	5. Not separate analysis for TKA
(Gandhi et al., 2009b)	5. Not separate analysis for TKA
(Gandhi et al., 2009c)	5. Not separate analysis for TKA
(Gandhi et al., 2009d)	5. Not separate analysis for TKA
(Gates et al., 2016)	2. Conference
(Gates et al., 2017)	4. Outcome not evaluated
(Gatha et al., 2004)	6. Insufficient follow-up time
(Getachew et al., 2021)	3. Inadequate aim
(Giesinger et al., 2016)	2. Conference
(Giordano et al., 2020)	1. No adequate regression performed
(Giordano et al., 2021)	3. Inadequate aim
(Gonzalez Saenz de Tejada et al., 2014)	5. Not separate analysis for TKA
(Graves et al., 2014)	1. No regression performed
(Gray et al., 2017)	5. Used the total score of WOMAC
(Greco et al., 2017)	2. Conference abstract
(Greenidge et al., 2009)	2. Conference abstract
(Grosu et al., 2013)	2. Conference abstract
(Group et al., 2009)	1. No regression performed
(Guimaraes-Pereira et al., 2016)	5. Not separate analysis for TKA
(Gøthesen et al., 2014)	5. Not separate results for OA
(Ha and Ha, 2006)	7. Insufficient information about age
(Halket et al., 2010)	5. Not separate analysis for TKA
(Hamilton et al., 2015)	1. No regression performed
(Hamilton et al., 2017)	2. Conference abstract
(Hamilton et al., 2021)	5. Not separate measure of function
(Hanratty et al., 2011)	1. No regression performed
(Hanusch et al., 2014)	7. Insufficient information follow-up time**
(Harden et al., 2003)	4. Outcome not evaluated
(Hasegawa et al., 2021)	3. Inadequate aim
(Hashimoto et al., 2019)	1. No regression performed
(Hemert et al., 2011)	1. No regression performed
(Hinarejos et al., 2016)	1. No regression performed
(Hirschmann et al., 2010)	1. No regression performed
(Hirschmann et al., 2013)	1. No regression performed
(Hitt et al., 2015)	1. No regression performed
(Hodges et al., 2018)	5. Outcome not evaluated
(Hodges et al., 2018)	2. Duplicate publication
(Hommel et al., 2017)	1. No regression performed.
(Hofstede et al., 2018)	3. Inadequate study design
(Hofstede et al., 2018)	2. Duplicate publication
(Hooper et al., 2012)	1. No regression performed

© 2022 Olsen U et al. *JAMA Network Open.*

(Hylkema et al., 2019)	2. Duplicate
(Hourlier and Fennema, 2014)	1. No regression performed
(Hovik et al., 2016)	1. No regression performed
(Hughes et al., 2018)	2. Conference abstract
(Haanstra et al., 2015)	3. Inadequate aim
(Ingleshwar et al., 2013)	2. Conference abstract
(Jacobs et al., 2016a)	2. Conference abstract
(Jacobs et al., 2016b)	2. Conference abstract
(Jain et al., 2017)	7. Insufficient information**
(Jamsen et al., 2015)	2. Conference abstract
(Jarvenpaa et al., 2010a)	1. No regression performed
(Jarvenpaa et al., 2010b)	2. Duplicate
(Jefferies et al., 2012)	3. Study design
(Jiang et al., 2017)	5. Not separate analysis for OA
(Jolles et al., 2012)	5. Insufficient aim
(Jonbergen et al., 2011)	1. No regression performed
(Jones et al., 2012a)	6. Insufficient follow-up time
(Jones et al., 2003)	5. Not separate analysis for OA
(Jones et al., 2012b)	2. Conference abstract
(Judge et al., 2012)	7. Insufficient age of participant (<18 years)
(Judge et al., 2010)	2. Conference abstract
(Julie et al., 2013)	2. Conference abstract
(Kahlenberg et al., 2018)	1. No regression performed
(Kang et al., 2010)	3. Inadequate study design
(Katakam et al., 2021)	3. Inadequate study design
(Katz et al., 2011)	2. Conference abstract
(Keeney et al., 2017)	3. Inadequate aim
(Kelly et al., 2006)	3. Inadequate aim
(Kennedy et al., 2008)	3. Inadequate aim
(Khanna, 2016)	2. Conference abstract
(Kilicarslan et al., 2011)	1. No regression performed
(Kim et al., 2015)	5. Not separate analysis for TKA
(Kim et al., 2009)	1. No regression performed
(Ko et al., 2010)	2. Conference abstract
(Kornilov et al., 2018)	3. Inadequate aim
(Kurien et al., 2018)	3. Inadequate aim
(Kurien et al., 2018)	2. Duplicate publication
(Lam et al., 2003)	1. No regression performed
(Lamb and Frost, 2003)	1. No regression performed
(Lampe et al., 2016)	5. Pooled data intervention & control
(Lange et al., 2016)	2. Conference abstract
(Larsen et al., 2021)	5. Pooled data intervention & control
(Laskow et al., 2021)	5. Not separate analysis for OA
(Lebleu et al., 2019)	5. Not separate analysis for TKA
(Ledin et al., 2012)	1. No regression performed
(Lee et al., 2015)	5. Not separate analysis for TKA
(Leung et al., 2017)	2. Conference abstract
(Leung et al., 2019)	5. Not separate analysis for TKA
(Li et al., 2013)	2. Conference abstract
(Liebs et al., 2011)	5. Not separate analysis for TKA
(Lindberg et al., 2016)	1. No regression performed
(Lindner et al., 2018)	2. Duplicate
(Lingard et al., 2004)	1. Unknown if regression is performed**

© 2022 Olsen U et al. *JAMA Network Open.*

(Liu et al., 2020)	1. No regression performed
(Lizaur-Utrilla et al., 2012)	3. Inadequate aim
(Lungu et al., 2014)	1. No regression performed
(Lustig et al., 2012)	5. Pooled data, more knees than patients
(Lutzner et al., 2014)	1. No regression performed
(Lützner et al., 2014)	1. No regression performed
(Macaulay et al., 2010)	2. Conference abstract
(Maculé et al., 2005)	1. No regression performed
(Maffulli et al., 2011)	3. Inadequate study design
(Magaldi et al., 2019)	3. Inadequate aim
(Mahomed et al., 2002)	5. Not separate analysis for TKA
(Mahoney et al., 2012)	1. No regression performed
(Malviya et al., 2009)	1. No regression performed
(Martinez et al., 2007)	1. No regression performed
(Mat et al., 2016)	3. Inadequate study design and aim
(Maus et al., 2017)	1. No regression performed
(Maxwell et al., 2013)	6. Insufficient follow-up time
(Meessen et al., 2018)	1. No regression performed
(Mehta and Lotke, 2007)	1. No regression performed
(Mehta et al., 2014)	2. Conference abstract
(Mehta et al., 2015)	3. Inadequate study design
(Meijerink et al., 2009)	6. Insufficient follow-up time
(Mercurio et al., 2020)	5. Not separate analysis for TKA
(Metsna et al., 2014)	1. No regression performed
(Miozzari et al., 2013)	2. Conference abstract
(Mittal et al., 2012)	1. No regression performed
(Mizner et al., 2005)	3. Inadequate aim
(Moghtadaei et al., 2020)	3. Inadequate study design
(Molt and Toksvig-Larsen, 2014)	1. No regression performed
(Morze et al., 2013)	1. No regression performed
(Motwani et al., 2013)	3. Inadequate study design
(Nandi et al., 2016)	2. Conference abstract
(Nankaku et al., 2018)	2. Duplicate publication
(Navarro Collado et al., 2000)	5. Not separate analysis for OA
(Naylor et al., 2008)	5. Not separate results for OA population
(Neogi et al., 2010)	2. Conference abstract
(Neuburger et al., 2013)	5. Not separate results for OA population
Neuprez et al., 2018)	5. Not separate results for TKA
(Neuprez et al., 2018)	2. Duplicate publication
(Nielsen et al., 2018)	6. Insufficient follow-up time
(Nielsen et al., 2018)	2. Duplicate publication
(Niki et al., 2015)	6. Insufficient follow-up time
(Noiseux et al., 2014)	5. Pooled data from intervention & control
(Nuñez et al., 2011)	3. Inadequate study design
(Nwankwo et al., 2021)	5. Not separate results for primary TKA
(Oatis et al., 2012)	2. Conference abstract
(Oberbek and Synder, 2015)	1. No regression performed
(Okamoto et al., 2014)	5. Pooled data, more knees than patients
(Otero et al., 2016)	6. Insufficient follow-up time.
(Ozdemir et al., 2017)	1. No regression performed
(Page et al., 2014)	2. Conference abstract
(Page et al., 2015)	1. No regression performed
(Pan et al., 2019)	3. Inadequate study design and aim

© 2022 Olsen U et al. *JAMA Network Open.*

(Papakostidou et al., 2012)	7. Insufficient data on analysis**
(Parsley et al., 2010)	1. No regression performed
(Paxton et al., 2016)	6. Insufficient follow-up time
(Pereira et al., 2016)	5. Not separate results for TKA
(Perruccio et al., 2010a)	5. Not separate results for TKA
(Perruccio et al., 2010b)	2. Duplicate publication
(Perruccio et al., 2011a)	1. No regression performed
(Perruccio et al., 2011b)	2. Conference abstract
(Perruccio et al., 2019)	4. Outcome not evaluated
(Petersen et al., 2015)	3. Inadequate aim
(Petersen et al., 2017)	3. Inadequate aim
{Petersen, 2018 #897}	2. Duplicate publication
(Petersen et al., 2020)	3. Inadequate aim
(Pinsornsak et al., 2014)	1. No regression performed
(Pinto et al., 2013)	5. Not separate results for TKA
(Pinto et al., 2014)	2. Conference abstract
(Polkowski et al., 2013)	6. Insufficient follow-up time
(Pont et al., 2011)	1. No regression performed
(Pua et al., 2012)	2. Conference abstract
(Pua et al., 2017)	4. Prognostic factor not evaluated
(Pua et al., 2013)	4. Prognostic factor not evaluated
(Pua et al., 2015)	1. No regression performed
(Yong-Hao et al., 2016)	2. Same sample as in a later included study
(Quintana et al., 2006)	5. Not separate results for TKA
(Radmer et al., 2006)	5. Not separate results for OA
(Rajamaki et al., 2015)	5. Not separate results for OA
(Rakel et al., 2013)	2. Conference abstract
(Ramaesh et al., 2014)	5. Not separate results for OA
(Razmjou et al., 2015)	6. Insufficient follow-up time
(Reid et al., 2014)	3. Inadequate aim
(Richards et al., 2016)	2. Conference abstract
(Riddle et al., 2009)	2. Conference abstract
(Riddle et al., 2015)	1. No regression performed
(Riddle, 2018)	5. Not separate results for TKA
(Riddle et al., 2020)	5. Pooled results from RCT
(Rice et al., 2018)	5. Not separate results for OA
(Rosen et al., 2013)	3. Inadequate aim
(Russell et al., 2014)	1. No regression performed
(Sakellariou et al., 2016)	2. Conference abstract
(Salazar et al., 2013)	2. Conference abstract
(Sanchez-Santos et al., 2014)	2. Conference abstract
(Sanchez-Santos et al., 2018)	5. Not separate results for OA
(Schaumburger et al., 2012)	1. No regression performed
(Schwartz et al., 2012)	4. Prognostic factor not evaluated
(Scott et al., 2010)	4. Outcome not evaluated
(Scott et al., 2012)	4. Outcome not evaluated
(Seol et al., 2016)	3. Inadequate study design
(Sharma et al., 2021)	7. Insufficient data on population**
(Shim et al., 2018)	7. Age < 18 years
(Singh et al., 2015)	3. Inadequate study design
(Siviero et al., 2020)	5. Not separate results for TKA
(Slevin et al., 2017)	1. No regression performed
(Smith et al., 2006)	6. Insufficient follow-up time

© 2022 Olsen U et al. *JAMA Network Open.*

(Smith et al., 2014)	5. Not separate results for TKA
(Smith et al., 2012)	1. No regression performed
(Smith et al., 2019)	6. Insufficient follow-up time
(Smith et al., 2019)	6. Insufficient follow-up time/duplicate
(Soni et al., 2014)	2. Conference abstract
(Soni et al., 2016)	2. Conference abstract
(Stickles et al., 2001)	1. No regression performed
(Stone et al., 2017)	3. Inadequate study design
(Stratford et al., 2010)	6. Insufficient follow-up time
(Street et al., 2018)	3. Inadequate study design
(Sveikata et al., 2017)	1. No regression performed
(Tabutin et al., 2005)	1. No regression performed
(Tan et al., 2014)	5. Pooled results, more knees than patients
(Tchetina et al., 2020)	1. No regression performed
(Thomazeau et al., 2016)	5. Not separate results for TKA
(Tilbury et al., 2016)	6. Insufficient data
(Tilbury et al., 2018)	2. Duplicate publication
(Toguchi et al., 2020)	3. Inadequate study design
(Tolk et al., 2021)	1. No regression reported on outcome
(Trace, 2006)	3. Inadequate study design
(Twiggs et al., 2019)	3. Inadequate aim
(Utrillas-Compaired et al., 2014)	5. Not separate results for OA
(Vaegter et al., 2017)	1. No regression reported
(van den Akker-Scheek et al., 2007)	5. Not separate results for TKA
(Van Hamersveld et al., 2018)	4. Inadequate outcome
(Van Hamersveld et al., 2018)	4. Inadequate outcome
(Van Hamersveld et al., 2018)	2. Duplicate
(van Loon et al., 2021)	6. Insufficient follow-up time
(Van Onsem et al., 2018)	4. Inadequate outcome
(Vekama et al., 2015)	5. Not separate analysis for TKA
(Vela et al., 2017)	2. Conference abstract
(Vila et al., 2020)	5. Not separate analysis for TKA
(Vina et al., 2014)	2. Conference abstract
(Vina et al., 2016)	6. Insufficient follow-up time
(Vogel et al., 2019)	7. Insufficient data about eligibility
(Wada et al., 2016)	1. No regression reported
(Walker et al., 2015)	2. Conference abstract
(Wenjun et al., 2017)	3. Wrong aim
(Widmer et al., 2013)	5. Not separate analysis for TKA
(Williams et al., 2013)	5. Not separate analysis for TKA
(Winters et al., 2014)	3. Inadequate study design
(Wohlrab et al., 2005)	1. No regression reported
(Wollmerstedt et al., 2006)	5. Not separate analysis for TKA
(Woo et al., 2006)	6. Data from article was unavailable
(Wood et al., 2021)	5. Not separate analysis for TKA
(Wright et al., 2017)	5. Not separate analysis for TKA
(Wylde et al., 2013)	1. No regression performed for the outcome
(Wylde et al., 2015)	5. Pooled results intervention & control
(Wylde et al., 2017)	2. Same sample as in prior included study
(Xu et al., 2020)	5. Not separate measure of function
(Yakobov et al., 2018)	4. Inadequate outcome
(Yap et al., 2021)	5. Not separate measure of function
(Yau et al., 2005)	5. Not separate results for OA

© 2022 Olsen U et al. *JAMA Network Open.*

(Young et al., 2017)	3. Inadequate aim
(Young-Shand et al., 2020)	3. Inadequate aim
(Zeni and Snyder-Mackler, 2010)	5. Pooled results intervention & control

Abbreviations: OA; osteoarthritis, RA; rheumatoid arthrithis, TKA; total knee arthroplasty. THA; total hip arthroplasty
*Reason for exclusion correspond with the PRISMA flow diagram
**Author did not respond to e-mail or gave insufficient information about the study

**No of studies**	**Reason for exclusion**
90	1. No regression performed
80	2. Conference abstract, duplicate publication or letter to editor
54	3. Inadequate study design or aim
21	4. Predictor or outcome not evaluated
94	5. TKA/OA/pooled results/total score
20	6. Insufficient follow-up time
12	7. Insufficient data or age>18 years

References

ABANE, L., ANRACT, P., BOISGARD, S., DESCAMPS, S., COURPIED, J. & HAMADOUCHE, M. 2015. A comparison of patient-specific and conventional instrumentation for total knee arthroplasty: a multicentre randomised controlled trial. *The bone & joint journal,* 97**,** 56-63.

ABDEL, M., PARRATTE, S., BLANC, G., OLLIVIER, M., POMERO, V., VIEHWEGER, E. & ARGENSON, J. 2014. No benefit of patient-specific instrumentation in TKA on functional and gait outcomes: a randomized clinical trial.

ADERINTO, J., BRENKEL, I. J. & CHAN, P. 2005. Natural history of fixed flexion deformity following total knee replacement: a prospective five-year study. *Journal of Bone & Joint Surgery - British Volume,* 87**,** 934-6.

ADIE, S., DAO, A., HARRIS, I. A., NAYLOR, J. M. & MITTAL, R. 2012. Satisfaction with joint replacement in public versus private hospitals: a cohort study. *ANZ Journal of Surgery,* 82**,** 616-24.

AHMED, I., GRAY, A., LINDEN, M. & NUTTON, R. 2009. Range of flexion after primary TKA: the effect of soft tissue release and implant design. *Orthopedics,* 32**,** 811.

ALENTORN-GELI, E., LEAL-BLANQUET, J., GUIRRO, P., HINAREJOS, P., PELFORT, X. & PUIG-VERDIÉ, L. 2013. Comparison of Quality of Life Between Elderly Patients Undergoing TKA. *Orthopedics,* 36**,** 253-253.

ALOMRAN, A. 2015. Effect of patellar denervation on mid-term results after non-resurfaced total knee arthroplasty. A randomised, controlled trial. *Acta orthopaedica belgica,* 81**,** 609-613.

AMUSAT, N., BEAUPRE, L., JHANGRI, G. S., POHAR, S. L., SIMPSON, S., WARREN, S. & JONES, C. A. 2014. Diabetes that impacts on routine activities predicts slower recovery after total knee arthroplasty: an observational study. *Journal of Physiotherapy,* 60**,** 217-23.

ANDRAWIS, J., AKHAVAN, S., CHAN, V., LEHIL, M., PONG, D. & BOZIC, K. J. 2015. Higher Preoperative Patient Activation Associated With Better Patient-reported Outcomes After Total Joint Arthroplasty. *Clinical Orthopaedics & Related Research,* 473**,** 2688-97.

ARDEN, N., ALTMAN, D., BEARD, D., CARR, A., CLARKE, N., COLLINS, G., COOPER, C., CULLIFORD, D., DELMESTRI, A., GARDEN, S., GRIFFIN, T., JAVAID, K., JUDGE, A., LATHAM, J., MULLEE, M., MURRAY, D., OGUNDIMU, E., PINEDO-VILLANUEVA, R., PRICE, A., PRIETO-ALHAMBRA, D. & RAFTERY, J. 2017. *NIHR Journals Library. Programme Grants for Applied Research,* 6**,** 06.

ARENDT-NIELSEN, L., SIMONSEN, O., LAURSEN, M. B., ROOS, E. M., RATHLEFF, M. S., RASMUSSEN, S. & SKOU, S. T. 2018. Pain and sensitization after total knee replacement or nonsurgical treatment in patients with knee osteoarthritis: Identifying potential predictors of outcome at 12 months. *European Journal of Pain,* 22**,** 1088-1102.

ASO, K., IKEUCHI, M., TAKAYA, S., SUGIMURA, N., IZUMI, M., WADA, H., OKANOUE, Y. & DAN, J. 2021. Chronic postsurgical pain after total knee arthroplasty: A prospective cohort study in Japanese population. *Modern Rheumatology,* 31**,** 1038-1044.

ATTAL, N., MASSELIN-DUBOIS, A., MARTINEZ, V., JAYR, C., ALBI, A., FERMANIAN, J., BOUHASSIRA, D. & BAUDIC, S. 2014. Does cognitive functioning predict chronic pain? Results from a prospective surgical cohort. *Brain,* 137**,** 904-917.

AYERS, D. C., FRANKLIN, P. D., PLOUTZ-SNYDER, R. & BOISVERT, C. B. 2005. Total knee replacement outcome and coexisting physical and emotional illness. *Clinical Orthopaedics & Related Research,* 440**,** 157-61.

AYERS, D. C., LI, W., OATIS, C., ROSAL, M. C. & FRANKLIN, P. D. 2013. Patient-reported outcomes after total knee replacement vary on the basis of preoperative coexisting disease in the lumbar spine and other nonoperatively treated joints,the need for a musculoskeletal comorbidity index. *Journal of Bone and Joint Surgery - Series A,* 95**,** 1833-1837.

BADE, M., KITTELSON, J., KOHRT, W. & STEVENS-LAPSLEY, J. 2014. Predicting functional performance and range of motion outcomes after total knee arthroplasty. *American journal of physical medicine & rehabilitation,* 93**,** 579-585.

BADE, M. J., WOLFE, P., ZENI, J. A., STEVENS-LAPSLEY, J. E. & SNYDER-MACKLER, L. 2012. Predicting poor physical performance after total knee arthroplasty. *Journal of Orthopaedic Research,* 30**,** 1805-10.

BARRACK, R. L., RUH, E. L., CHEN, J., LOMBARDI JR, A. V., BEREND, K. R., PARVIZI, J., DELLA VALLE, C. J., HAMILTON, W. G. & NUNLEY, R. M. 2014. Impact of socioeconomic factors on outcome of total knee arthroplasty knee. *Clinical Orthopaedics and Related Research,* 472**,** 86-97.

BARROSO, J., WAKAIZUMI, K., RECKZIEGEL, D., PINTO-RAMOS, J., SCHNITZER, T., GALHARDO, V. & APKARIAN, A. V. 2020. Prognostics for pain in osteoarthritis: Do clinical measures predict pain after total joint replacement? *PLoS One,* 15**,** e0222370.

BASCUAS, I., TEJERO, M., MONLEON, S., BOZA, R., MUNIESA, J. M. & BELMONTE, R. 2013. Balance 1 year after TKA: correlation with clinical variables. *Orthopedics,* 36**,** e6-12.

BAUER, T., BIAU, D., COLMAR, M., POUX, X., HARDY, P. & LORTAT-JACOB, A. 2010. Influence of posterior condylar offset on knee flexion after cruciate-sacrificing mobile-bearing total knee replacement: a prospective analysis of 410 consecutive cases. *Knee,* 17**,** 375-80.

BEHREND, H., GRAULICH, T., GERLACH, R., SPROSS, C. & LADURNER, A. 2019. Blackburne-Peel ratio predicts patients' outcomes after total knee arthroplasty. *Knee Surgery, Sports Traumatology, Arthroscopy,* 27**,** 1562-1569.

BELFORD, K., GALLAGHER, N., DEMPSTER, M., WOLFENDEN, M., HILL, J., BLANEY, J., O'BRIEN, S., SMIT, A. M., BOTHA, P., MOLLOY, D. & BEVERLAND, D. 2020. Psychosocial predictors of outcomes up to one year following total knee arthroplasty. *Knee,* 27**,** 1028-1034.

BERGHMANS, D., LENSSEN, A., DE BIE, R. A. & VAN RHIJN, L. 2015. Functioning with knee osteoarthritis and one year after a TKA: Are we able to predict functional recovery? *Physiotherapy (United Kingdom),* 1**,** eS140-eS141.

BERGSCHMIDT, P., BADER, R., FINZE, S., ANSORGE, S., KUNDT, G. & MITTELMEIER, W. 2008. [Bicondylar knee arthroplasty - influence of preoperative functional restriction on early functional postoperative outcome]. *Zeitschrift fur Orthopadie & Unfallchirurgie,* 146**,** 344-51.

BETHGE, M., BARTEL, S., STREIBELT, M., LASSAHN, C. & THREN, K. 2010. [Illness perceptions and functioning following total knee and hip arthroplasty]. *Zeitschrift fur Orthopadie & Unfallchirurgie,* 148**,** 387-92.

BIAN, T., SHAO, H., ZHOU, Y., HUANG, Y. & SONG, Y. 2021. Does psychological distress influence postoperative satisfaction and outcomes in patients undergoing total knee arthroplasty? A prospective cohort study. *BMC Musculoskeletal Disorders,* 22**,** 647.

BIERKE, S. & PETERSEN, W. 2017. Influence of anxiety and pain catastrophizing on the course of pain within the first year after uncomplicated total knee replacement: a prospective study. *Archives of Orthopaedic & Trauma Surgery,* 137**,** 1735-1742.

BIN, S. I. & NAM, T. S. 2007. Early results of high-flex total knee arthroplasty: comparison study at 1 year after surgery. *Knee Surgery, Sports Traumatology, Arthroscopy,* 15**,** 350-5.

BISTOLFI, A., BETTONI, E., APRATO, A., MILANI, P., BERCHIALLA, P., GRAZIANO, E., MASSAZZA, G. & LEE, G. C. 2017. The presence and influence of mild depressive symptoms on post-operative pain perception following primary total knee arthroplasty. *Knee Surgery, Sports Traumatology, Arthroscopy,* 25**,** 2792-2800.

© 2022 Olsen U et al. *JAMA Network Open.*

BLACKBURN, J., QURESHI, A., AMIRFEYZ, R. & BANNISTER, G. 2012. Does preoperative anxiety and depression predict satisfaction after total knee replacement? *Knee,* 19**,** 522-4.
BOERGER, T. O., AGLIETTI, P., MONDANELLI, N. & SENSI, L. 2005. Mini-subvastus versus medial parapatellar approach in total knee arthroplasty. *Clinical Orthopaedics & Related Research,* 440**,** 82-7.
BOSSMANN, T., BRAUNER, T., WEARING, S. & HORSTMANN, T. 2017. Predictors of chronic pain following total knee replacement in females and males: an exploratory study. *Pain Manag,* 7**,** 391-403.
BOVE, A. M., HAUSMANN, L. R. M., PIVA, S. R., BRACH, J. S., LEWIS, A. & FITZGERALD, G. K. 2022. Race Differences in Postacute Physical Therapy Utilization and Patient-Reported Function After Total Knee Arthroplasty. *Arthritis Care Res (Hoboken),* 74**,** 79-88.
BRANDER, V. A., DAVID STULBERG, S., ADAMS, A. D., HARDEN, R. N., BRUEHL, S., STANOS, S. P. & HOULE, T. 2003. Predicting Total Knee Replacement Pain: A Prospective, Observational Study. *Clinical Orthopaedics and Related Research***,** 27-36.
BROCK, T. M., SHIRLEY, M., BARDGETT, M., WALKER, M. & DEEHAN, D. J. 2017. Inadequate pre-operative glycaemic control in patients with diabetes mellitus adversely influences functional recovery after total knee arthroplasty : Patients with impaired glycaemic control exhibit poorer functional outcomes at 1-year post-arthroplasty. *Knee Surgery, Sports Traumatology, Arthroscopy,* 25**,** 1801-1806.
BROCKENBROUGH, G. 2007. Radiographic severity of OA may not predict TKA outcomes. *Orthopedics Today,* 27**,** 9-9.
BROWNE, J. A. 2013. Movement pain, resting pain and depression prior to total knee replacement predict postoperative pain. *Evidence-Based Nursing,* 16**,** 115-6.
BRUMMETT, C. M., HALLSTROM, B., URQUHART, A., MORRIS, M., CLAUW, D. J. & WILLIAMS, D. A. 2011. Psychological predictors of failure to improve after lower extremity joint arthroplasty. *Arthritis and Rheumatism. Conference: Annual Scientific Meeting of the American College of Rheumatology and Association of Rheumatology Health Professionals,* 63.
BRUMMETT, C. M., URQUHART, A., HALLSTROM, B., TSODIKOV, A., WILLIAMS, D. A. & CLAUW, D. J. 2013. The impact of centralized pain on long-term analgesic response to lower extremity joint arthroplasty: A prospective, observational cohort study. *Arthritis and Rheumatism,* 10**,** S898-S899.
BRUMMETT, C. M., URQUHART, A. G., HASSETT, A. L., TSODIKOV, A., HALLSTROM, B. R., WOOD, N. I., WILLIAMS, D. A. & CLAUW, D. J. 2015. Characteristics of Fibromyalgia Independently Predict Poorer Long-Term Analgesic Outcomes Following Total Knee and Hip Arthroplasty. *Arthritis & Rheumatology,* 67**,** 1386-1394.
BRAAKSMA, C., OEHLERS, V., VEEN, M. R. & WOLTERBEEK, N. 2020. Patient characteristics do not predict the change in physical functioning following arthroplasty measured by the HOOS-PS and KOOS-PS. *Journal of Orthopaedics,* 20**,** 122-124.
BUMBERGER, A., BORST, K., HOBUSCH, G. M., WILLEGGER, M., STELZENEDER, D., WINDHAGER, R., DOMAYER, S. & WALDSTEIN, W. 2021. Higher patient knowledge and resilience improve the functional outcome of primary total knee arthroplasty. *Wiener Klinische Wochenschrift,* 133**,** 543-549.
BUVANENDRAN, A., MORIC, M., KROIN, J. & TUMAN, K. J. 2011. Risk factors and mechanisms for persistent postsurgical pain after total knee replacement. *Anesthesia and Analgesia. Conference,* 112.
BUVANENDRAN, A., MORIC, M., KROIN, J. & TUMAN, K. J. 2012. Persistent postsurgical pain after total knee replacement: Preoperative health status as a risk factor? *Anesthesia and Analgesia,* 1**,** S374.
CARACCIOLO, B. & GIAQUINTO, S. 2005. Determinants of the subjective functional outcome of total joint arthroplasty. *Archives of Gerontology & Geriatrics,* 41**,** 169-176.
CARVALHO JUNIOR, L. H., TEIXEIRA, B. P., BERNARDES, C. O., SOARES, L. F., GONCALVES, M. B. & TEMPONI, E. F. 2017. Range of motion predictability after total knee arthroplasty with medial pivot prosthesis. *Revista Brasileira de Ortopedia,* 52**,** 197-202.
CHALIDIS, B. E., PETSATODIS, G., CHRISTODOULOU, A. G., CHRISTOFORIDIS, J., PAPADOPOULOS, P. P. & POURNARAS, J. 2010. Is obesity a contraindication for minimal invasive total knee replacement? A prospective randomized control trial. *Obesity Surgery,* 20**,** 1633-41.
CHANG, C. B., YOO, J. H., KOH, I. J., KANG, Y. G., SEONG, S. C. & KIM, T. K. 2010. Key factors in determining surgical timing of total knee arthroplasty in osteoarthritic patients: age, radiographic severity, and symptomatic severity. *Journal of Orthopaedics & Traumatology,* 11**,** 21-7.
CHEN, F., GAO, W., HU, J., YANG, X., CHAI, X. & WANG, D. 2021. Preoperative angiotensin II type 2 receptor is a predictor for developing chronic post-surgical pain after total knee arthroplasty surgery. *Life Sci,* 278**,** 119654.

CHENG, T., LIU, T., ZHANG, G., PENG, X. & ZHANG, X. 2010. Does minimally invasive surgery improve short-term recovery in total knee arthroplasty? *Clinical Orthopaedics & Related Research,* 468**,** 1635-1648.
CHEUY, V. A., LOYD, B. J., HAFNER, W., KITTELSON, A. J., WAUGH, D. & STEVENS-LAPSLEY, J. E. 2019. Influence of Diabetes Mellitus on the Recovery Trajectories of Function, Strength, and Self-Report Measures After Total Knee Arthroplasty. *Arthritis Care and Research.*
CHOUTEAU, J., LERAT, J. L., TESTA, R., MOYEN, B., FESSY, M. H. & BANKS, S. A. 2009. Kinematics of a cementless mobile bearing posterior cruciate ligament-retaining total knee arthroplasty. *Knee,* 16**,** 223-7.
CHOWDHRY, M., BAMNE, A. B., NA, Y. G., KANG, Y. G. & KIM, T. K. 2014. Prevalence and predictors of post-operative coronal alignment outliers and their association with the functional outcomes in navigated total knee arthroplasty. *Journal of Arthroplasty,* 29**,** 2357-62.
CHRISTENSEN, J., PETERS, C., GILILLAND, J., STODDARD, G. & PELT, C. 2021. Physical activity, pain interference and comorbidities relate to PROMIS physical function in younger adults following total knee arthroplasty. *Disabil Rehabil,* 43**,** 3741-3747.
CHRISTENSEN, J. C., MIZNER, R. L., FOREMAN, K. B., LASTAYO, P. C., PETERS, C. L. & PELT, C. E. 2019. Preoperative Quadriceps Weakness Preferentially Predicts Postoperative Aberrant Movement Patterns During High-Demand Mobility Following Total Knee Arthroplasty. *The Knee,* 26**,** 79.
CLEMENT, N. D., JENKINS, P. J., MACDONALD, D., NIE, Y. X., PATTON, J. T., BREUSCH, S. J., HOWIE, C. R. & BIANT, L. C. 2013a. Socioeconomic status affects the Oxford knee score and short-form 12 score following total knee replacement. *Bone & Joint Journal,* 95**,** 52-8.
CLEMENT, N. D., MACDONALD, D., BURNETT, R. & BREUSCH, S. J. 2013b. Diabetes does not influence the early outcome of total knee replacement: a prospective study assessing the Oxford knee score, short form 12, and patient satisfaction. *Knee,* 20**,** 437-41.
CLEMENT, N. D., MACDONALD, D., HOWIE, C. R. & BIANT, L. C. 2011. The outcome of primary total hip and knee arthroplasty in patients aged 80 years or more. *Journal of Bone & Joint Surgery - British Volume,* 93**,** 1265-70.
CLEMENT, N. D., MACDONALD, D., SIMPSON, A. H. R. W. & BURNETT, R. 2013c. Total knee replacement in patients with concomitant back pain results in a worse functional outcome and a lower rate of satisfaction. *Bone and Joint Journal,* 95**,** 1632-1639.
COLLINS, J. E., DONNELL-FINK, L. A., YANG, H. Y., USISKIN, I. M., LAPE, E. C., WRIGHT, J., KATZ, J. N. & LOSINA, E. 2017. Effect of Obesity on Pain and Functional Recovery Following Total Knee Arthroplasty. *Journal of Bone & Joint Surgery - American Volume,* 99**,** 1812-1818.
COLLINS, J. E., YANG, H. Y., USISKIN, I. M., KATZ, J. N. & LOSINA, E. 2016. Does morbid obesity negatively affect patient reported outcomes following total knee arthroplasty? *Arthritis and Rheumatology,* 68**,** 4003-4004.
COOPER, N. A., RAKEL, B. A., ZIMMERMAN, B., TONELLI, S. M., HERR, K. A., CLARK, C. R., NOISEUX, N. O., CALLAGHAN, J. J. & SLUKA, K. A. 2017. Predictors of multidimensional functional outcomes after total knee arthroplasty. *Journal of Orthopaedic Research,* 35**,** 2790-2798.
CORNELIUS, M., EDWARDS, R., LAZARIDOU, A. & FRANCESCHELLI, O. 2017. Pain catastrophizing predicts post-surgical changes in physical functioning in total knee replace-ment patients. *Journal of Pain,* 18**,** S46.
CORNELIUS, M., WALKER, J., PEJSA, M., HAND, M., CAMPBELL, C., HAYTHORNTHWAITE, J., KHANUJA, P., STERLING, R., SMITH, M. & EDWARDS, R. 2015. Pre-surgical Quantitative Sensory Testing predicts persistent postoperative pain in total knee replacement patients. *Journal of Pain,* 1**,** S26.
CREMEANS-SMITH, J. K., CONTRERA, K., SPEERING, L., MILLER, E. T., PFEFFERLE, K. & DELAHANTY, D. L. 2012. Using established predictors of post-traumatic stress to explain variations in recovery outcomes among orthopaedic patients. *Psychosomatic Medicine,* 74**,** A22-A23.
CREMEANS-SMITH, J. K., CONTRERA, K., SPEERING, L., MILLER, E. T., PFEFFERLE, K., GREENE, K. & DELAHANTY, D. L. 2015a. Using established predictors of post-traumatic stress to explain variations in recovery outcomes among orthopedic patients. *Journal of Health Psychology,* 20**,** 1296-304.
CREMEANS-SMITH, J. K., GREENE, K. & DELAHANTY, D. L. 2013. Post-operative pain: Does trauma history exacerbate or mollify the experience? *Psychosomatic Medicine,* 75**,** A46-A47.
CREMEANS-SMITH, J. K., GREENE, K. & DELAHANTY, D. L. 2015b. Trauma history as a resilience factor for patients recovering from total knee replacement surgery. *Psychology & Health,* 30**,** 1005-16.
CREMEANS-SMITH, J. K., GREENE, K. & DELAHANTY, D. L. 2016. Physiological Indices of Stress Prior to and Following Total Knee Arthroplasty Predict the Occurrence of Severe Post-Operative Pain. *Pain Medicine,* 17**,** 970-9.

CREMEANS-SMITH, J. K., GREENE, K. & DELAHANTY, D. L. 2018. Application of the cortisol-CRP ratio to a surgical population: Predicting depression, pain, and stress reactivity among patients undergoing total knee arthroplasty. *Psychosomatic Medicine,* 80**,** A127.

DAILIANA, Z. H., PAPAKOSTIDOU, I., VARITIMIDIS, S., LIAROPOULOS, L., ZINTZARAS, E., KARACHALIOS, T., MICHELINAKIS, E. & MALIZOS, K. N. 2015. Patient-reported quality of life after primary major joint arthroplasty: a prospective comparison of hip and knee arthroplasty. *BMC Musculoskeletal Disorders,* 16**,** 366.

DALURY, D. F., MULLIKEN, B. D., ADAMS, M. J., LEWIS, C., SAUDER, R. R., BUSHEY, J. A., DALURY, D. F., MULLIKEN, B. D., ADAMS, M. J., LEWIS, C., SAUDER, R. R. & BUSHEY, J. A. 2009. Early recovery after total knee arthroplasty performed with and without patellar eversion and tibial translation. A prospective randomized study. *Journal of Bone & Joint Surgery, American Volume,* 91**,** 1339-1343.

DAVE, A. J., SELZER, F., LOSINA, E., USISKIN, I., COLLINS, J. E., LEE, Y. C., BAND, P., DALURY, D. F., IORIO, R., KINDSFATER, K. & KATZ, J. N. 2017. The association of pre-operative body pain diagram scores with pain outcomes following total knee arthroplasty. *Osteoarthritis & Cartilage,* 25**,** 667-675.

DAVIS, A., BADLEY, E. M., HOGG-JOHNSON, S., IBRAHIM, S., PERRUCCIO, A. V., WONG, R. & BEATON, D. E. 2009. Understanding early recovery following primary total hip and knee replacement. *Arthritis and Rheumatism,* 10**,** 1938.

DAVIS, A., IBRAHIM, S., HOGG-JOHNSON, S., WONG, R., BEATON, D., CHESWORTH, B., GANDHI, R., MAHOMED, N., PERRUCCIO, A., RAJGOPAL, V. & WADDELL, J. 2017. Achieving important improvement in WOMAC pain and function impacts satisfaction 1 year following total knee replacement. *Osteoarthritis and Cartilage,* 25**,** S218.

DERE, D., PAKER, N., SOY BUGDAYCI, D. & TEKDOS DEMIRCIOGLU, D. 2014. Effect of body mass index on functional recovery after total knee arthroplasty in ambulatory overweight or obese women with osteoarthritis. *Acta Orthopaedica et Traumatologica Turcica,* 48**,** 117-21.

DESMEULES, F., DIONNE, C. E., BELZILE, E. L., BOURBONNAIS, R., CHAMPAGNE, F. & FREMONT, P. 2013. Determinants of pain, functional limitations and health-related quality of life six months after total knee arthroplasty: results from a prospective cohort study. *BMC Sports Science, Medicine and Rehabilitation,* 5**,** 2.

DIERICK, F., AVENIERE, T., COSSEMENT, M., POILVACHE, P., LOBET, S. & DETREMBLEUR, C. 2004. Outcome assessment in osteoarthritic patients undergoing total knee arthroplasty. *Acta Orthopaedica Belgica,* 70**,** 38-45.

DJADOUN, S., FRANCE, M. N., GROSU, I., THIENPONT, E. & LAVAND'HOMME, P. 2014. Impact of diabetes mellitus on nature and quality of persistent pain after total knee arthroplasty. *Regional Anesthesia and Pain Medicine,* 1**,** e286.

DOSSETT, H. G., SWARTZ, G. J., ESTRADA, N. A., LEFEVRE, G. W. & KWASMAN, B. G. 2012. Kinematically versus mechanically aligned total knee arthroplasty. *Orthopedics,* 35**,** e160-9.

DOURY-PANCHOUT, F., METIVIER, J. C. & FOUQUET, B. 2015. Kinesiophobia negatively influences recovery of joint function following total knee arthroplasty. *European journal of physical & rehabilitation medicine.,* 51**,** 155-61.

DOWSEY, M. M., BROADHEAD, M. L., STONEY, J. D. & CHOONG, P. F. 2009. Outcomes of total knee arthroplasty in English- versus non-English-speaking patients. *Journal of Orthopaedic Surgery,* 17**,** 305-9.

DOWSEY, M. M., NIKPOUR, M. & CHOONG, P. F. 2014. Outcomes following large joint arthroplasty: does socio-economic status matter? *BMC Musculoskeletal Disorders,* 15**,** 148.

DOWSEY, M. M., SMITH, A. J. & CHOONG, P. F. M. 2015. Latent Class Growth Analysis predicts long term pain and function trajectories in total knee arthroplasty: a study of 689 patients. *Osteoarthritis & Cartilage,* 23**,** 2141-2149.

DOWSEY, M. M., SPELMAN, T. & CHOONG, P. F. M. 2016. Development of a Prognostic Nomogram for Predicting the Probability of Nonresponse to Total Knee Arthroplasty 1 Year After Surgery. *Journal of Arthroplasty,* 31**,** 1654-1660.

DUIVENVOORDEN, T., VISSERS, M. M., VERHAAR, J. A., BUSSCHBACH, J. J., GOSENS, T., BLOEM, R. M., BIERMA-ZEINSTRA, S. M. & REIJMAN, M. 2013. Anxiety and depressive symptoms before and after total hip and knee arthroplasty: a prospective multicentre study. *Osteoarthritis & Cartilage,* 21**,** 1834-40.

DUMENCI, L., PERERA, R. A., KEEFE, F. J., ANG, D. C., SLOVER, J., JENSEN, M. P. & RIDDLE, D. L. 2019. Model-based pain and function outcome trajectory types for patients undergoing knee arthroplasty: a secondary analysis from a randomized clinical trial. *Osteoarthritis Cartilage,* 27**,** 878-884.

DURSTELER, C., SALAZAR, Y., RODRIGUEZ, U., PELFORT, X. & VERDIE, L. P. 2021. Conditioned pain modulation predicts persistent pain after knee replacement surgery. *Pain Rep,* 6**,** e910.
DUTKA, J., SKOWRONEK, M., SOSIN, P. & SKOWRONEK, P. 2011. Subvastus and medial parapatellar approaches in TKA: comparison of functional results. *Orthopedics,* 34**,** 148.
DUTTON, A. Q., YEO, S. J., YANG, K. Y., LO, N. N., CHIA, K. U., CHONG, H. C., DUTTON, A. Q., YEO, S.-J., YANG, K.-Y., LO, N.-N., CHIA, K.-U. & CHONG, H.-C. 2008. Computer-assisted minimally invasive total knee arthroplasty compared with standard total knee arthroplasty. A prospective, randomized study. *Journal of Bone & Joint Surgery, American Volume,* 90**,** 2-9.
EDWARDS, R. R., HAYTHORNTHWAITE, J. A., SMITH, M. T., KLICK, B. & KATZ, J. N. 2009. Catastrophizing and depressive symptoms as prospective predictors of outcomes following total knee replacement. *Pain Research & Management,* 14**,** 307-11.
ELLIS, H. B., HOWARD, K. J., KHALEEL, M. A. & BUCHOLZ, R. 2012. Effect of psychopathology on patient-perceived outcomes of total knee arthroplasty within an indigent population. *Journal of Bone and Joint Surgery - Series A,* 94**,** e84.1-e84.8.
FALLER, H., KIRSCHNER, S. & KONIG, A. 2003. Psychological distress predicts functional outcomes at three and twelve months after total knee arthroplasty. *General Hospital Psychiatry,* 25**,** 372-373.
FARAHINI, H., MOGHTADAEI, M., BAGHERI, A. & AKBARIAN, E. 2012. Factors influencing range of motion after total knee arthroplasty. *Iranian Red Crescent Medical Journal,* 14**,** 417-21.
FARIN, E., GLATTACKER, M. & JACKEL, W. H. 2006. Predictors of rehabilitation outcome in patients after total hip and total knee arthroplasty - A multilevel analysis. [German]. *Physikalische Medizin Rehabilitationsmedizin Kurortmedizin,* 16**,** 82-91.
FERNANDEZ-FAIREN, M., HERNÁNDEZ-VAQUERO, D., MURCIA, A., TORRES, A. & LLOPIS, R. 2013. Trabecular metal in total knee arthroplasty associated with higher knee scores: a randomized controlled trial. *Clinical Orthopaedics & Related Research,* 471**,** 3543-3553.
FERREIRA, A. M., SALIM, R., FOGAGNOLO, F., DE OLIVEIRA, L. F. L., RIBERTO, M. & KFURI, M. 2021. The Value of a Standardized Knee Functional Assessment in Predicting the Outcomes of Total Knee Arthroplasty. *J Knee Surg.*
FERRER, T., HINAREJOS, P., GOICOECHEA, N., LEAL-BLANQUET, J., SANCHEZ-SOLER, J., TORRES-CLARAMUNT, R. & MONLLAU, J. C. 2020. Anxiety is the cause of the worse outcomes of allergic patients after total knee arthroplasty. *Knee Surg Sports Traumatol Arthrosc,* 28**,** 3135-3141.
FILARDO, G., MERLI, G., ROFFI, A., MARCACCI, T., BERTI CERONI, F., RABONI, D., BORTOLOTTI, B., KON, E. & MARCACCI, M. 2017. Kinesiophobia and depression affect total knee arthroplasty outcome in a multivariate analysis of psychological and physical factors on 200 patients. *Knee Surgery, Sports Traumatology, Arthroscopy,* 25**,** 3417-3423.
FILBAY, S. R. & JUDGE, A. 2017. Evaluating patients' expectations from a novel patientcentered perspective predicts surgical outcome and dissatisfaction following knee arthroplasty. *Osteoarthritis and Cartilage,* 25**,** S336-S337.
FILBAY, S. R., JUDGE, A., DELMESTRI, A., ARDEN, N. K., ALTMAN, D., BEARD, D., CARR, A., COOPER, C., CULLIFORD, D., GRIFFIN, T., JAVAID, K., LATHAM, J., MURRAY, D., PINEDO-VILLANUEVA, R., PRICE, A. & PRIETO-ALHAMBRA, D. 2018. Evaluating Patients' Expectations From a Novel Patient-Centered Perspective Predicts Knee Arthroplasty Outcome. *Journal of Arthroplasty,* 33**,** 2146-2152.e4.
FITZPATRICK, J., BADLEY, E. M., RAMPERSAUD, Y. R., POWER, J. D., GANDHI, R., VEILLETTE, C., MAHOMED, N. N., DAVEY, J. R., SYED, K. & PERRUCCIO, A. V. 2017. The influence of patient characteristics on pain following total joint arthroplasty for osteoarthritis: Effect modification by sex and age. *Osteoarthritis and Cartilage,* 25**,** S340.
FITZSIMMONS, M., CARR, E., WOODHOUSE, L. & BOSTICK, G. P. 2018. Development and Persistence of Suspected Neuropathic Pain After Total Knee Arthroplasty in Individuals With Osteoarthritis. *PM R,* 10**,** 903-909.
FLEETON, G., HARMER, A. R., NAIRN, L., CROSBIE, J., MARCH, L., CRAWFORD, R., VAN DER ESCH, M. & FRANSEN, M. 2016. Self-Reported Knee Instability Before and After Total Knee Replacement Surgery. *Arthritis care & research,* 68**,** 463-71.
FORAN, J. R., MONT, M. A., ETIENNE, G., JONES, L. C. & HUNGERFORD, D. S. 2004. The outcome of total knee arthroplasty in obese patients. *Journal of Bone & Joint Surgery - American Volume,* 86**,** 1609-15.

© 2022 Olsen U et al. *JAMA Network Open.*

FORSYTHE, M. E., DUNBAR, M. J., HENNIGAR, A. W., SULLIVAN, M. J. L. & GROSS, M. 2008. Prospective relation between catastrophizing and residual pain following knee arthroplasty: Two-year follow-up. *Pain Research and Management,* 13**,** 335-341.

FRANKLIN, P. D., HARROLD, L. R., LI, W., ALLISON, J., AYERS, D. & LEWIS, C. 2013. Important predictors of patient-reported outcomes after TKR and THR are not included in risk models based on administrative data. *Arthritis and Rheumatism,* 10**,** S910.

FRANKLIN, P. D., LI, W. & AYERS, D. C. 2008. The Chitranjan Ranawat Award: functional outcome after total knee replacement varies with patient attributes. *Clinical Orthopaedics & Related Research,* 466**,** 2597-604.

FURU, M., ITO, H., NISHIKAWA, T., NANKAKU, M., KURIYAMA, S., ISHIKAWA, M., NAKAMURA, S., AZUKIZAWA, M., HAMAMOTO, Y. & MATSUDA, S. 2016. Quadriceps strength affects patient satisfaction after total knee arthroplasty. *Journal of Orthopaedic Science,* 21**,** 38-43.

GANDHI, R., DAVEY, J. R. & MAHOMED, N. 2009a. Patient expectations predict greater pain relief with joint arthroplasty. *Journal of Arthroplasty,* 24**,** 716-21.

GANDHI, R., RAZAK, F., DAVEY, J. R. & MAHOMED, N. N. 2010a. Metabolic syndrome and the functional outcomes of hip and knee arthroplasty. *Journal of Rheumatology,* 37**,** 1917-22.

GANDHI, R., RAZAK, F., DAVEY, J. R. & MAHOMED, N. N. 2010b. Metabolic syndrome and the functional outcomes of hip and knee arthroplasty. *Journal of Rheumatology,* 37**,** 1917-1922.

GANDHI, R., RAZAK, F., DAVEY, J. R., RAMPERSAUD, Y. R. & MAHOMED, N. N. 2010c. Effect of sex and living arrangement on the timing and outcome of joint replacement surgery. *Canadian Journal of Surgery,* 53**,** 37-41.

GANDHI, R., RAZAK, F., TSO, P., DAVEY, J. R. & MAHOMED, N. N. 2009b. Greater perceived helplessness in osteoarthritis predicts outcome of joint replacement surgery. *Journal of Rheumatology,* 36**,** 1507-11.

GANDHI, R., TSO, P., DAVIS, A. & MAHOMED, N. N. 2009c. Outcomes of total joint arthroplasty in academic versus community hospitals. *Canadian Journal of Surgery,* 52**,** 413-6.

GANDHI, R., TSVETKOV, D., DAVEY, J. R., SYED, K. A. & MAHOMED, N. N. 2009d. Relationship between self-reported and performance-based tests in a hip and knee joint replacement population. *Clinical Rheumatology,* 28**,** 253-257.

GATES, L. S., BOWEN, C. J. & ARDEN, N. K. 2016. Can clinical foot and ankle assessments improve the prediction of patient reported outcomes in knee arthroplasty? *Osteoarthritis and Cartilage,* 1**,** S208.

GATES, L. S., BOWEN, C. J., SANCHEZ-SANTOS, M. T., DELMESTRI, A. & ARDEN, N. K. 2017. Do foot & ankle assessments assist the explanation of 1 year knee arthroplasty outcomes? *Osteoarthritis & Cartilage,* 25**,** 892-898.

GATHA, N. M., CLARKE, H. D., FUCHS, R., SCUDERI, G. R. & INSALL, J. N. 2004. Factors affecting postoperative range of motion after total knee arthroplasty. *The Journal of Knee Surgery,* 17**,** 196-202.

GETACHEW, M., LERDAL, A., SMASTUEN, M. C., GAY, C. L., AAMODT, A., TESFAYE, M. & LINDBERG, M. F. 2021. High levels of preoperative pain and fatigue are red flags for moderate-severe pain 12 months after total knee arthroplasty-A longitudinal cohort study. *Musculoskeletal Care,* 19**,** 186-192.

GIESINGER, J. M., GIESINGER, K., LOTH, F. L., SIMPSON, H. A., HOWIE, C. & HAMILTON, D. F. 2016. Impact of obesity on patient-reported outcomes after total knee replacement. *Value in Health,* 19**,** A544.

GIORDANO, R., PETERSEN, K. K., ANDERSEN, H. H., LICHOTA, J., VALERIANI, M., SIMONSEN, O. & ARENDT-NIELSEN, L. 2020. Preoperative serum circulating microRNAs as potential biomarkers for chronic postoperative pain after total knee replacement. *Mol Pain,* 16**,** 1744806920962925.

GIORDANO, R., PETERSEN, K. K., SANTORO, M., PAZZAGLIA, C., SIMONSEN, O., VALERIANI, M. & ARENDT-NIELSEN, L. 2021. Circulating long non-coding RNA signature in knee osteoarthritis patients with postoperative pain one-year after total knee replacement. *Scand J Pain,* 21**,** 823-830.

GONZALEZ SAENZ DE TEJADA, M., ESCOBAR, A., BILBAO, A., HERRERA-ESPINEIRA, C., GARCIA-PEREZ, L., AIZPURU, F. & SARASQUETA, C. 2014. A prospective study of the association of patient expectations with changes in health-related quality of life outcomes, following total joint replacement. *BMC Musculoskeletal Disorders,* 15**,** 248.

GRAVES, C. M., OTERO, J. E., GAO, Y., GOETZ, D. D., WILLENBORG, M. D. & CALLAGHAN, J. J. 2014. Patient reported allergies are a risk factor for poor outcomes in total hip and knee arthroplasty. *Journal of Arthroplasty,* 29**,** 147-9.

GRAY, A., RAMOS, S., HOWARD, K., BRYSON, B. & ELLIS, H. B. 2017. The Pain Disability Questionnaire (PDQ): Evaluating the efficacy of the psychosocial and functional subscales for 12-month post-treatment outcomes after total knee arthroplasty. *Current Orthopaedic Practice,* 28**,** 573-579.

© 2022 Olsen U et al. *JAMA Network Open.*

GRECO, C., BELFER, I., VULAKOVICH, K., LANDSITTEL, D., DAI, F. & CHELLY, J. 2017. Associations of psychosocial factors with chronic postsurgical pain following total knee arthroplasty. *Journal of Pain,* 18**,** S76.

GREENIDGE, N., DAVIS, A. M., HAWKER, G. A. & BADLEY, E. M. 2009. The effect of pre-operative number of affected joints on pain and function following total joint replacement for OA: A longitudinal study. *Osteoarthritis and Cartilage,* 1**,** S194.

GROSU, I., THIENPONT, E., FRANCE, M. N. & LAVAND'HOMME, P. 2013. Persistent pain after total knee arthroplasty (TKA): Incidence, characteristics and predictive factors. *Regional Anesthesia and Pain Medicine. Conference: 38th Annual ASRA Regional Anesthesia and Acute Pain Medicine Meeting,* 38.

GROUP, K. A. T. T., JOHNSTON, L., MACLENNAN, G., MCCORMACK, K., RAMSAY, C. & WALKER, A. 2009. The Knee Arthroplasty Trial (KAT) design features, baseline characteristics, and two-year functional outcomes after alternative approaches to knee replacement. *Journal of Bone & Joint Surgery - American Volume,* 91**,** 134-41.

GUIMARAES-PEREIRA, L., VALDOLEIROS, I., REIS, P. & ABELHA, F. 2016. Evaluating persistent postoperative pain in one tertiary hospital: Incidence, quality of life, associated factors, and treatment. *Anesthesiology and Pain Medicine,* 6.

GØTHESEN, O., ESPEHAUG, B., HAVELIN, L. I., PETURSSON, G., HALLAN, G., STRØM, E., DYRHOVDEN, G. & FURNES, O. 2014. Functional outcome and alignment in computer-assisted and conventionally operated total knee replacements: a multicentre parallel-group randomised controlled trial. *Bone & Joint Journal,* 96**,** 609-618.

HA, C. & HA, H. 2006. Minimally Invasive vs. Standard Total Knee Arthroplasty: a Prospective Randomized Comparison Study. *Journal of the korean orthopaedic association,* 41**,** 841-849.

HALKET, A., STRATFORD, P. W., KENNEDY, D. M. & WOODHOUSE, L. J. 2010. Using hierarchical linear modeling to explore predictors of pain after total hip and knee arthroplasty as a consequence of osteoarthritis. *Journal of Arthroplasty,* 25**,** 254-62.

HAMILTON, D. F., BURNETT, R., PATTON, J. T., HOWIE, C. R., MORAN, M., SIMPSON, A. H. R. W. & GASTON, P. 2015. Implant design influences patient outcome after total knee arthroplasty: a prospective double-blind randomised controlled trial. *Bone & Joint Journal,* 97**,** 64-70.

HAMILTON, D. F., LOTH, F. L., GIESINGER, J. G., GIESINGER, K., MACDONALD, D. J., SIMPSON, H. & HOWIE, C. R. 2017. Impact of obesity on patient-reported outcomes following total knee arthroplasty. *Journal of Orthopaedic Research. Conference,* 35.

HAMILTON, D. F., SHIM, J., HOWIE, C. R. & MACFARLANE, G. J. 2021. Patients follow three distinct outcome trajectories following total knee arthroplasty. *Bone Joint J,* 103-B**,** 1096-1102.

HANRATTY, B., BENNETT, D., THOMPSON, N. & BEVERLAND, D. 2011. A randomised controlled trial investigating the effect of posterior capsular stripping on knee flexion and range of motion in patients undergoing primary knee arthroplasty. *The knee,* 18**,** 474-479.

HANUSCH, B. C., O'CONNOR, D. B., IONS, P., SCOTT, A. & GREGG, P. J. 2014. Effects of psychological distress and perceptions of illness on recovery from total knee replacement. *Bone & Joint Journal,* 96**,** 210-6.

HARDEN, R. N., BRUEHL, S., STANOS, S., BRANDER, V., CHUNG, O. Y., SALTZ, S., ADAMS, A. & STULBERG, S. D. 2003. Prospective examination of pain-related and psychological predictors of CRPS-like phenomena following total knee arthroplasty: a preliminary study. *Pain,* 106**,** 393-400.

HASEGAWA, M., TONE, S., NAITO, Y. & SUDO, A. 2021. Preoperative pain catastrophizing affects pain outcome after total knee arthroplasty. *J Orthop Sci.*

HASHIMOTO, S., HATAYAMA, K., TERAUCHI, M., SAITO, K., HIGUCHI, H. & CHIKUDA, H. 2019. Preoperative hand-grip strength can be a predictor of stair ascent and descent ability after total knee arthroplasty in female patients. *Journal of Orthopaedic Science.*

HEMERT, W., SENDEN, R., GRIMM, B., LINDE, M., LATASTER, A. & HEYLIGERS, I. 2011. Early functional outcome after subvastus or parapatellar approach in knee arthroplasty is comparable. *Knee surgery, sports traumatology, arthroscopy,* 19**,** 943-951.

HINAREJOS, P., FERRER, T., LEAL, J., TORRES-CLARAMUNT, R., SANCHEZ-SOLER, J. & MONLLAU, J. C. 2016. Patient-reported allergies cause inferior outcomes after total knee arthroplasty. *Knee Surgery, Sports Traumatology, Arthroscopy,* 24**,** 3242-3246.

HIRSCHMANN, M. T., HOFFMANN, M., KRAUSE, R., JENABZADEH, R. A., ARNOLD, M. P. & FRIEDERICH, N. F. 2010. Anterolateral approach with tibial tubercle osteotomy versus standard medial approach for primary total knee arthroplasty: does it matter? *BMC Musculoskeletal Disorders,* 11**,** 167.

© 2022 Olsen U et al. *JAMA Network Open.*

HIRSCHMANN, M. T., TESTA, E., AMSLER, F. & FRIEDERICH, N. F. 2013. The unhappy total knee arthroplasty (TKA) patient: higher WOMAC and lower KSS in depressed patients prior and after TKA. *Knee Surgery, Sports Traumatology, Arthroscopy,* 21**,** 2405-11.
HITT, K., PIERCE, T., JAUREGUI, J., CHERIAN, J., ELMALLAH, R., LEIBOWITZ, E., LOGAN, S. & MONT, M. 2015. Use of a Flexible Intramedullary Rod and its Influence on Patient Satisfaction and Femoral Size in Total Knee Arthroplasty. *Journal of long-term effects of medical implants,* 25**,** 201-208.
HODGES, A., HARMER, A. R., DENNIS, S., NAIRN, L., MARCH, L., CRAWFORD, R., PARKER, D. & FRANSEN, M. 2018. Prevalence and determinants of physical activity and sedentary behaviour before and up to 12months after total knee replacement: a longitudinal cohort study. *Clinical Rehabilitation***,** 269215518769986.
HOFSTEDE, S. N., GADEMAN, M. G. J., STIJNEN, T., NELISSEN, R. G. H. H., MARANG-VAN DE MHEEN, P. J., BIERMA-ZEINSTRA, S. M. A., VAN DIJK, M., KAARSEMAKER, S., VAN KAMPEN, P. M., NOLTE, P. A., POOLMAN, R. W., PRONK, Y., REIJMAN, M., STEVENS, M., THOMASSEN, B. J. W., VERDEGAAL, S. H. M. & VLIET VLIELAND, T. P. M. 2018. The influence of preoperative determinants on quality of life, functioning and pain after total knee and hip replacement: A pooled analysis of Dutch cohorts. *BMC Musculoskeletal Disorders,* 19.
HOMMEL, H., ABDEL, M. & PERKA, C. 2017. Kinematic femoral alignment with gap balancing and patient-specific instrumentation in total knee arthroplasty: a randomized clinical trial. *European journal of orthopaedic surgery & traumatology : orthopedie traumatologie,* 27**,** 683-688.
HOOPER, G. J., ROTHWELL, A. G., HOOPER, N. M., FRAMPTON, C., HOOPER, G. J., ROTHWELL, A. G., HOOPER, N. M. & FRAMPTON, C. 2012. The relationship between the American Society Of Anesthesiologists physical rating and outcome following total hip and knee arthroplasty: an analysis of theNew Zealand Joint Registry. *Journal of Bone & Joint Surgery, American Volume,* 94**,** 1065-1070.
HOURLIER, H. & FENNEMA, P. 2014. Intraoperative fluoroscopy improves surgical precision in conventional TKA. *Knee surgery, sports traumatology, arthroscopy,* 22**,** 1619-1625.
HOVIK, L. H., WINTHER, S. B., FOSS, O. A. & GJEILO, K. H. 2016. Preoperative pain catastrophizing and postoperative pain after total knee arthroplasty: a prospective cohort study with one year follow-up. *BMC Musculoskeletal Disorders,* 17**,** 214.
HUGHES, A. J., RICHARDS, J. M., CAMPBELL, C. M., HAYTHORNTHWAITE, J. A., EDWARDS, R. R. & SMITH, M. T. 2018. Sleep-pain behaviors predict insomnia, pain, and physical function over one year following total knee arthroplasty in individuals with knee osteoarthritis. *Sleep,* 41**,** A338.
HYLKEMA, T. H., STEVENS, M., SELZER, F., AMICK, B. A., KATZ, J. N. & BROUWER, S. 2019. Activity Impairment and Work Productivity Loss After Total Knee Arthroplasty: A Prospective Study. *Journal of Arthroplasty.*
HAANSTRA, T. M., TILBURY, C., KAMPER, S. J., TORDOIR, R. L., VLIET VLIELAND, T. P., NELISSEN, R. G., CUIJPERS, P., DE VET, H. C., DEKKER, J., KNOL, D. L. & OSTELO, R. W. 2015. Can Optimism, Pessimism, Hope, Treatment Credibility and Treatment Expectancy Be Distinguished in Patients Undergoing Total Hip and Total Knee Arthroplasty? *PLoS ONE [Electronic Resource],* 10**,** e0133730.
INGLESHWAR, A., BARBO, A., LANDON, G. C., SIFF, S. J., DE ACHAVAL, S. & SUAREZ-ALMAZOR, M. E. 2013. Ethnic variations at time of surgery and during follow-up in patients undergoing total knee arthroplasty. *Arthritis and Rheumatism,* 10**,** S353.
JACOBS, C. A., CHRISTENSEN, C. P. & KARTHIKEYAN, T. 2016a. Greater medial compartment forces during TKA associated with improved patient satisfaction and function. *Journal of Orthopaedic Research. Conference,* 34.
JACOBS, C. A., CHRISTENSEN, C. P. & KARTHIKEYAN, T. 2016b. Persistent postoperative pain more common for total knee arthroplasty patients with an intact anterior cruciate ligament at the time of surgery. *Journal of Orthopaedic Research. Conference,* 34.
JAIN, D., NGUYEN, L. L., BENDICH, I., NGUYEN, L. L., LEWIS, C. G., HUDDLESTON, J. I., DUWELIUS, P. J., FEELEY, B. T. & BOZIC, K. J. 2017. Higher Patient Expectations Predict Higher Patient-Reported Outcomes, But Not Satisfaction, in Total Knee Arthroplasty Patients: A Prospective Multicenter Study. *Journal of Arthroplasty,* 32**,** S166-S170.
JAMSEN, E., VEKAMA, L. & PUOLAKKA, T. 2015. Self-rated health and the functional outcome of primary knee replacement in the aged. *European Geriatric Medicine,* 1**,** S149.
JARVENPAA, J., KETTUNEN, J., KROGER, H. & MIETTINEN, H. 2010a. Obesity may impair the early outcome of total knee arthroplasty. *Scandinavian Journal of Surgery: SJS,* 99**,** 45-9.

JARVENPAA, J., KETTUNEN, J., KROGER, H. & MIETTINEN, H. 2010b. Obesity may impair the early outcome of total knee arthroplasty. A prospective study of 100 patients. *Scandinavian Journal of Surgery,* 99**,** 45-49.
JEFFERIES, P., COFFEY, L. & GALLAGHER, P. 2012. The efficacy of psychosocial factors in predicting pain and functional outcomes following knee replacement surgery. *Evidence-Based Nursing,* 15**,** 92-3.
JIANG, Y., SANCHEZ-SANTOS, M. T., JUDGE, A. D., MURRAY, D. W. & ARDEN, N. K. 2017. Predictors of Patient-Reported Pain and Functional Outcomes Over 10 Years After Primary Total Knee Arthroplasty: A Prospective Cohort Study. *Journal of Arthroplasty,* 32**,** 92-100.e2.
JOLLES, B., GRZESIAK, A., EUDIER, A., DEJNABADI, H., VORACEK, C., PICHONNAZ, C., AMINIAN, K. & MARTIN, E. 2012. A randomised controlled clinical trial and gait analysis of fixed- and mobile-bearing total knee replacements with a five-year follow-up. 94. Available: http://onlinelibrary.wiley.com/o/cochrane/clcentral/articles/702/CN-00969702/frame.html [Accessed 2012-1-1].
JONBERGEN, H., SCHOLTES, V., KAMPEN, A. & POOLMAN, R. 2011. A randomised, controlled trial of circumpatellar electrocautery in total knee replacement without patellar resurfacing. *Journal of bone and joint surgery. British volume,* 93**,** 1054-1059.
JONES, C. A., COX, V., JHANGRI, G. S. & SUAREZ-ALMAZOR, M. E. 2012a. Delineating the impact of obesity and its relationship on recovery after total joint arthroplasties. *Osteoarthritis & Cartilage,* 20**,** 511-8.
JONES, C. A., JHANGRI, G. S. & SUAREZ-ALMAZOR, M. E. 2012b. Factors influencing long-term recovery of total knee arthroplasty. *Arthritis and Rheumatism,* 10**,** S467-S468.
JONES, C. A., VOAKLANDER, D. C. & SUAREZ-ALMA, M. E. 2003. Determinants of function after total knee arthroplasty. *Physical Therapy,* 83**,** 696-706.
JUDGE, A., ARDEN, N. K., COOPER, C., KASSIM JAVAID, M., CARR, A. J., FIELD, R. E. & DIEPPE, P. A. 2012. Predictors of outcomes of total knee replacement surgery. *Rheumatology,* 51**,** 1804-13.
JUDGE, A., DIEPPE, P. A., ARDEN, N. K., COOPER, C., CARR, A., JAVAID, K. & FIELD, R. 2010. Developing a predictive tool for outcomes of total knee replacement: South West London elective orthopaedic centre database. *Rheumatology,* 1**,** i74.
JULIE, K. C. S., CONTRERA, K., SPEERING, L., MILLER, E. T., PFEFFERLE, K., GREENE, K. & DELAHANTY, D. L. 2013. Using established predictors of post-traumatic stress to explain variations in recovery outcomes among orthopaedic patients. *Psychosomatic Medicine,* 75**,** A-149.
KAHLENBERG, C. A., TRIVELLAS, M., LEE, Y. Y. & PADGETT, D. E. 2018. Preoperative Valgus Alignment Does Not Predict Inferior Outcome of Total Knee Arthroplasty. *HSS Journal,* 14**,** 50-54.
KANG, J. H., HSIEH, M. S. & LIN, H. C. 2010. Comparison of treatment outcomes following total knee arthroplasty among patients with rheumatoid arthritis and osteoarthritis: a nationwide population-based study. *Rheumatology,* 49**,** 1409-10.
KATAKAM, A., BRAGDON, C. R., CHEN, A. F., MELNIC, C. M. & BEDAIR, H. S. 2021. Elevated Body Mass Index Is a Risk Factor for Failure to Achieve the Knee Disability and Osteoarthritis Outcome Score-Physical Function Short Form Minimal Clinically Important Difference Following Total Knee Arthroplasty. *J Arthroplasty,* 36**,** 1626-1632.
KATZ, J. N., WRIGHT, E. A., LINGARD, E. A. & LOSINA, E. 2011. Association between severe pain in the early months following total knee replacement and functional outcomes over five year follow-up. *Osteoarthritis and Cartilage,* 1**,** S41-S42.
KEENEY, B. J., KOENIG, K. M., PADDOCK, N. G., MOSCHETTI, W. E., SPARKS, M. B. & JEVSEVAR, D. S. 2017. Do Aggregate Socioeconomic Status Factors Predict Outcomes for Total Knee Arthroplasty in a Rural Population? *Journal of Arthroplasty,* 32**,** 3583-3590.
KELLY, M., RUMI, M., KOTHARI, M., PARENTIS, M., BAILEY, K., PARRISH, W. & PELLEGRINI, V. 2006. Comparison of the vastus-splitting and median parapatellar approaches for primary total knee arthroplasty: a prospective, randomized study. *Journal of bone and joint surgery. American volume,* 88**,** 715-720.
KENNEDY, D. M., STRATFORD, P. W., RIDDLE, D. L., HANNA, S. E. & GOLLISH, J. D. 2008. Assessing recovery and establishing prognosis following total knee arthroplasty. *Physical Therapy,* 88**,** 22-32.
KHANNA, V. 2016. Association between preoperative sarcopenia and functional outcome of the patients following total knee replacement. *Osteoporosis International,* 1**,** S300.
KILICARSLAN, K., YALCIN, N., CICEK, H., DOGRAMACI, Y., UGURLU, M., OZKAN, H. & YILDIRIM, H. 2011. The effect of total synovectomy in total knee arthroplasty: a prospective randomized controlled study. *Knee surgery, sports traumatology, arthroscopy,* 19**,** 932-935.

© 2022 Olsen U et al. *JAMA Network Open.*

KIM, S. H., YOON, K. B., YOON, D. M., YOO, J. H. & AHN, K. R. 2015. Influence of Centrally Mediated Symptoms on Postoperative Pain in Osteoarthritis Patients Undergoing Total Knee Arthroplasty: A Prospective Observational Evaluation. *Pain Practice,* 15**,** E46-53.

KIM, T. K., CHO, H. J., KANG, Y. G., KIM, S. J. & CHANG, C. B. 2009. Improved early clinical outcomes of RP/PS mobile-bearing total knee arthroplasties. *Clinical Orthopaedics & Related Research,* 467**,** 2901-10.

KO, Y., LO, N. N., YEO, S. J., YANG, K. Y., YEO, W., CHONG, H. C. & THUMBOO, J. 2010. Determining the optimal timing for total knee replacement. *Value in Health,* 13**,** A313.

KORNILOV, N., LINDBERG, M. F., GAY, C., SARAEV, A., KULIABA, T., ROSSELAND, L. A. & LERDAL, A. 2018. Higher physical activity and lower pain levels before surgery predict non-improvement of knee pain 1 year after TKA. *Knee Surgery, Sports Traumatology, Arthroscopy,* 26**,** 1698-1708.

KURIEN, T., ARENDT-NIELSEN, L., PETERSEN, K. K., GRAVEN-NIELSEN, T. & SCAMMELL, B. E. 2018. Preoperative Neuropathic Pain Like Symptoms and Central Pain Mechanisms in Knee Osteoarthritis Predicts Poor Outcome 6 Months After Total Knee Replacement Surgery. *Journal of Pain,* 16**,** 16.

LAM, L. O., SWIFT, S. & SHAKESPEARE, D. 2003. Fixed flexion deformity and flexion after knee arthroplasty. What happens in the first 12 months after surgery and can a poor outcome be predicted? *Knee,* 10**,** 181-5.

LAMB, S. E. & FROST, H. 2003. Recovery of mobility after knee arthroplasty: expected rates and influencing factors. *Journal of Arthroplasty,* 18**,** 575-582.

LAMPE, F., MARQUES, C. J., FIEDLER, F., SUFI-SIAVACH, A., CARITA, A. I. & MATZIOLIS, G. 2016. Patient-specific and intra-operatively modifiable factors assessed by computer navigation predict maximal knee flexion one year after TKA. *Knee Surgery, Sports Traumatology, Arthroscopy,* 24**,** 3457-3465.

LANGE, J., DISEGNA, S. T., YANG, W., LI, W. & FRANKLIN, P. D. 2016. Identifying preoperative patient characteristics that correlate with early improvement or decline following total knee arthroplasty. *Journal of Orthopaedic Research. Conference,* 34.

LARSEN, D. B., LAURSEN, M., EDWARDS, R. R., SIMONSEN, O., ARENDT-NIELSEN, L. & PETERSEN, K. K. 2021. The Combination of Preoperative Pain, Conditioned Pain Modulation, and Pain Catastrophizing Predicts Postoperative Pain 12 Months After Total Knee Arthroplasty. *Pain Medicine,* 22**,** 1583-1590.

LASKOW, T., ZHU, J., BUTA, B., ONI, J., SIEBER, F., BANDEEN-ROCHE, K., WALSTON, J., FRANKLIN, P. D. & VARADHAN, R. 2021. Risk Factors for Non-Resilient Outcomes in Older Adults after Total Knee Replacement in the FORCE-TJR Cohort. *J Gerontol A Biol Sci Med Sci,* 04.

LEBLEU, J., POILVACHE, H., MAHAUDENS, P., DE RIDDER, R. & DETREMBLEUR, C. 2019. Predicting physical activity recovery after hip and knee arthroplasty? A longitudinal cohort study. *Brazilian Journal of Physical Therapy,* 25**,** 30-39.

LEDIN, H., , P. & , L. 2012. Tourniquet use in total knee replacement does not improve fixation, but appears to reduce final range of motion. *Acta Orthopaedica,* 83**,** 499-503.

LEE, A., CHAN, S. K. C., SAMY, W., CHIU, C. H. & GIN, T. 2015. Effect of hypovitaminosis d on postoperative pain outcomes and short-term health-related quality of life after knee arthroplasty. *Medicine (United States),* 94**,** e1812.

LEUNG, Y. Y., CHAKRABORTY, B., LIM, Z., YEO, S., LO, N., TAN, M., WONG, S., CHONG, H., YEO, W., WYLDE, V., DIEPPE, P. & THUMBOO, J. 2017. Preoperative pain sensitization and total knee replacement outcome. *Osteoarthritis and Cartilage,* 25**,** S372.

LEUNG, Y. Y., LIM, Z., FAN, Q., WYLDE, V., XIONG, S., YEO, S. J., LO, N. N., CHONG, H. C., YEO, W., TAN, M. H., CHAKRABORTY, B., BAK-SIEW WONG, S. & THUMBOO, J. 2019. Pre-operative pressure pain thresholds do not meaningfully explain satisfaction or improvement in pain after knee replacement: a cohort study. *Osteoarthritis and Cartilage,* 27**,** 49-58.

LI, W., HARROLD, L. R., ALLISON, J., LEWIS, C., BOWEN, T., FRANKLIN, P. D. & AYERS, D. 2013. Does functional gain and pain relief after TKR and THR differ by patient obese status? *Arthritis and Rheumatism,* 10**,** S909-S910.

LIEBS, T. R., HERZBERG, W., ROTH-KROEGER, A. M., RÜTHER, W. & HASSENPFLUG, J. 2011. Women recover faster than men after standard knee arthroplasty. *Clinical Orthopaedics & Related Research,* 469**,** 2855-2865.

LINDBERG, M. F., MIASKOWSKI, C., RUSTØEN, T., ROSSELAND, L. A., COOPER, B. A. & LERDAL, A. 2016. Factors that can predict pain with walking, 12 months after total knee arthroplasty. *Acta Orthopaedica,* 87**,** 600-606.

LINDNER, M., NOSSEIR, O., KELLER-PLIESSNIG, A., TEIGELACK, P., TEUFEL, M. & TAGAY, S. 2018. Psychosocial predictors for outcome after total joint arthroplasty: a prospective comparison of hip and knee arthroplasty. *BMC Musculoskeletal Disorders,* 19**,** 159.

LINGARD, E. A., KATZ, J. N., WRIGHT, E. A. & SLEDGE, C. B. 2004. Predicting the outcome of total knee arthroplasty. *Journal of Bone and Joint Surgery - Series A,* 86**,** 2179-2186.
LIU, K., YANG, D., ZAN, P., FAN, A., ZHENG, Z., JIANG, W. & LI, G. 2020. Preoperative low scores of Life Satisfaction Rating predicts poor outcomes after total knee arthroplasty: a prospective observational study. *Journal of Orthopaedic Surgery,* 15**,** 145.
LIZAUR-UTRILLA, A., SANZ-REIG, J. & TRIGUEROS-RENTERO, M. 2012. Greater satisfaction in older patients with a mobile-bearing compared with fixed-bearing total knee arthroplasty. *Journal of arthroplasty,* 27**,** 207-212.
LUNGU, E., DESMEULES, F., DIONNE, C. E., BELZILE, E. L. & VENDITTOLI, P. A. 2014. Prediction of poor outcomes six months following total knee arthroplasty in patients awaiting surgery. *BMC Musculoskeletal Disorders,* 15**,** 299.
LUSTIG, S., SCHOLES, C. J., STEGEMAN, T. J., OUSSEDIK, S., COOLICAN, M. R. J. & PARKER, D. A. 2012. Sagittal placement of the femoral component in total knee arthroplasty predicts knee flexion contracture at one-year follow-up. *International Orthopaedics,* 36**,** 1835-1839.
LUTZNER, C., KIRSCHNER, S. & LUTZNER, J. 2014. Patient activity after TKA depends on patient-specific parameters. *Clinical Orthopaedics & Related Research,* 472**,** 3933-40.
LÜTZNER, J., HARTMANN, A., LÜTZNER, C. & KIRSCHNER, S. 2014. Is range of motion after cruciate-retaining total knee arthroplasty influenced by prosthesis design? A prospective randomized trial. *Journal of arthroplasty,* 29**,** 961-965.
MACAULAY, W., KIM, A. D., GELLER, J. A., NELLANS, K. W., MORRISON, T. A., CHOI, J. K., NYCE, J. & COYLE, R. 2010. The effect of hypovitaminosis D among primary knee arthroplasty patients. *Osteoporosis International,* 1**,** S105-S106.
MACULÉ, F., SASTRE, S., LASURT, S., SALA, P., SEGUR, J. & MALLOFRÉ, C. 2005. Hoffa's fat pad resection in total knee arthroplasty. *Acta orthopaedica belgica,* 71**,** 714-717.
MAFFULLI, G., BRIDGMAN, S. & MAFFULLI, N. 2011. Total knee arthoplasty: better short-term results after subvastus approach. *Knee Surgery, Sports Traumatology, Arthroscopy,* 19**,** 1047; author reply 1048.
MAGALDI, R. J., STAFF, I., STOVALL, A. E., STOHLER, S. A. & LEWIS, C. G. 2019. Impact of resilience on outcomes of total knee arthroplasty. *The Journal of arthroplasty,* 34**,** 2620-2623. e1.
MAHOMED, N. N., LIANG, M. H., COOK, E. F., DALTROY, L. H., FORTIN, P. R., FOSSEL, A. H. & KATZ, J. N. 2002. The importance of patient expectations in predicting functional outcomes after total joint arthroplasty. *Journal of Rheumatology,* 29**,** 1273-9.
MAHONEY, O. M., KINSEY, T. L., D'ERRICO, T. J., SHEN, J., MAHONEY, O. M., KINSEY, T. L., D'ERRICO, T. J. & SHEN, J. 2012. The John Insall Award: no functional advantage of a mobile bearing posterior stabilized TKA. *Clinical Orthopaedics & Related Research,* 470**,** 33-44.
MALVIYA, A., LINGARD, E. A., WEIR, D. J. & DEEHAN, D. J. 2009. Predicting range of movement after knee replacement: the importance of posterior condylar offset and tibial slope. *Knee Surgery, Sports Traumatology, Arthroscopy,* 17**,** 491-8.
MARTINEZ, V., FLETCHER, D., BOUHASSIRA, D., SESSLER, D. I. & CHAUVIN, M. 2007. The evolution of primary hyperalgesia in orthopedic surgery: quantitative sensory testing and clinical evaluation before and after total knee arthroplasty. *Anesthesia & Analgesia,* 105**,** 815-21.
MAT, E. I. M., SHARIFUDIN, M., SHOKRI, A. & AB, R. S. 2016. Preoperative physiotherapy and short-term functional outcomes of primary total knee arthroplasty. *Singapore medical journal,* 57**,** 138-143.
MAUS, U., MARQUES, C. J., SCHEUNEMANN, D., LAMPE, F., LAZOVIC, D., HOMMEL, H., VOGEL, D., HAUNSCHILD, M. & PFITZNER, T. 2017. No improvement in reducing outliers in coronal axis alignment with patient-specific instrumentation. *Knee Surgery, Sports Traumatology, Arthroscopy,* 25**,** 25.
MAXWELL, J., NIU, J., SINGH, J. A., NEVITT, M. C., LAW, L. F. & FELSON, D. 2013. The influence of the contralateral knee prior to knee arthroplasty on post-arthroplasty function: the multicenter osteoarthritis study. *Journal of Bone & Joint Surgery - American Volume,* 95**,** 989-93.
MEESSEN, J., FIOCCO, M., LEICHTENBERG, C. S., VLIET VLIELAND, T. P. M., SLAGBOOM, P. E. & NELISSEN, R. 2018. Frailty Questionnaire Is Not a Strong Prognostic Factor for Functional Outcomes in Hip or Knee Arthroplasty Patients. *Geriatric Orthopaedic Surgery & Rehabilitation,* 10**,** 2151459318808164.
MEHTA, S. & LOTKE, P. A. 2007. Impact of surgeon handedness and laterality on outcomes of total knee arthroplasties: should right-handed surgeons do only right TKAs? *American journal of orthopedics (Belle Mead, N.J.),* 36**,** 530-533.

MEHTA, S., PALAGANAS, M., PERRUCCIO, A. V. & DAVIS, A. M. 2014. Do women have poorer outcomes following total knee replacement for osteoarthritis? *Osteoarthritis and Cartilage,* 1**,** S15-S16.
MEHTA, S. P., PERRUCCIO, A. V., PALAGANAS, M. & DAVIS, A. M. 2015. Do women have poorer outcomes following total knee replacement? *Osteoarthritis & Cartilage,* 23**,** 1476-82.
MEIJERINK, H. J., BROKELMAN, R. B. G., VAN LOON, C. J. M., VAN KAMPEN, A. & DE WAAL MALEFIJT, M. C. 2009. Surgeon's expectations do not predict the outcome of a total knee arthroplasty. *Archives of Orthopaedic and Trauma Surgery,* 129**,** 1361-1365.
MERCURIO, M., GASPARINI, G., CARBONE, E. A., GALASSO, O. & SEGURA-GARCIA, C. 2020. Personality traits predict residual pain after total hip and knee arthroplasty. *International Orthopaedics,* 44**,** 1263-1270.
METSNA, V., VOROBJOV, S., LEPIK, K. & MÄRTSON, A. 2014. Anterior knee pain following total knee replacement correlates with the OARSI score of the cartilage of the patella. *Acta Orthopaedica,* 85**,** 427-432.
MIOZZARI, H. H., SAGAWA JUNIOR, Y., HOFFMEYER, P., SUVA, D., ARMAND, S. & TURCOT, K. 2013. Gait analysis and patients outcome after TKA comparing dependent vs. Independent bone cut technique: A preliminary study. *Swiss Medical Weekly,* 198**,** 2S-3S.
MITTAL, R., KO, V., ADIE, S., NAYLOR, J., DAVE, J., DAVE, C., HARRIS, I. A., HACKETT, D., NGO, D. & DIETSCH, S. 2012. Tourniquet application only during cement fixation in total knee arthroplasty: a double-blind, randomized controlled trial. *ANZ Journal of Surgery,* 82**,** 428-33.
MIZNER, R. L., PETTERSON, S. C., STEVENS, J. E., AXE, M. J. & SNYDER-MACKLER, L. 2005. Preoperative quadriceps strength predicts functional ability one year after total knee arthroplasty. *Journal of Rheumatology,* 32**,** 1533-9.
MOGHTADAEI, M., YEGANEH, A., HOSSEINZADEH, N., KHAZANCHIN, A., MOAIEDFAR, M., JOLFAEI, A. G. & NASIRI, S. 2020. The Impact of Depression, Personality, and Mental Health on Outcomes of Total Knee Arthroplasty. *Clinics in Orthopedic Surgery,* 12**,** 456-463.
MOLT, M. & TOKSVIG-LARSEN, S. 2014. Similar early migration when comparing CR and PS in Triathlon? TKA: a prospective randomised RSA trial. *The knee,* 21**,** 949-954.
MORZE, C. J., JOHNSON, N. R., WILLIAMS, G., MORONEY, M., LAMBERTON, T. & MCAULIFFE, M. 2013. Knee pain during the first three months after unilateral total knee arthroplasty: a multi-centre prospective cohort study. *Journal of Arthroplasty,* 28**,** 1565-70.
MOTWANI, P., JARIWALA, A. & VALENTINE, N. 2013. Does navigation total knee replacement really make a difference? *Journal of Musculoskeletal Research,* 16.
NANDI, M., CORNELIUS, M., CAMPBELL, C., SMITH, M., HAYTHORNTHWAITE, J., WRIGHT, J., EDWARDS, R. & STRICHARTZ, G. 2016. Sex differences in pain and functioning among Total Knee Arthroplasty patients. *Journal of Pain,* 1**,** S1-S2.
NANKAKU, M., ITO, H., FURU, M., KURIYAMA, S., NAKAMURA, S., IKEGUCHI, R. & MATSUDA, S. 2018. Preoperative factors related to the ambulatory status at 1 year after total knee arthroplasty. *Disability & Rehabilitation,* 40**,** 1929-1932.
NAVARRO COLLADO, M. J., PEIRO, S., TRENOR GOMIS, C., RUIZ JARENO, L., PEREZ IGUALADA, A. & GUEROLA SOLER, N. 2000. [Factors related to functional outcomes and quality of life after knee arthroplasty]. *Medicina Clinica,* 114**,** 250-4.
NAYLOR, J. M., HARMER, A. R. & HEARD, R. C. 2008. Severe other joint disease and obesity independently influence recovery after joint replacement surgery: an observational study. *Australian Journal of Physiotherapy,* 54**,** 57-64.
NEOGI, T., NIU, J., FREY-LAW, L., ARENDT-NIELSEN, L., SINGH, J., SCHOLZ, J. & WOOLF, C. 2010. Role of central sensitization in persistent pain post-knee replacement: The MOST study. *Arthritis and Rheumatism,* 10**,** 1385.
NEUBURGER, J., HUTCHINGS, A., BLACK, N. & VAN DER MEULEN, J. H. 2013. Socioeconomic differences in patient-reported outcomes after a hip or knee replacement in the English National Health Service. *Journal of Public Health,* 35**,** 115-24.
NEUPREZ, A., NEUPREZ, A. H., KAUX, J. F., KURTH, W., DANIEL, C., THIRION, T., HUSKIN, J. P., GILLET, P., BRUYERE, O. & REGINSTER, J. Y. 2018. Early Clinically Relevant Improvement in Quality of Life and Clinical Outcomes 1 Year Postsurgery in Patients with Knee and Hip Joint Arthroplasties. *Cartilage,* 9**,** 127-139.
NIELSEN, C. S., NEBERGALL, A., HUDDLESTON, J., KALLEMOSE, T., MALCHAU, H. & TROELSEN, A. 2018. Medial Overhang of the Tibial Component Is Associated With Higher Risk of Inferior Knee Injury and Osteoarthritis Outcome Score Pain After Knee Replacement. *Journal of Arthroplasty,* 33**,** 1394-1398.

NIKI, Y., TAKEDA, Y., HARATO, K. & SUDA, Y. 2015. Factors affecting the achievement of Japanese-style deep knee flexion after total knee arthroplasty using posterior-stabilized prosthesis with high-flex knee design. *Journal of Orthopaedic Science,* 20**,** 1012-8.

NOISEUX, N., CALLAGHAN, J., CLARK, C., ZIMMERMAN, M., SLUKA, K. & RAKEL, B. 2014. Preoperative predictors of pain following total knee arthroplasty. *Journal of arthroplasty,* 29**,** 1383-1387.

NUÑEZ, M., LOZANO, L., NUÑEZ, E., SEGUR, J. M. & SASTRE, S. 2011. Factors Influencing Health-related Quality of Life after TKA in Patients who are Obese. *Clinical Orthopaedics & Related Research,* 469**,** 1148-1153.

NWANKWO, V. C., JIRANEK, W. A., GREEN, C. L., ALLEN, K. D., GEORGE, S. Z. & BETTGER, J. P. 2021. Resilience and pain catastrophizing among patients with total knee arthroplasty: a cohort study to examine psychological constructs as predictors of post-operative outcomes. *Health & Quality of Life Outcomes,* 19**,** 136.

OATIS, C. A., LI, W., ROSAL, M., AYERS, D. & FRANKLIN, P. D. 2012. Associations between body mass index and physical activity following total knee replacement. *Arthritis and Rheumatism,* 10**,** S1123-S1124.

OBERBEK, J. & SYNDER, M. 2015. Impact of Body Mass Index (BMI) on Early Outcomes of Total Knee Arthroplasty. *Ortopedia Traumatologia Rehabilitacja,* 17**,** 127-34.

OKAMOTO, S., OKAZAKI, K., MITSUYASU, H., MATSUDA, S., MIZU-UCHI, H., HAMAI, S., TASHIRO, Y. & IWAMOTO, Y. 2014. Extension gap needs more than 1-mm laxity after implantation to avoid post-operative flexion contracture in total knee arthroplasty. *Knee Surgery, Sports Traumatology, Arthroscopy,* 22**,** 3174-80.

OTERO, J. E., GRAVES, C. M., GAO, Y., OLSON, T. S., DICKINSON, C. C., CHALUS, R. J., VITTETOE, D. A., GOETZ, D. D. & CALLAGHAN, J. J. 2016. Patient-Reported Allergies Predict Worse Outcomes After Hip and Knee Arthroplasty: Results From a Prospective Cohort Study. *Journal of Arthroplasty,* 31**,** 2746-2749.

OZDEMIR, M., DEMIRKALE, I., SESEN, H., TASKESEN, A., OKKAOGLU, M. C. & ALTAY, M. 2017. Affective temperament does not influence satisfaction after total knee arthroplasty. *Medicine,* 96**,** e6852.

PAGE, M. G., KATZ, J., CURTIS, K., FUSS, S., COHEN, N., ESCOBAR, E. M. R. & CLARKE, H. 2014. Not all post-operative pain is alike: A pain trajectory analysis in total knee arthroplasty patients. *Pain Research and Management,* 19**,** e90.

PAGE, M. G., KATZ, J., ROMERO ESCOBAR, E. M., LUTZKY-COHEN, N., CURTIS, K., FUSS, S. & CLARKE, H. A. 2015. Distinguishing problematic from nonproblematic postsurgical pain: a pain trajectory analysis after total knee arthroplasty. *Pain,* 156**,** 460-468.

PAN, X., WANG, J., LIN, Z., DAI, W. & SHI, Z. 2019. Depression and Anxiety Are Risk Factors for Postoperative Pain-Related Symptoms and Complications in Patients Undergoing Primary Total Knee Arthroplasty in the United States. *Journal of Arthroplasty.*

PAPAKOSTIDOU, I., DAILIANA, Z. H., PAPAPOLYCHRONIOU, T., LIAROPOULOS, L., ZINTZARAS, E., KARACHALIOS, T. S. & MALIZOS, K. N. 2012. Factors affecting the quality of life after total knee arthroplasties: a prospective study. *BMC Musculoskeletal Disorders,* 13**,** 116.

PARSLEY, B. S., BERTOLUSSO, R., HARRINGTON, M., BREKKE, A. & NOBLE, P. C. 2010. Influence of gender on age of treatment with TKA and functional outcome. *Clinical Orthopaedics & Related Research,* 468**,** 1759-64.

PAXTON, E. W., TORRES, A., LOVE, R. M., BARBER, T. C., SHETH, D. S. & INACIO, M. C. S. 2016. Total joint replacement: A multiple risk factor analysis of physical activity level 1–2 years postoperatively. *Acta Orthopaedica,* 87**,** 44-49.

PEREIRA, D. L., MELEIRO, H. L., CORREIA, I. A. & FONSECA, S. 2016. [Pain after major elective orthopedic surgery of the lower limb and type of anesthesia: does it matter?]. *Revista Brasileira de Anestesiologia,* 66**,** 628-636.

PERRUCCIO, A. V., BADLEY, E. M., HOGG-JOHNSON, S. & DAVIS, A. M. 2010a. Characterizing self-rated health during a period of changing health status. *Social Science & Medicine,* 71**,** 1636-43.

PERRUCCIO, A. V., BADLEY, E. M., HOGG-JOHNSON, S. & DAVIS, A. M. 2010b. Characterizing self-rated health during a period of changing health status. *Social Science & Medicine,* 71**,** 1636-1643.

PERRUCCIO, A. V., DAVIS, A. M., HOGG-JOHNSON, S. & BADLEY, E. M. 2011a. Importance of self-rated health and mental well-being in predicting health outcomes following total joint replacement surgery for osteoarthritis. *Arthritis care & research,* 63**,** 973-81.

PERRUCCIO, A. V., FITZPATRICK, J., POWER, J. D., GANDHI, R., RAMPERSAUD, Y. R., MAHOMED, N. N., DAVEY, J. R., SYED, K., VEILLETTE, C. & BADLEY, E. M. 2019. The effects of depression, low

back pain and comorbidities on pain after total knee arthroplasty for osteoarthritis are modified by sex. *Arthritis care & research*.
PERRUCCIO, A. V., POWER, J. D., BADLEY, E. M., GANDHI, R., MAHOMED, N. N. & DAVIS, A. M. 2011b. Outcomes following total knee replacement surgery for OA: There is more than just the knee to consider. *Osteoarthritis and Cartilage,* 1**,** S22.
PETERSEN, K. K., ARENDT-NIELSEN, L., SIMONSEN, O., WILDER-SMITH, O. & LAURSEN, M. B. 2015. Presurgical assessment of temporal summation of pain predicts the development of chronic postoperative pain 12 months after total knee replacement. *Pain,* 156**,** 55-61.
PETERSEN, K. K., ARENDT-NIELSEN, L., VELA, J., SKOU, S. T., ELD, M., AL-MASHKUR, N. M., BOESEN, M., RIIS, R. G. C. & SIMONSEN, O. 2020. Less Severe Preoperative Synovitis is Associated With Higher Self-reported Pain Intensity 12 Months After Total Knee Arthroplasty-An Exploratory Prospective Observational Study. *Clin J Pain,* 36**,** 34-40.
PETERSEN, K. K., SIMONSEN, O., LAURSEN, M. B. & ARENDT-NIELSEN, L. 2017. The Role of Preoperative Radiological Severity, Sensory Testing, and Temporal Summation on Chronic Postoperative Pain following Total Knee Arthroplasty. *Clinical Journal of Pain.,* 16.
PINSORNSAK, P., NARATRIKUN, K. & CHUMCHUEN, S. 2014. The effect of infrapatellar fat pad excision on complications after minimally invasive TKA: a randomized controlled trial. 472.
PINTO, P., MCINTYRE, T., FERRERO, R., ALMEIDA, A. & ARAUJO-SOARES, V. 2014. Risk factors for moderate and severe persistent pain in patients undergoing total knee and hip arthroplasty: A prospective predictive study. *Pain Practice,* 1**,** 94-95.
PINTO, P. R., MCINTYRE, T., FERRERO, R., ALMEIDA, A. & ARAUJO-SOARES, V. 2013. Risk factors for moderate and severe persistent pain in patients undergoing total knee and hip arthroplasty: a prospective predictive study. *PLoS ONE [Electronic Resource],* 8**,** e73917.
POLKOWSKI, I. G. G., RUH, E. L., BARRACK, T. N., NUNLEY, R. M. & BARRACK, R. L. 2013. Is pain and dissatisfaction after TKA related to early-grade preoperative osteoarthritis? Knee. *Clinical Orthopaedics and Related Research,* 471**,** 162-168.
PONT, C. P., ORTIZ, V. A. D. C., PASTOR, B. S., CANO, L. G., MESQUIDA, M. E. P., COBO, E. P., MARQUES, M. C. & GONZÁLEZ, M. B. 2011. Predictive factors of the functional level after total knee arthroplasty. *Rehabilitacion,* 45**,** 240-246.
PUA, Y., ONG, P., CLARK, R., LEE, A., TAN, J. & BRYANT, A. 2012. A prediction model for fear-induced activity limitation after total knee arthroplasty: a prospective cohort study. *Proceedings of singapore healthcare,* 21**,** S354.
PUA, Y. H., ONG, P. H., CHONG, H. C., YEO, W., TAN, C. & LO, N. N. 2013. Knee extension range of motion and self-report physical function in total knee arthroplasty: mediating effects of knee extensor strength. *BMC Musculoskeletal Disorders,* 14**,** 33.
PUA, Y. H., SEAH, F. J., CLARK, R. A., LIAN-LI POON, C., TAN, J. W. & CHONG, H. C. 2017. Factors associated with gait speed recovery after total knee arthroplasty: A longitudinal study. *Semin Arthritis Rheum,* 46**,** 544-551.
PUA, Y. H., SEAH, F. J., SEET, F. J., TAN, J. W., LIAW, J. S. & CHONG, H. C. 2015. Sex Differences and Impact of Body Mass Index on the Time Course of Knee Range of Motion, Knee Strength, and Gait Speed After Total Knee Arthroplasty. *Arthritis care & research,* 67**,** 1397-405.
QUINTANA, J. M., ESCOBAR, A., AROSTEGUI, I., BILBAO, A., AZKARATE, J., GOENAGA, J. I. & ARENAZA, J. C. 2006. Health-related quality of life and appropriateness of knee or hip joint replacement. *Archives of Internal Medicine,* 166**,** 220-6.
RADMER, S., ANDRESEN, R. & SPARMANN, M. 2006. Simultaneous bilateral total knee arthroplasty in patients with rheumatoid arthritis. *Zeitschrift fur orthopadie und ihre grenzgebiete,* 144**,** 472-476.
RAJAMAKI, T., JAMSEN, E., PUOLAKKA, P., NEVALAINEN, P. & MOILANEN, T. 2015. Diabetes is associated with persistent pain after hip and knee replacement. 86.
RAKEL, B., NOISEUX, N., ZIMMERMAN, B., CALLAGHAN, J., CLARK, C. & SLUKA, K. 2013. Predictors of pain following total knee arthroplasty. *Journal of Pain,* 1**,** S15.
RAMAESH, R., JENKINS, P., LANE, J. V., KNIGHT, S., MACDONALD, D. & HOWIE, C. 2014. Personality, function and satisfaction in patients undergoing total hip or knee replacement. *Journal of Orthopaedic Science,* 19**,** 275-81.
RAZMJOU, H., BOLJANOVIC, D., WRIGHT, S., MURNAGHAN, J. & HOLTBY, R. 2015. Association between Neuropathic Pain and Reported Disability after Total Knee Arthroplasty. *Physiotherapy Canada,* 67**,** 311-318.

REID, M. J., BOOTH, G., KHAN, R. J. & JANES, G. 2014. Patellar eversion during total knee replacement: a prospective, randomized trial. *Journal of Bone & Joint Surgery - American Volume,* 96**,** 207-13.
RICE, D., KLUGER, M., MCNAIR, P., LEWIS, G., SOMOGYI, A., BOROTKANICS, R., BARRATT, D. & WALKER, M. 2018. Persistent postoperative pain after total knee arthroplasty: a prospective cohort study of potential risk factors. *British journal of anaesthesia,* 121**,** 804.
RICHARDS, J., PEJSA, M., HAND, M., CORNELIUS, M., CAMPBELL, C., HAYTHORNTHWAITE, J., EDWARDS, R. & SMITH, M. 2016. Psychometric evaluation and predictive validity of the sleep and pain behaviors survey in knee osteoarthritis patients undergoing total knee replacement. *Journal of Pain,* 1**,** S17.
RIDDLE, D. L. 2018. Prevalence and Predictors of Symptom Resolution and Functional Restoration in the Index Knee After Knee Arthroplasty: A Longitudinal Study. *Archives of Physical Medicine & Rehabilitation,* 99**,** 887-892.
RIDDLE, D. L., PERERA, R. A., JIRANEK, W. A. & DUMENCI, L. 2015. Using surgical appropriateness criteria to examine outcomes of total knee arthroplasty in a United States sample. *Arthritis Care & Research,* 67**,** 349-357.
RIDDLE, D. L., SLOVER, J., KEEFE, F. J., ANG, D., DUMENCI, L. & PERERA, R. A. 2020. Racial differences in pain and function following knee arthroplasty: a secondary analysis from a multicenter randomized clinical trial. *Osteoarthritis and cartilage,* 28**,** S431-S432.
RIDDLE, D. L., WADE, J. B., JIRANEK, W. A. & KONG, X. 2009. Preoperative pain catastrophizing predicts pain outcome following knee arthroplasty. *Osteoarthritis and Cartilage,* 1**,** S156.
ROSEN, A. S., NEVILLE, L., PULIDO, P. A., PATIL, S., WALKER, R. H. & COP, S. N. 2013. Outcome and range of motion using a high-flexion cruciate-retaining TKA. *Orthopedics,* 36**,** e1198-e1202.
RUSSELL, R. D., HUO, M. H., DE JONG, L. & JONES, R. E. 2014. Preoperative flexion does not influence postoperative flexion after rotating-platform total knee arthroplasty. *Knee Surgery, Sports Traumatology, Arthroscopy,* 22**,** 1644-8.
SAKELLARIOU, V. I., POULTSIDES, L. A., YAN, M. A., BAE, J., LIU, S., SCULCO, T. P. & MA, Y. 2016. Risk Assessment for Chronic Pain and Patient Satisfaction After Total Knee Arthroplasty. *Orthopedics,* 39**,** 55-62.
SALAZAR, Y., RODRIGUEZ, U., DURSTELER, C., PUIG, L., MONTES, A. & ESCOLANO, F. 2013. Painful knee prosthesis: Relationship between endogenous analgesia and persistent post-surgical pain: Preliminary data. *European Journal of Anaesthesiology,* 51**,** 205-206.
SANCHEZ-SANTOS, M. T., GARRIGA, C., JUDGE, A., BATRA, R. N., PRICE, A. J., LIDDLE, A. D., JAVAID, M. K., COOPER, C., MURRAY, D. W. & ARDEN, N. K. 2018. Development and validation of a clinical prediction model for patient-reported pain and function after primary total knee replacement surgery. *Scientific Reports,* 8**,** 3381.
SANCHEZ-SANTOS, M. T., JUDGE, A., BATRA, R. N., MURRAY, D., PRICE, A., LIDDLE, A. D., JAVAID, M. K., COOPER, C. & ARDEN, N. K. 2014. A clinical tool for the prediction of patient-reported outcomes after knee replacement surgery. *Osteoarthritis and Cartilage,* 1**,** S412.
SCHAUMBURGER, J., LECHLER, P., RIEDT, S., SPRINGORUM, H. R., RATH, B., BAIER, C., KOCK, F. X., GRIFKA, J. & HANDEL, M. 2012. [Patient satisfaction and muscle torque after total knee replacement in dependence on body mass index]. [German]. *Zeitschrift fur Orthopadie und Unfallchirurgie,* 150**,** 641-647.
SCHWARTZ, I., KANDEL, L., SAJINA, A., LITINEZKI, D., HERMAN, A. & MATTAN, Y. 2012. Balance is an important predictive factor for quality of life and function after primary total knee replacement. *Journal of Bone & Joint Surgery - British Volume,* 94**,** 782-786.
SCOTT, C. E., BUGLER, K. E., CLEMENT, N. D., MACDONALD, D., HOWIE, C. R. & BIANT, L. C. 2012. Patient expectations of arthroplasty of the hip and knee. *Journal of Bone & Joint Surgery - British Volume,* 94**,** 974-81.
SCOTT, C. E. H., HOWIE, C. R., MACDONALD, D. & BIANT, L. C. 2010. Predicting dissatisfaction following total knee replacement: A prospective study of 1217 patients. *Journal of Bone and Joint Surgery - Series B,* 92**,** 1253-1258.
SEOL, J. H., SEON, J. K. & SONG, E. K. 2016. Comparison of postoperative complications and clinical outcomes between simultaneous and staged bilateral total knee arthroplasty. *Journal of Orthopaedic Science,* 21**,** 766-769.
SHARMA, S., KUMAR, V., SOOD, M. & MALHOTRA, R. 2021. Effect of Preoperative Modifiable Psychological and Behavioural Factors on Early Outcome Following Total Knee Arthroplasty in an Indian Population. *Indian Journal of Orthopaedics,* 55**,** 939-947.

© 2022 Olsen U et al. *JAMA Network Open.*

SHIM, J., MCLERNON, D. J., HAMILTON, D., SIMPSON, H. A., BEASLEY, M. & MACFARLANE, G. J. 2018. Development of a clinical risk score for pain and function following total knee arthroplasty: results from the TRIO study. *Rheumatol Adv Pract,* 2**,** rky021.
SINGH, G., HAN, F., KAKI, R. R., SHEN, L. & NATHAN, S. S. 2015. Does Limited Tourniquet Usage in Primary Total Knee Arthroplasty Result in Better Functional Outcomes? *Annals of the Academy of Medicine, Singapore,* 44**,** 302-306.
SIVIERO, P., MARSEGLIA, A., BIZ, C., ROVINI, A., RUGGIERI, P., NARDACCHIONE, R. & MAGGI, S. 2020. Quality of life outcomes in patients undergoing knee replacement surgery: longitudinal findings from the QPro-Gin study. *BMC Musculoskelet Disord,* 21**,** 436.
SLEVIN, O., AMSLER, F. & HIRSCHMANN, M. T. 2017. No correlation between coronal alignment of total knee arthroplasty and clinical outcomes: a prospective clinical study using 3D-CT. *Knee Surgery, Sports Traumatology, Arthroscopy,* 25**,** 3892-3900.
SMITH, A. J., LLOYD, D. G. & WOOD, D. J. 2006. A kinematic and kinetic analysis of walking after total knee arthroplasty with and without patellar resurfacing. *Clinical Biomechanics,* 21**,** 379-86.
SMITH, J. R. A., MATHEWS, J. A., OSBORNE, L., BAKEWELL, Z. & WILLIAMS, J. L. 2019. Why do patients not kneel after total knee replacement? Is neuropathic pain a contributing factor? *Knee,* 26**,** 427-434.
SMITH, J. W., MARCUS, R. L., PETERS, C. L., PELT, C. E., TRACY, B. L. & LASTAYO, P. C. 2014. Muscle force steadiness in older adults before and after total knee arthroplasty. *Journal of Arthroplasty,* 29**,** 1143-8.
SMITH, J. W., MARTINS, T. B., GOPEZ, E., JOHNSON, T., HILL, H. R. & ROSENBERG, T. D. 2012. Significance of C-reactive protein in osteoarthritis and total knee arthroplasty outcomes. *Therapeutic Advances in Musculoskeletal Disease,* 4**,** 315-25.
SONI, A., ARDEN, N. K., PRICE, A. J., TRACEY, I. & JAVAID, M. 2016. Neuropathic pain as a predictor of short and long-term outcome following knee replacement surgery. *Osteoarthritis and Cartilage,* 1**,** S420-S421.
SONI, A., GWYLIM, S., ARDEN, N., TRACEY, I., PRICE, A. & JAVAID, M. 2014. Pre-operative experimental thermal sensitivity predicts oxford knee score 1-year post-operatively: A preliminary study. *Osteoarthritis and Cartilage,* 1**,** S414-S415.
STICKLES, B., PHILLIPS, L., BROX, W. T., OWENS, B. & LANZER, W. L. 2001. Defining the relationship between obesity and total joint arthroplasty. *Obesity Research,* 9**,** 219-23.
STONE, O. D., DUCKWORTH, A. D., CURRAN, D. P., BALLANTYNE, J. A. & BRENKEL, I. J. 2017. Severe arthritis predicts greater improvements in function following total knee arthroplasty. *Knee Surgery, Sports Traumatology, Arthroscopy,* 25**,** 2573-2579.
STRATFORD, P. W., KENNEDY, D. M. & ROBARTS, S. F. 2010. Modelling knee range of motion post arthroplasty: clinical applications. *Physiotherapy Canada,* 62**,** 378-87.
STREET, B. D., ADKIN, A. & GAGE, W. 2018. Reported balance confidence and movement reinvestment of younger knee replacement patients are more like younger healthy individuals, than older patients. *Gait & Posture,* 61**,** 130-134.
SVEIKATA, T., PORVANECKAS, N., KANOPA, P., MOLYTE, A., KLIMAS, D., UVAROVAS, V. & VENALIS, A. 2017. Age, Sex, Body Mass Index, Education, and Social Support Influence Functional Results After Total Knee Arthroplasty. *Geriatric Orthopaedic Surgery & Rehabilitation,* 8**,** 71-77.
TABUTIN, J., BANON, F., CATONNE, Y., GROBOST, J., TESSIER, J. L. & TILLIE, B. 2005. Should we resurface the patella in total knee replacement? Experience with the Nex Gen prothesis. *Knee Surgery, Sports Traumatology, Arthroscopy,* 13**,** 534-8.
TAN, S. C., CHAN, Y. H., CHONG, H. C., CHIN, P. L., YEW, A., CHIA, S. L., TAY, D., LO, N. N. & YEO, S. J. 2014. Association of surgeon factors with outcome scores after total knee arthroplasty. *Journal of Orthopaedic Surgery,* 22**,** 378-82.
TCHETINA, E. V., GLEMBA, K. E., MARKOVA, G. A., NARYSHKIN, E. A., TASKINA, E. A., MAKAROV, M. A. & LILA, A. M. 2020. Development of Postoperative Pain in Patients with End-Stage Knee Osteoarthritis Is Associated with Upregulation of Genes Related to Extracellular Matrix Degradation, Inflammation, and Apoptosis Measured in the Peripheral Blood before Knee Surgery. *Life (Basel),* 10**,** 30.
THOMAZEAU, J., ROUQUETTE, A., MARTINEZ, V., RABUEL, C., PRINCE, N., LAPLANCHE, J. L., NIZARD, R., BERGMANN, J. F., PERROT, S. & LLORET-LINARES, C. 2016. Predictive Factors of Chronic Post-Surgical Pain at 6 Months Following Knee Replacement: Influence of Postoperative Pain Trajectory and Genetics. *Pain Physician,* 19**,** E729-41.
TILBURY, C., HOLTSLAG, M. J., TORDOIR, R. L., LEICHTENBERG, C. S., VERDEGAAL, S. H., KROON, H. M., FIOCCO, M., NELISSEN, R. G. & VLIET VLIELAND, T. P. 2016. Outcome of total hip arthroplasty,

but not of total knee arthroplasty, is related to the preoperative radiographic severity of osteoarthritis. A prospective cohort study of 573 patients. *Acta Orthopaedica,* 87**,** 67-71.
TILBURY, C., HAANSTRA, T. M., VERDEGAAL, S. H. M., NELISSEN, R., DE VET, H. C. W., VLIET VLIELAND, T. P. M. & OSTELO, R. W. 2018. Patients' pre-operative general and specific outcome expectations predict postoperative pain and function after total knee and total hip arthroplasties. *Scand J Pain,* 18**,** 457-466.
TOGUCHI, K., NAKAJIMA, A., AKATSU, Y., SONOBE, M., YAMADA, M., TAKAHASHI, H., SAITO, J., AOKI, Y., SUGURO, T. & NAKAGAWA, K. 2020. Predicting clinical outcomes after total knee arthroplasty from preoperative radiographic factors of the knee osteoarthritis. *BMC Musculoskelet Disord,* 21**,** 9.
TOLK, J. J., JANSSEN, R. P. A., HAANSTRA, T. M., VAN DER STEEN, M. C., BIERMA-ZEINSTRA, S. M. A. & REIJMAN, M. 2021. The influence of expectation modification in knee arthroplasty on satisfaction of patients: a randomized controlled trial. *Bone Joint J,* 103-B**,** 619-626.
TRACE, R. 2006. Morbidly obese TKA patients fare poorly, have less function, more complications. *Orthopedics Today,* 26**,** 8-8.
TWIGGS, J. G., WAKELIN, E. A., FRITSCH, B. A., LIU, D. W., SOLOMON, M. I., PARKER, D. A., KLASAN, A. & MILES, B. P. 2019. Clinical and Statistical Validation of a Probabilistic Prediction Tool of Total Knee Arthroplasty Outcome. *Journal of Arthroplasty.*
UTRILLAS-COMPAIRED, A., DE LA TORRE-ESCUREDO, B. J., TEBAR-MARTINEZ, A. J. & ASUNSOLO-DEL BARCO, A. 2014. Does preoperative psychologic distress influence pain, function, and quality of life after TKA? *Clinical Orthopaedics & Related Research,* 472**,** 2457-65.
VAEGTER, H. B., HANDBERG, G., EMMELUTH, C. & GRAVEN-NIELSEN, T. 2017. Preoperative Hypoalgesia After Cold Pressor Test and Aerobic Exercise is Associated With Pain Relief 6 Months After Total Knee Replacement. *Clinical Journal of Pain,* 33**,** 475-484.
VAN DEN AKKER-SCHEEK, I., STEVENS, M., GROOTHOFF, J. W., BULSTRA, S. K. & ZIJLSTRA, W. 2007. Preoperative or postoperative self-efficacy: which is a better predictor of outcome after total hip or knee arthroplasty? *Patient Education & Counseling,* 66**,** 92-9.
VAN HAMERSVELD, K. T., MARANG-VAN DE MHEEN, P. J., NELISSEN, R. & TOKSVIG-LARSEN, S. 2018. Migration of all-polyethylene compared with metal-backed tibial components in cemented total knee arthroplasty. *Acta Orthopaedica***,** 1-6.
VAN LOON, C., BAAS, N., HUEY, V., LESKO, J., MEERMANS, G. & VERGROESEN, D. 2021. Early outcomes and predictors of patient satisfaction after TKA: a prospective study of 200 cases with a contemporary cemented rotating platform implant design. *J Exp Orthop,* 8**,** 30.
VAN ONSEM, S., VERSTRAETE, M., DHONT, S., ZWAENEPOEL, B., VAN DER STRAETEN, C. & VICTOR, J. 2018. Improved walking distance and range of motion predict patient satisfaction after TKA. *Knee Surgery, Sports Traumatology, Arthroscopy,* 8**,** 08.
VEKAMA, L., PUOLAKKA, T., HONKASALO, M., HUHTALA, H., MOILANEN, T. & JÄMSEN, E. 2015. Functional gain following knee replacement in patients aged 75 and older: a prospective follow-up study. *Aging Clinical & Experimental Research,* 27**,** 865-876.
VELA, J., KJAER PETERSEN, K., ARENDT-NIELSEN, L., MEYER ANDERSEN, M. & SIMONSEN, O. 2017. Preoperative synovitis in knee osteoarthritis is predictive for pain 1 year after total knee arthroplasty. *Scandinavian Journal of Pain,* 16**,** 184.
VILA, M. R., TODOROVIC, M. S., TANG, C., FISHER, M., STEINBERG, A., FIELD, B., BOTTROS, M. M., AVIDAN, M. S. & HAROUTOUNIAN, S. 2020. Cognitive flexibility and persistent post-surgical pain: the FLEXCAPP prospective observational study. *Br J Anaesth,* 124**,** 614-622.
VINA, E. R., HANNON, M. J. & KWOH, C. K. 2014. Improvement following total knee replacement (TKR) surgery: Exploring preoperative symptoms and change in preoperative symptoms. *Arthritis and Rheumatology,* 10**,** S1297.
VINA, E. R., HANNON, M. J. & KWOH, C. K. 2016. Improvement following total knee replacement surgery: Exploring preoperative symptoms and change in preoperative symptoms. *Seminars in Arthritis & Rheumatism,* 45**,** 547-55.
VOGEL, M., RIEDIGER, C., KRIPPL, M., FROMMER, J., LOHMANN, C. & ILLIGER, S. 2019. Negative Affect, Type D Personality, Quality of Life, and Dysfunctional Outcomes of Total Knee Arthroplasty. *Pain Res Manag,* 2019**,** 6393101.

WADA, O., NAGAI, K., HIYAMA, Y., NITTA, S., MARUNO, H. & MIZUNO, K. 2016. Diabetes is a Risk Factor for Restricted Range of Motion and Poor Clinical Outcome After Total Knee Arthroplasty. *Journal of Arthroplasty,* 31**,** 1933-7.

WALKER, J., CAMPBELL, C., PEJSA, M., HAND, M., HAYTHORNTHWAITE, J., EDWARDS, R., KHANUJA, P., STERLING, R., CORNELIUS, M. & SMITH, M. 2015. Pre-and post-operative sleep is associated with postoperative pain and functioning following total knee replacements. *Journal of Pain,* 1**,** S63.

WENJUN, L., AYERS, D. C., LEWIS, C. G., BOWEN, T. R., ALLISON, J. J., FRANKLIN, P. D. & LI, W. 2017. Functional Gain and Pain Relief After Total Joint Replacement According to Obesity Status. *Journal of Bone & Joint Surgery, American Volume,* 99**,** 1183-1189.

WIDMER, B. J., SCHOLES, C. J., LUSTIG, S., CONRAD, L., OUSSEDIK, S. I. & PARKER, D. A. 2013. Intraoperative computer navigation parameters are poor predictors of function 1 year after total knee arthroplasty. *Journal of Arthroplasty,* 28**,** 56-61.

WILLIAMS, D. P., PRICE, A. J., BEARD, D. J., HADFIELD, S. G., ARDEN, N. K., MURRAY, D. W. & FIELD, R. E. 2013. The effects of age on patient-reported outcome measures in total knee replacements. *Bone & Joint Journal,* 95**,** 38-44.

WINTERS, J. D., CHRISTIANSEN, C. L. & STEVENS-LAPSLEY, J. E. 2014. Preliminary investigation of rate of torque development deficits following total knee arthroplasty. *Knee,* 21**,** 382-6.

WOHLRAB, D., DITL, J., HERRSCHELMANN, R., SCHIETSCH, U., HEIN, W. & HUBE, R. 2005. [Does the NexGen LPS flex mobile knee prosthesis offer advantages compared to the NexGen LPS?--a comparison of clinical and radiological results]. *Zeitschrift fur Orthopadie und Ihre Grenzgebiete,* 143**,** 567-72.

WOLLMERSTEDT, N., GLATZEL, M., KIRSCHNER, S., SCHNEIDER, J., FALLER, H. & KÖNIG, A. 2006. Comparative analysis of patient-centered outcome of total hip and knee arthroplasty. *Zeitschrift fur orthopadie und ihre grenzgebiete,* 144**,** 464-471.

WOO, Y., LEE, H. & KIM, J. 2006. Effectiveness of Patellar Replacementin Total Knee Arthroplasty: randomized, Prospective Study using Simultaneous Bilateral Total Knee Arthroplasty. *Journal of the korean orthopaedic association,* 41**,** 675-679.

WOOD, T. J., GAZENDAM, A. M., KABALI, C. B. & HAMILTON ARTHROPLASTY, G. 2021. Postoperative Outcomes Following Total Hip and Knee Arthroplasty in Patients with Pain Catastrophizing, Anxiety, or Depression. *J Arthroplasty,* 36**,** 1908-1914.

WRIGHT, D., HOANG, M., SOFINE, A., SILVA, J. P. & SCHWARZKOPF, R. 2017. Pain catastrophizing as a predictor for postoperative pain and opiate consumption in total joint arthroplasty patients. *Archives of Orthopaedic and Trauma Surgery,* 137**,** 1623-1629.

WYLDE, V., PALMER, S., LEARMONTH, I. D. & DIEPPE, P. 2013. The association between pre-operative pain sensitisation and chronic pain after knee replacement: an exploratory study. *Osteoarthritis & Cartilage,* 21**,** 1253-6.

WYLDE, V., SAYERS, A., LENGUERRAND, E., GOOBERMAN-HILL, R., PYKE, M., BESWICK, A. D., DIEPPE, P. & BLOM, A. W. 2015. Preoperative widespread pain sensitization and chronic pain after hip and knee replacement: A cohort analysis. *Pain,* 156**,** 47-54.

WYLDE, V., TRELA-LARSEN, L., WHITEHOUSE, M. R. & BLOM, A. W. 2017. Preoperative psychosocial risk factors for poor outcomes at 1 and 5 years after total knee replacement: A cohort study of 266 patients. *Acta Orthopaedica,* 88**,** 530-536.

XU, J., TWIGGS, J., PARKER, D. & NEGUS, J. 2020. The Association Between Anxiety, Depression, and Locus of Control With Patient Outcomes Following Total Knee Arthroplasty. *J Arthroplasty,* 35**,** 720-724.

YAKOBOV, E., STANISH, W., TANZER, M., DUNBAR, M., RICHARDSON, G. & SULLIVAN, M. J. L. 2018. The prognostic value of pain catastrophizing in health-related quality of life judgments after Total knee arthroplasty. *Health & Quality of Life Outcomes,* 16**,** 126.

YAP, Y. Y. W., EDWARDS, K. L., SOUTAKBAR, H., FERNANDES, G. S. & SCAMMELL, B. E. 2021. Oxford knee score 1 year after TKR for osteoarthritis with reference to a normative population: What can patients expect? *Osteoarthritis and Cartilage Open,* 3.

YAU, W. P., CHIU, K. Y., TANG, W. M. & NG, T. P. 2005. Residual posterior femoral condyle osteophyte affects the flexion range after total knee replacement. *International Orthopaedics,* 29**,** 375-9.

YONG-HAO, P., JIE-TING SEAH, F., CLARK, R. A., LIAN-LI POON, C., WEI-MING TAN, J., HWEI-CHI, C., PUA, Y.-H., SEAH, F. J.-T., POON, C. L.-L., TAN, J. W.-M. & CHONG, H.-C. 2016. Development of a Prediction Model to Estimate the Risk of Walking Limitations in Patients with Total Knee Arthroplasty. *Journal of Rheumatology,* 43**,** 419-426.

© 2022 Olsen U et al. *JAMA Network Open.*

YOUNG-SHAND, K. L., DUNBAR, M. J. & ASTEPHEN WILSON, J. L. 2020. Individual Gait Features Are Associated with Clinical Improvement After Total Knee Arthroplasty. *JB JS Open Access,* 5**,** e0038.
YOUNG, S., WALKER, M., BAYAN, A., BRIANT-EVANS, T., PAVLOU, P., FARRINGTON, B., YOUNG, S. W. & WALKER, M. L. 2017. The Chitranjan S. Ranawat Award : No Difference in 2-year Functional Outcomes Using Kinematic versus Mechanical Alignment in TKA: A Randomized Controlled Clinical Trial. *Clinical Orthopaedics & Related Research,* 475**,** 9-20.
ZENI, J. A., JR. & SNYDER-MACKLER, L. 2010. Preoperative predictors of persistent impairments during stair ascent and descent after total knee arthroplasty. *Journal of Bone & Joint Surgery - American Volume,* 92**,** 1130-6.

© 2022 Olsen U et al. *JAMA Network Open.*
